# Multi-Electron Transfer in Zinc-Based Batteries: Unraveling Redox Mechanisms Toward Next-Generation Self-Powered Technologies

**DOI:** 10.1007/s40820-026-02329-y

**Published:** 2026-10-26

**Authors:** Chao Zhou, Jiaju Zhou, Xiaojun Guo, Haoxiang Zhang, Sajian Wu, Xiaoyu Yu, Xudong Li, Dexu Zheng, Hao Du, Wei Song, Yuanming Gao, Hanying Wang, Dong Yang, Xianyong Wu, Dongliang Chao, Shengzhong Frank Liu

**Affiliations:** 1 State Key Laboratory of Photoelectric Conversion and Utilization of Solar Energy, Dalian National Lab for Clean Energy, Dalian Institute of Chemical Physics, Center of Materials Science and Optoelectronics Engineering, University of Chinese Academy of Sciences, Dalian, 116023 People’s Republic of China; 2 Laboratory of Advanced Materials, Aqueous Battery Center, Shanghai Key Laboratory of Molecular Catalysis and Innovative Materials, Electron Microscope Center of Fudan University, Faculty of Chemistry and Materials, Fudan University, Shanghai, 200433 People’s Republic of China; 3 Department of Chemistry, University of Puerto Rico-Rio Piedras Campus, San Juan, PR 00925 USA; 4 Key Laboratory of Applied Surface and Colloid Chemistry, National Ministry of Education, Shaanxi Engineering Lab for Advanced Energy Technology, School of Materials Science and Engineering, Shaanxi Normal University, Xi’an, 710119 People’s Republic of China; 5 School of New Energy, Yulin University, Yulin, 719000 People’s Republic of China; 6 China National Nuclear Power Co., Ltd., Beijing, 100097 People’s Republic of China

**Keywords:** Multi-electron transfer, Zinc-based batteries, Photo-rechargeable, Overall energy conversion efficiency

## Abstract

The rapid expansion of autonomous IoT networks and wearable electronics has intensified the demand for photo-rechargeable zinc-based batteries (PRZBBs). While realizing high overall energy conversion efficiency intrinsically relies on cathode materials capable of multi-electron transfer reactions, current literature largely treats the fundamental electrochemistry of aqueous zinc batteries and the macroscopic engineering of solar-integrated devices as isolated domains. This review bridges this critical gap, offering a unique positioning by establishing a unified analytical framework that directly links fundamental multi-electron redox mechanisms to the dynamic architectural requirements of PRZBBs. Moving beyond a conventional cataloging of material classes, we critically evaluate diverse cathode platforms, including transition metal oxides, Prussian blue analogues, organics, halogens, and sulfur. Our incremental contribution lies in decoupling how specific multi-step reaction kinetics, distinct voltage plateaus, and dominant charge carriers dictate solar cell coupling strategies, particularly concerning maximum power point matching and intermittent charging adaptability. Furthermore, we critically and systematically assess the practical bottlenecks of these chemistries, such as shuttle effects, kinetic limitations, and voltage hysteresis, that hinder scalable integration. Finally, we distill these insights into a prioritized, roadmap-oriented perspective, outlining the short- to long-term engineering and scientific milestones necessary to translate fundamental multi-electron zinc chemistry into next-generation, commercially viable self-powered technologies.

## Highlights

- A unified analytical framework is established that directly links multi-electron redox mechanisms of diverse cathode chemistries with the dynamic device-level requirements for photo-rechargeable zinc-based batteries.
- The critical influence of multi-step reaction kinetics, distinct voltage plateaus, and dominant charge carriers on solar cell coupling strategies is decoupled, with emphasis on maximum power point matching and intermittent charging adaptability.
- A prioritized, roadmap-oriented perspective is provided, delineating the short- to long-term scientific and engineering milestones needed to translate multi-electron zinc chemistry into commercially viable self-powered technologies.

## Graphical abstract

**Figure:**
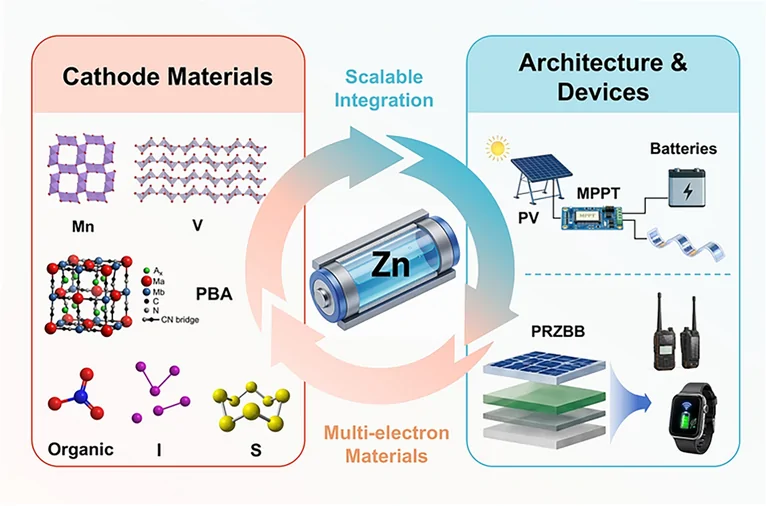

## Introduction

**Fig. 1:**
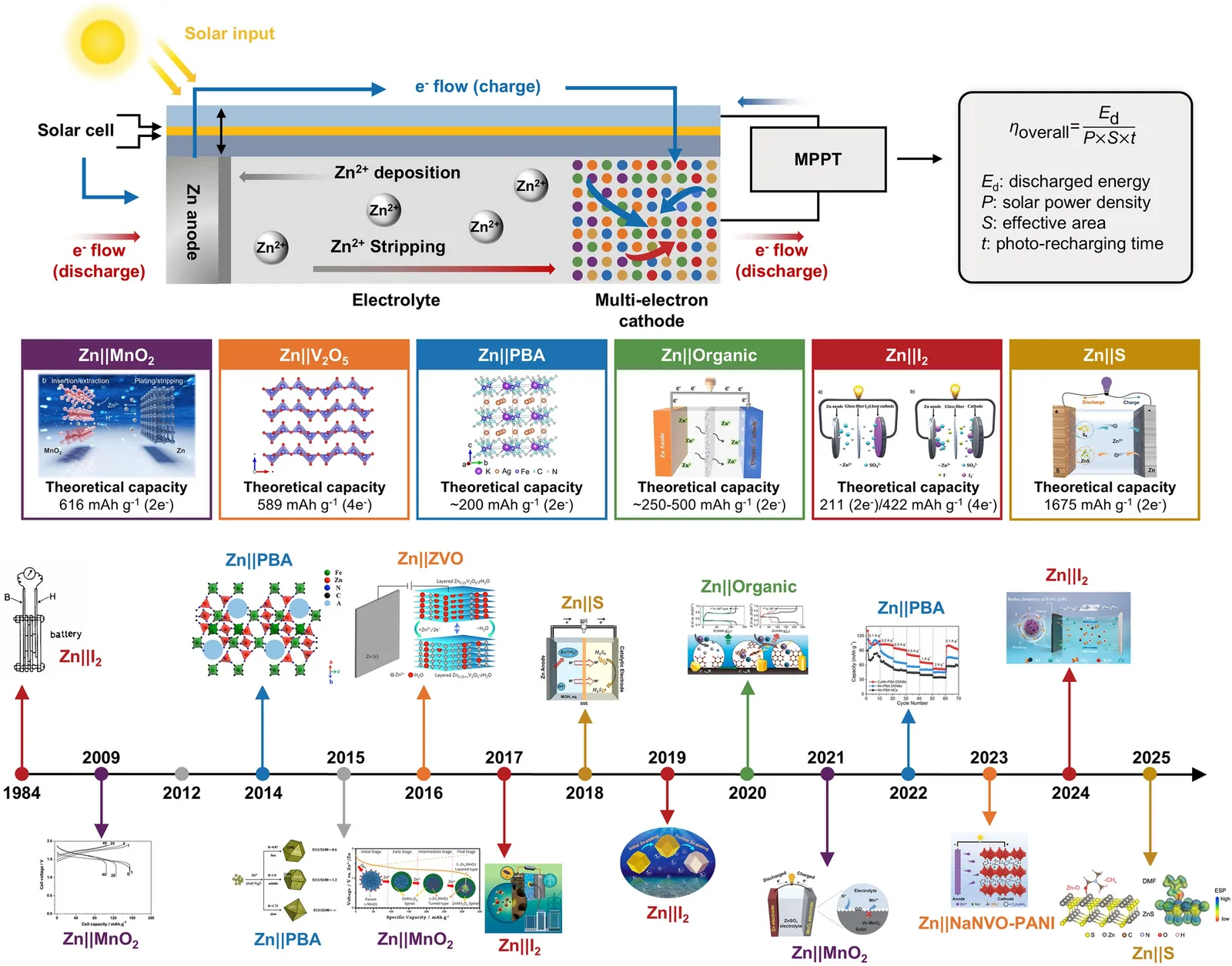
Integrated design, working principles, and evolution of Zn-based batteries via multi-electron transfer [6–26]. Copyright 2023 by the authors, published by Springer Nature, Copyright 2025 Wiley–VCH, Copyright 2025 Wiley–VCH, Copyright 2020 Wiley–VCH, Copyright 2023 Wiley–VCH, Copyright 2023 Wiley–VCH, Copyright 1984 Elsevier, Copyright 2009 Elsevier, B.V., Copyright 2014 Wiley–VCH, Copyright 2015 American Chemical Society, Copyright 2015 Scientific Reports, Copyright 2016 Macmillan Publishers Limited, part of Springer Nature, Copyright 2017 American Chemical Society, Copyright 2018 American Chemical Society, Copyright 2019 Elsevier Inc, Copyright 2020 American Chemical Society, Copyright 2021 by the authors, published by Springer Nature, Copyright 2022 Wiley–VCH, Copyright 2023 Wiley–VCH, Copyright 2024 Wiley–VCH, Copyright 2025 by the authors, published by Springer Nature

With the rapid proliferation of portable electronics and distributed edge devices in the era of the Internet of Things (IoT) networks, the demand for autonomous and self-powered energy systems has become increasingly urgent [1, 2]. Among emerging solutions, photo-rechargeable batteries, which are capable of harvesting and storing solar energy in situ, attracted significant attention due to their potential for uninterrupted, off-grid operation [3]. Among a wide range of candidates, photo-rechargeable zinc-based batteries (PRZBBs) stand out due to their inherent advantages, including high theoretical capacities, natural abundance, environmental benignity, and excellent safety in aqueous electrolytes [4]. Zinc possesses a high hydrogen evolution overpotential and a moderate redox potential ( − 0.76 V vs. SHE), making it relatively stable in aqueous electrolytes. Coupled with its natural abundance and high theoretical capacity (820 mAh g^−1^), zinc serves as an ideal platform for multi-electron transfer studies. In contrast, although aluminum and magnesium offer higher theoretical volumetric capacities, they are prone to forming dense passivation layers and suffer from severe hydrogen evolution side reactions, resulting in sluggish multi-electron reaction kinetics and poor reversibility. Meanwhile, iron-based batteries are primarily constrained by lower operating voltage plateaus and a susceptibility to irreversible phase transitions. Consequently, zinc-based batteries exhibit a unique synergy of advantages within aqueous multi-electron transfer systems, fundamentally explaining their emergence as a research hotspot for integrated photo-rechargeable, self-powered technologies. When coupled with high-efficiency solar cells, these systems can form compact and fully integrated power units that efficiently capture and store solar energy with minimal energy conversion losses (Fig. 1) [5].

A critical parameter for evaluating the performance of PRZBBs is the overall energy conversion efficiency (*η*_overall_), which is calculated as [27]:

1 $${\eta}_{\mathrm{o}\mathrm{v}\mathrm{e}\mathrm{r}\mathrm{a}\mathrm{l}\mathrm{l}}=\frac{{E}_{d}}{P\times S\times t}$$

where *E*_d_ is the discharge energy of the Zn-based battery, *P* is the incident solar power, *S* is the effective area of the solar cell, and *t* is the photo-recharging time. According to this equation, one of the most effective strategies to improve *η*_overall_ is to enhance the discharge energy of Zn-based batteries, which is further determined by the discharge capacity of two electrodes and the discharge voltage. Consequently, this review posits that advancing the cathode chemistry of Zn-based batteries toward multi-electron transfer reactions is a crucial strategy for developing high-performance PRZBBs. This approach directly targets the key parameters in Eq. (1). First, multi-electron transfer significantly boosts the specific capacity (C = nF/3.6M), where F, M, and 3.6 represent the Faraday constant (96,485 C mol^−1^), molecular weight, and the ratio between Coulombs and mAh, thereby directly increasing *E*_*d*_ [28]. Second, such reactions often involve multiple distinct voltage plateaus, which can collectively create a wide and high operating voltage window. Optimizing these plateaus, particularly the highest ones, is vital for aligning with the maximum power point (*V*_mpp_) of the coupled solar cell, minimizing energy losses during the photo-charging process. Finally, achieving efficient multi-electron redox requires rapid kinetics, which facilitates faster photo-charging rates, shortens t in Eq. (1), and enhances the overall energy harvesting efficiency of the integrated system.

However, fabricating aqueous Zn-based batteries with such high-energy–density cathodes faces challenges, including parasitic reactions like hydrogen or oxygen evolution that limit the operational voltage window. Therefore, substantial research focuses on developing stable, high-capacity cathode materials. To this end, a wide variety of redox-active materials have been explored to enable multi-electron transfer pathways. Manganese-based compounds (e.g., MnO_2_) facilitate Zn^2+^ or H^+^ intercalation/extraction via Mn^4+^/Mn^3+^/Mn^2+^ transitions [29]. Vanadium-based compounds offer rich redox chemistry with multiple V^5+^/V^4+^/V^3+^ states, delivering reversible electrochemical behavior and tunable voltage profiles [30]. Prussian blue analogues (PBAs) provide open-framework structures with multiple redox centers, promoting fast Zn^2+^ transport and high cycling stability [31]. Organic molecules, such as quinones and azo compounds, utilize π-conjugated frameworks and delocalized electron systems to stabilize multi-electron redox processes [32]. Similarly, halogens (e.g., I_2_/I^−^) and elemental sulfur exhibit exceptionally high theoretical capacities due to their ability to undergo 2–8 electron redox reactions per molecule or atom [33, 34]. These advanced redox systems, which range from inorganic compounds, organic compounds, and elements (halogens and sulfur), are particularly promising for constructing next-generation PRZBBs when integrated with solar cells. These devices not only enable high-energy density and rapid solar charging, but also pave the way for sustainable, lightweight, and flexible power systems ideally well-suited for autonomous electronics. Moreover, the aqueous-based design and mechanical adaptability of Zn-based batteries align well with the structural and environmental requirements of flexible and wearable devices.

While recent years have witnessed a surge in review articles covering aqueous zinc-based batteries and photo rechargeable energy systems, a distinct gap still exists in the current literature. Most existing reviews primarily focus either on general cathode material optimization, often limited to single-electron intercalation mechanisms, or on the macroscopic device engineering of photobatteries without sufficiently addressing the underlying cathode chemistry. In contrast, this review uniquely adopts multi-electron transfer as the central organizing principle to bridge fundamental electrochemistry with macroscopic device integration. By systematically decoupling the multistep redox pathways across diverse material classes, including inorganic compounds, organic compounds, halogens, and sulfur, this manuscript establishes a comprehensive framework for utilizing high-valency redox chemistry to overcome the specific capacity and voltage matching limitations inherent in next-generation photo-rechargeable zinc-based batteries (PRZBBs). A detailed comparison highlighting the novelty of this review relative to previous literature is summarized in Table 1.

**Table 1:** Comparison of the scope and novelty of this review relative to existing literature

Review category	Typical focus and scope in existing literature	The unique focus of this work
General Zn-Based Batteries	Broad overview of anode protection, electrolyte engineering, or specific intercalation cathodes (e.g., just V-based or Mn-based)	Systematic synthesis of multi-electron mechanisms across a highly diverse material spectrum (Inorganics, Organics, Halogens, S) in a single framework
Organic/Halogen Batteries	Deep dive into specific organic functional groups or polyiodide shuttle suppression	Evaluates these materials specifically through the lens of amplifying electron transfer numbers to boost overall energy density
Integrated Photobatteries (PRZBBs)	Heavy emphasis on macroscopic device engineering (flexible substrates, circuit series/parallel design) and solar cell types	Bridges fundamental cathode multi-electron kinetics and voltage plateau modulation with the specific requirements of solar charging (e.g., V_mpp_ matching)

In this review, we first systematically summarize the fundamental redox mechanisms and material design strategies for multi-electron Zn-based battery chemistries. Building upon these highly reversible and energy-dense electrochemical foundations, we subsequently explore their critical integration with solar cells to construct fully self-powered energy storage systems. Representative cathode materials, including inorganic compounds (manganese oxides, vanadium oxides, and PBAs), organic compounds, and elements (halogen and sulfur), are systematically reviewed in terms of their redox mechanisms, energy storage behavior, and photo-rechargeable performance. We also highlight the major challenges and future directions for realizing efficient, durable, and scalable PRZBBs toward real-world applications.

## Theoretical Basis of Multi-Electron Transfer Mechanisms

**Fig. 2:**
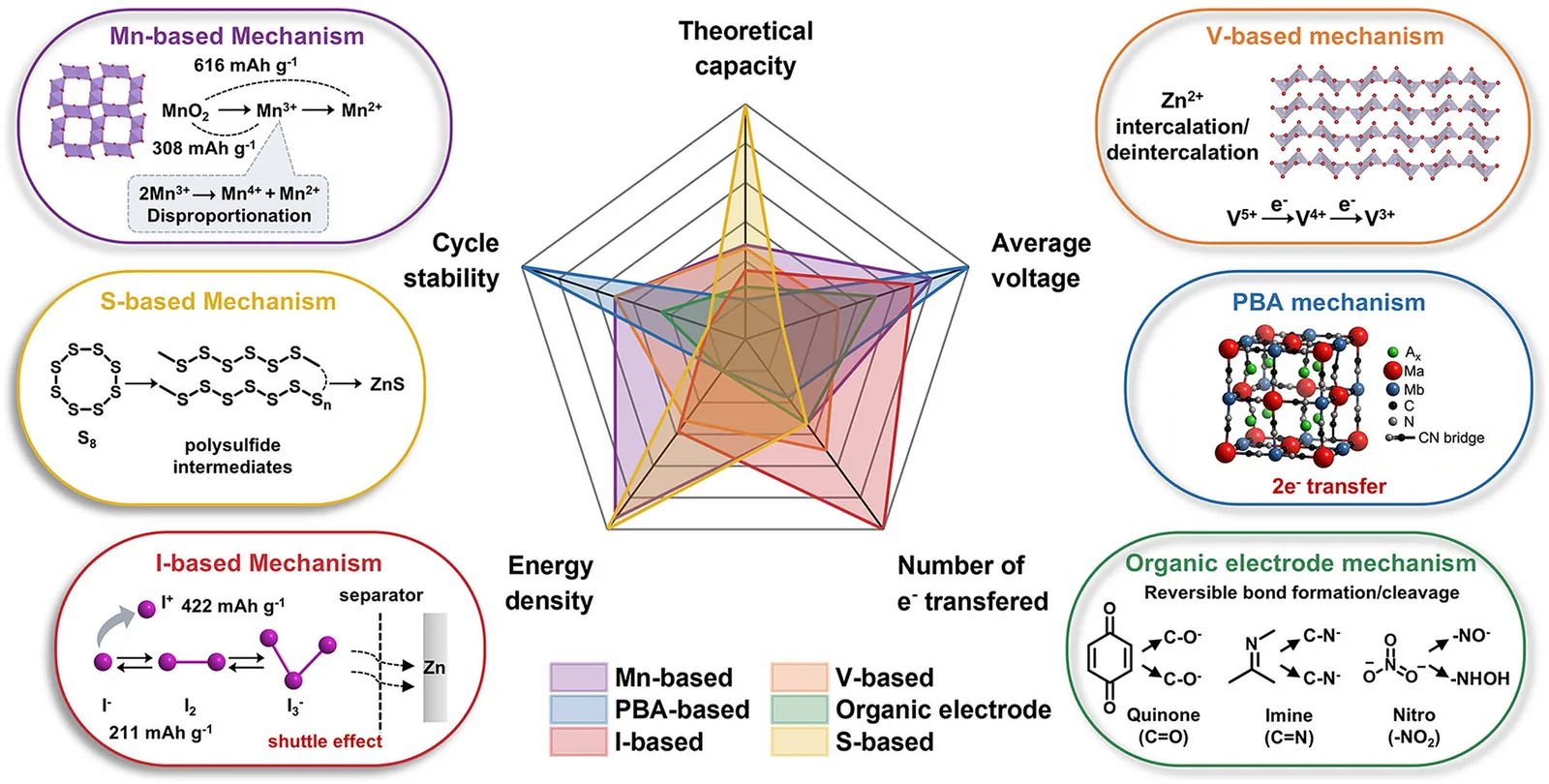
Performance landscape and charge-storage mechanisms of Zn-based batteries via multi-electron transfer [42, 43]. Copyright 2024 Elsevier, B.V., Copyright 2024 Elsevier, B.V

A comprehensive understanding of the charge storage mechanisms is essential for the rational design of advanced electrode materials. In Zn-based batteries, multi-electron transfer reactions, which are defined as the involvement of multiple electrons per redox center through cathode redox modulation, hold significant promise [35]. These reactions involve the transformation of cathode materials into different chemical states or valence configurations during charge/discharge, effectively enabling energy storage through reversible structural or compositional changes (Fig. 2) [36]. Theoretically, the specific capacity of an electrode is directly proportional to the number of electrons transferred during the redox reaction [37]. Compared to single-electron processes, multi-electron redox reactions occurring at higher redox potentials can simultaneously deliver enhanced energy in Zn-based batteries (Table 2) [38, 39]. However, it is crucial to emphasize that an experimentally observed high specific capacity does not inherently prove the occurrence of multi-electron transfer. Elevated capacities can frequently arise from confounding factors such as pseudocapacitive contributions, parasitic side reactions or the dynamic dissolution/redeposition of inactive species. Therefore, while high capacity is a macroscopic prerequisite, definitively confirming a multi-electron mechanism strictly requires dynamic microstructural and compositional corroboration. Nonetheless, most current cathodes suffer from limited theoretical capacities and poor intrinsic conductivity [40]. Therefore, the exploration of multi-electron transfer cathode materials, alongside in-depth mechanistic investigations, is vital to unlocking the full potential of Zn-based batteries. Notably, achieving efficient multi-electron transfer often requires specific activation strategies to overcome kinetic or thermodynamic barriers associated with high-valency redox chemistry [41].

**Table 2:** Performance comparison of zinc-based batteries with different cathode material systems

Indicator	Mn-based (2e^−^)	V-based	PBAs	Organic	I-based (4e^−^)	S-based
Theoretical specific capacity (mAh g^−1^)	616 (Mn^4+^/Mn^2+^)	589 (V_2_O_5_, V^5+^/V^3+^)	85–170 (metal-dependent)	200–600 (group-dependent)	422(I^−^/I^+^)	1675 (S → ZnS)
Typical practical capacity (mAh g^−1^)	400–580(optimized systems)	300–550	120–180	150–400	350–420	800–1400
Average discharge voltage (V vs. Zn/Zn^2+^)	~ 1.8	0.8–1.5 (multi-platform)	1.0–1.7	0.5–1.6 (type-dependent)	~ 1.45	~ 0.6–0.8
Number of transferred electrons	1–2 e^−^	2–6 e^−1^	1–4 e^−^	2–24 e^−^	2–4 e^−^ (12 e^−^)	16 e^−^ (per S_8_)

### Manganese Oxides-Based Multi-Electron Transfer Mechanism

Manganese dioxides (MnO_2_) typically operate on the single-electron Mn^4+^/Mn^3+^ redox couple, delivering a moderate theoretical capacity of 308 mAh g^−1^ [44]. In contrast, the two-electron Mn^4+^/Mn^2+^ redox reaction offers a significantly higher theoretical capacity of 616 mAh g^−1^, effectively doubling that of the single-electron process [45]. However, the electrochemical pathway from Mn^4+^ to Mn^2+^ is notably complex in aqueous electrolytes. This reaction pathway initiates with the oxidation of Mn^2+^ to Mn^3+^ intermediates on the electrode surface. Subsequently, these Mn^3+^ intermediates undergo disproportionation, yield both Mn^4+^ and Mn^2+^, experience hydrolysis, and ultimately regenerate MnO_2_. In neutral electrolytes, MnOOH often forms as an intermediate and can reversibly transform into Mn^3+^ [46, 47]. The disproportionation reaction of Mn^3+^ (during the discharge process): $2{Mn}^{3+}\to {Mn}^{4+}+{Mn}^{2+}$, the complete reaction contextualized within the electrolyte environment (a common representation involving MnOOH as an intermediate): $2{\mathrm{MnOOH}} + 2{\mathrm{H}}^{ + } \to {\mathrm{MnO}}_{2} + {\mathrm{Mn}}^{{2 + }} + 2{\mathrm{H}}_{2} {\mathrm{O}}$. In acidic or near-neutral electrolytes, Mn^3+^ is highly prone to disproportionation, leading to the dissolution of a portion of the active material into the electrolyte in the form of Mn^2+^. By optimizing the electrolyte composition (e.g., through the pre-addition of Mn^2+^ salts) or carefully regulating the pH, this irreversible disproportionation can be effectively mitigated. Such strategies also facilitate the re-oxidation of Mn^2+^ back to MnO_2_ during the charging process, thereby significantly enhancing the cycling stability of the two-electron reaction. During discharge, MnO_2_ reacts with H^+^ to form Mn^2+^ (${\mathrm{MnO}}_{2} + 4{\mathrm{H}}^{ + } + 2{\mathrm{e}}^{ - } \to {\mathrm{Mn}}^{{2 + }} + 2{\mathrm{H}}_{2} {\mathrm{O}}$, *E*^Θ^ = 1.228 V vs. SHE). The kinetics of Mn^2+^ dissolution and redeposition are intimately tied to local pH fluctuations. Proton consumption during discharge raises the interfacial pH, stabilizing Mn^3+^ initially but promoting Mn^2+^ solubility once disproportionation occurs. During charge, proton release creates a local acidic front that drives Mn^2+^ redeposition as MnO_2_. However, Mn^2+^ that have diffused beyond this acidic zone into the bulk electrolyte escape redeposition, causing irreversible capacity loss. Furthermore, the high operating potential of the Mn^4+^ to Mn^2+^ conversion exacerbates the electrochemical and thermal instability in Mn-based materials. Concurrently, the disproportionation of Mn^3+^ intermediates during cycling can lead to the formation of irreversible phases, further compromising the structural reversibility and cycling performance of the cathode [48].

### Vanadium Oxides-Based Multi-Electron Transfer Mechanism

Vanadium oxides (VO_x_) possess diverse valence states (ranging from + 2 to + 5) and can undergo multi-electron redox reactions, thereby offering a high theoretical capacity (> 300 mAh g^−1^) and attracting significant attention [49]. In zinc-based batteries, the two-electron transfer process of V^5+^ → V^4+^ → V^3+^ is the most prevalent. The corresponding potential ranges for these transitions are typically 0.8–1.0 V and 0.5–0.7 V (vs. Zn^2+^/Zn), respectively. Each transition contributes a theoretical capacity of approximately 147 mAh g^−1^ (yielding a cumulative capacity of 294 mAh g^−1^). Although the further reduction of V^3+^ to V^2+^ (at potentials < 0.3 V) could theoretically provide an additional capacity of 147 mAh g^−1^, this step results in a significantly lower average discharge voltage and exhibits poor reversibility, thereby limiting its practical application. It is worth noting that for different vanadium-based materials (e.g., LiV_3_O_8_, NH_4_V_4_O_10_), these redox potentials may exhibit slight shifts (approximately 0.1 V) due to variations in their intrinsic crystal structures. Among them, V_2_O_5_ and its derivatives are the most representative cathodes in Zn-based batteries, offering a high theoretical capacity of 589 mAh g^−1^ based on the V^3+^/V^5+^ redox couple [50]. During discharge, V^5+^ ions in the V_2_O_5_ lattice can be gradually reduced by gaining electrons, transitioning from V^5+^ to V^4+^ and eventually to V^3+^ [51]. The exact number of electron transfer depends on the specific material and battery design, with the V^5+^/V^4+^ conversion typically dominating the early-stage discharge reaction. Upon charging, the reverse reaction occurs as vanadium ions in lower oxidation states lose electrons through oxidation. This multi-electron conversion is generally accompanied by the reversible (de)insertion of Zn^2+^ ions in the VO_x_ lattice to maintain charge balance. The multi-electron reaction mechanism of VO_x_ cathodes is a complex process, which is influenced by various factors such as the material phase, particle size, morphology, and electrolyte compositions [52]. Optimizing these factors is crucial for achieving high-performance V-based cathodes in Zn-based batteries.

### PBAs-Based Multi-Electron Transfer Mechanism

Prussian blue analogues (PBAs) have gained considerable attention as promising cathode materials for Zn-based batteries due to their open three-dimensional framework, high theoretical capacity, easy synthesis, and environmental compatibility [108]. Their characteristic composition follows the general formula of A_x_M_a_[M_b_(CN)_6_]_y_, where A denotes alkali metals (such as Na^+^, K^+^, etc*.*), and M_a_/M_b_ represents transition metals. PBAs typically adopt a face-centered cubic crystal structure with large and open interstitial voids, thereby providing abundant and tunable redox-active sites [109, 110]. This structural versatility establishes PBAs as an appealing platform for enabling multi-electron transfer reactions, a critical factor in achieving high capacity. The multi-electron chemistry in these materials primarily stems from the reversible redox activity of transition metal ions (e.g., Fe^2+^/^3+^, Mn^2+^/^3+^/^4+^, Cu^+^/^2+^) located at both high-spin (M_a_) and low-spin (M_b_) sites [111, 112]. To fully exploit the potential of PBAs, compositional engineering through the selective incorporation of transition metal ions (such as Mn, Fe, and Cu) can introduce multiple redox couples per formula, directly increasing the number of electrons transferred. Besides, the elemental doping in PBAs can alter its electronic structures and electrochemical properties, further promoting multi-electron transfer. Furthermore, manipulating the synthesis conditions of PBAs (e.g., pH, temperature, and time) can control the degree of crystalline ordering, thus influencing electronic conductivity and facilitating multi-electron transfer reactions [113]. These factors collectively govern the kinetics and reversibility of the multi-electron processes. Additionally, the development of composite materials or nanostructures can facilitate the ion/electron transport pathway and enhance the electrochemical activity of PBAs, thereby stabilizing the multi-electron transfer reaction during the cycling. Through these synergistic strategies, PBAs can effectively overcome their inherent limitations like moderate electronic conductivity and structural instability, fully unlocking their multi-electron redox potential toward the applications in Zn-based batteries [114].

### Organic Materials-Based Multi-Electron Transfer Mechanism

The multi‑electron transfer capability of organic materials originates from the sequential redox reactions occurring at distinct active sites within the molecule at specific potentials. Based on their charge storage mechanisms, organic electrodes are primarily classified into n‑type, p‑type, and bipolar types [115]. In n‑type electrodes, carbonyl (C=O) groups enable two‑electron transfer through double‑bond cleavage and yield C–O^−^ bonds. To enhance the capacity, compounds containing multiple carbonyl groups, such as quinones and ketones, are commonly employed [116, 117]. Similarly, imine (C=N) groups also participate via analogous bond cleavage (forming C–N^−^). Polyamide compounds like phenazine can receive 2 to 6 electrons. Most n‑type materials integrate both C=O and C=N groups to cooperatively increase the number of electron transfer [118, 119]. The nitro group (-NO_2_), a strong electron‑withdrawing moiety, exhibits high redox activity and can undergo a multi‑step reduction process. It is first reduced to a nitroso (-NO, 2e^−^ transfer), then to hydroxylamine (-NHOH, 2e^−^ transfer), and potentially further to amine (-NH_2_, 2e^−^ transfer). Consequently, each nitro group can theoretically transfer 4 to 6 electrons. Introducing multiple nitro groups into a single molecule, as in nitroaromatic polymers or conjugated frameworks, enables synergistic multi-electron transfer, significantly boosting the specific capacity [120, 121].

Among p-type materials, polyaniline (PANI) exemplifies a system whose conductivity stems from its conjugated π-electron system and reversible oxidation states (e.g., leucoemeraldine, emeraldine, pernigraniline) [66]. Under acidic or oxidizing conditions, the imine (-NH-) in the PANI backbone is oxidized, generating positively charged polarons or bipolarons, with holes acting as the primary charge carriers to form conductive pathways [122, 123]. Charging follows the reaction pathway of PANI → PANI^n+^  + ne^−^, accompanied by the insertion of anions (e.g., SO_4_^2−^) to maintain charge balance, with discharge being the reverse process. Sulfur-containing p-type compounds (e.g., polythiophene, thionated conjugated polymers) leverage the redox activity of sulfur within the conjugated structure along with π-electron delocalization to store multiple electrons. Sulfur atoms possess multiple oxidation states and can undergo multi-step electron transfer during cycling, enabling high capacity [72]. During reduction, disulfide (–S–S–) bonds cleave to form thiols (–SH) with concomitant multi-electron transfer, offering a substantially high capacity.

Bipolar electrodes incorporate both n-type and p-type functional groups, combining the advantages of each. This dual-mode design enables complementary reduction and oxidation processes to occur sequentially within a single material, effectively expanding the accessible redox reactions per formula unit and thereby substantially increasing the theoretical specific capacity. More importantly, such integration broadens the operational voltage window by utilizing both high-potential p-type and low-potential n-type reactions, which contributes to a higher overall energy density. The internal electronic coupling between the different redox centers can also enhance charge transfer kinetics and improve structural stability during cycling, leading to better rate capability and longer lifespan compared to electrodes based on a single redox type.

The multi-electron transfer in organic electrodes is essentially the result of cooperative redox reactions among multiple active sites within the molecule. Optimizing their performance requires balancing the number of functional groups, structural stability, and electronic conductivity [124]. In the future, integrating computational materials science with nanotechnology is expected to overcome existing bottlenecks and advance the practical implementation of organic batteries [125].

### Iodine-Based Multi-Electron Transfer Mechanism

The halogens with multiple valence states (− 1 to + 7) are compelling cathode materials for high-energy Zn-halogen batteries. Moreover, the halogens exhibit fast reaction kinetics compared to metal oxide cathodes, due to their excellent solubility in electrolytes [126–128]. Among them, Zn||I_2_ batteries show significant potential due to the elemental abundance, high capacity, rich redox states, and elevated redox potential of iodine [129]. The conventional redox mechanism is the two-electron I^−^/I^0^ reaction (I_2_ + 2e^−^  ⇌ 2I^−^), which delivers a theoretical capacity of 211 mAh g^−1^ at a moderate potential of ~ 0.54 V versus the standard hydrogen electrode (SHE) [130]. However, this process intrinsically limits the achievable capacity and energy density.

To overcome this constraint, multi-electron pathways involving higher oxidation states of iodine, such as I^+^ and I^5+^, have been explored [131]. The four-electron I^−^/I^+^ redox couple can double the capacity to 422 mAh g^−1^ and raise the reaction potential to ~ 1.45 V vs. SHE [132, 133]. Nevertheless, the oxidation of I_2_ to I^+^ (e.g., in the form of hypoiodous acid) faces significant challenges. The symmetric structure of the I_2_ molecule impedes efficient interaction with water, slowing down the reaction kinetics [134]. More critically, the required oxidation potential overlaps with the OER in aqueous electrolytes [135]. Furthermore, I^+^ species are highly susceptible to hydrolysis, resulting in poor reversibility of the I^0^/I^+^ redox chemistry [136].

Beyond the I^−^/I^+^ redox reaction, an even more ambitious route is the utilization of the 12-electron IO_3_^−^/I^−^ couple, which offers a remarkable theoretical capacity of 1266 mAh g^−1^ [137]. This process entails a complex mechanism: the oxidation pathway involves disproportionation reactions (2I^−^  → I_2_ → HIO → IO_3_^−^), while the reduction pathway, though autocatalytic in part (2IO_3_^−^  + 12H^+^  + 12e^−^  → 2I^−^  + 6H_2_O), is initiated by a kinetically sluggish step. The substantial energy barriers make highly reversible cycling exceedingly difficult [138, 139].

Besides the inherent complexity of these multi-electron reactions, iodine-based cathodes universally suffer from the shuttle effect of soluble polyiodide intermediates (I_3_^−^/I_5_^−^), which leads to rapid self-discharge and low Coulombic efficiency. This issue becomes increasingly severe in multi-electron processes where a wider variety of mobile species are generated. Consequently, the demonstration of high-energy and long-life Zn||I_2_ batteries necessitates the development of targeted modification strategies. These strategies must simultaneously catalyze the complex multi-step redox conversions, suppress competing side reactions (such as OER and hydrolysis), and effectively immobilize the active iodine species to fully harness the potential of multi-electron iodine redox chemistry.

### S-Based Multi-Electron Transfer Mechanism

Sulfur is another representative multi-electron cathode in Zn-based batteries, which offers a high theoretical capacity of 1675 mAh g^−1^ via a series of two-electron S^0^/S^2−^ reaction. Furthermore, sulfur holds additional advantages in material sourcing and manufacturing costs, therefore facilitating the development of cost-effective battery systems [140]. The operation of Zn||S batteries relies primarily on the overall redox reaction of 16Zn^2+^  + S_8_ + 32e^−^  → 8ZnS [141]. During discharge, the solid sulfur (S_8_) is activated and gradually converts into polysulfide intermediates (S_n_^2−^) with the insertion of Zn^2+^ cations, and ultimately precipitates as the final discharge product, ZnS. This sequence is reversed during charging.

Despite the high-energy and low-cost merits of Zn||S batteries, the multi-electron conversion in Zn||S batteries is fraught with fundamental challenges. During charge/discharge cycles, the polysulfide intermediates react with Zn^2+^ to form ZnS, which results in slow solid–solid S/ZnS reaction kinetics, thereby resulting in high activation energy barriers. This manifests as large voltage hysteresis, poor rate capability, and low practical sulfur utilization. Additionally, ZnS can undergo oxidization side reactions, which reacts with water to generate irreversible by‑products (2ZnS + 4H_2_O − 10e^−^  → 8H^+^  + S + 2Zn^2+^  + SO_4_^2−^) [99], causing active material loss at the cathode and impairing cycling stability of the Zn||S batteries.

## Material Design and Electrode Structure Optimization

Before detailing the specific material engineering strategies, it is imperative to establish a rigorous benchmarking framework for evaluating electrochemical performance. In the current literature, performance metrics such as specific capacity and energy density are frequently reported under highly divergent testing conditions. Most critically, specific energy values are conventionally calculated based exclusively on the active mass of the cathode, which drastically overestimates the practical energy density of the full device. However, for the future development of next-generation photo-rechargeable zinc-based batteries (PRZBBs), evaluating true device-level overall energy conversion efficiency necessitates stricter standardizations. Moving forward, the community must rigorously control and report practical device design parameters, particularly the Negative-to-Positive (N/P) capacity ratio, the depth of discharge (DOD) of the zinc anode, and the electrolyte-to-capacity (E/C) ratio, to bridge the gap between fundamental cathode chemistry and scalable, self-powered applications.

### Manganese Oxides-Based Multi-Electron Transfer Zn-Based Batteries

In manganese oxides-based Zn-based batteries, lowering the electrolyte pH and increasing the charging voltage have been demonstrated as effective strategies to activate the two-electron Mn^4+^/Mn^2+^ redox couple [142, 143]. However, such conditions often induce severe material corrosion and pose significant safety risks, thereby limiting their applicability in large-scale energy storage. Consequently, there is an urgent need to develop manganese oxides-based Zn-based batteries that can undergo multi-electron transfer reactions under milder and more practical conditions [42].

#### Electrolyte Engineering

By optimizing electrolyte properties, particularly pH values and electrolyte compositions, the multi-electron transfer of Mn^4+^/Mn^2+^ reactions can be promoted, significantly enhancing the specific capacity of the MnO_2_ cathode. Additionally, the incorporation of electrolyte actives, such as acetate salts or organic molecules, can reduce side reactions and enhance the cycling stability of MnO_2_ [144].

**Fig. 3:**
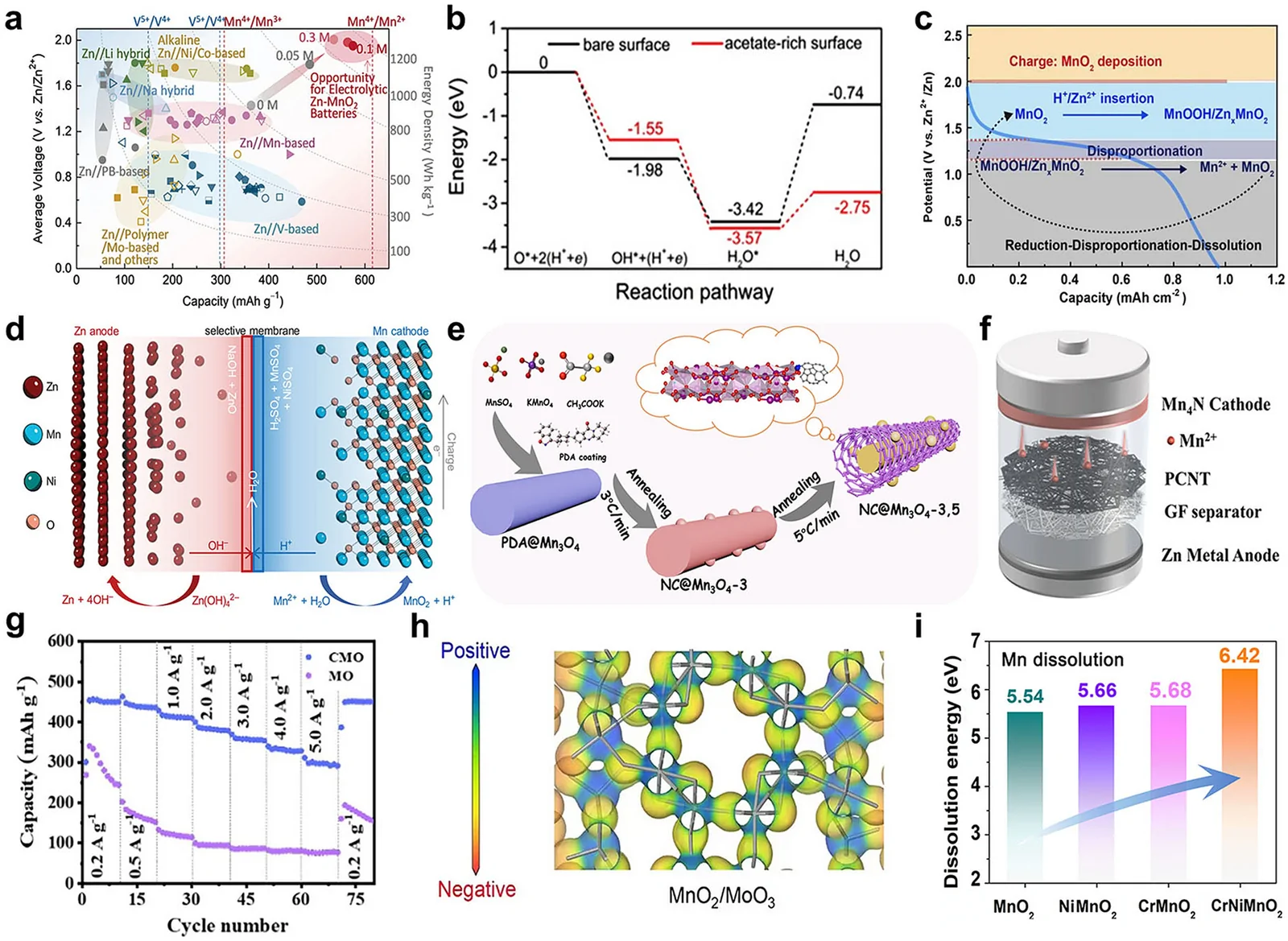
**a** Specific capacities and average discharge voltages of other Zn battery systems. Reproduced with permission [143]. Copyright 2019, Wiley–VCH. **b** Energy diagram of the dissolution reaction on MnO_2_. Reproduced with permission [145]. Copyright 2020, Wiley–VCH. **c** Schematic illustration of the discharge mechanism for the hybrid battery in a mild electrolyte. Reproduced with permission [146]. Copyright 2022, Elsevier. **d** Schematic depiction of HAB in hybrid electrolytes during charge/discharge cycles. Reproduced with permission [142]. Copyright 2020, Wiley–VCH. **e** Diagram illustrating the synthetic procedure for NC@Mn_3_O_4_ core–shell composites. Reproduced with permission [148]. Copyright 2024, Elsevier. **f** Schematic representation of the Zn||Mn_4_N-PCNT battery configuration. Reproduced with permission [149]. Copyright 2024, Wiley–VCH.** g** Rate performance of CMO and MO at various current densities. Reproduced with permission [150]. Copyright 2022, Elsevier. **h** Calculated electrostatic potential distributions in MnO_2_ and MnO_2_/MoO_3_. Reproduced with permission [151]. Copyright 2023, American Chemical Society. **i** Dissolution energy of the Mn dissolution. Reproduced with permission [152]. Copyright 2024, Elsevier

A breakthrough was achieved by introducing a high-voltage Zn||MnO_2_ battery in the mixed electrolyte of ZnSO_4_ + MnSO_4_ + 0.1 M H_2_SO_4_, where the acidic environment (pH ≈ 1.15) facilitates a Mn^4+^/Mn^2+^ reaction aided by vacancy formation in MnO_2_, leading to a record output voltage of 1.95 V, a capacity of ~ 570 mAh g^−1^, and an unprecedented specific energy of ~ 409 Wh kg^−1^ (Fig. 3a) [143]. Note that the specific energy in this review is based on the active mass of cathode material only. Alternatively, using acetate anions in a near-neutral electrolyte (pH ≈ 6) can modify the MnO_2_ surface to lower the dissolution energy barrier, enabling the Mn^4+^/Mn^2+^ redox while simultaneously forming a protective layer on the Zn anode to suppress dendrites. This approach yielded a high capacity of 556 mAh g^−1^ and exceptional stability over 4000 cycles (Fig. 3b) [145]. Innovative electrolyte design can also tackle inherent challenges. Liu et al*.* utilized the typically detrimental disproportionation of Mn^3+^ to drive an efficient Mn^2+^/Mn^4+^ conversion in a mild electrolyte **(**pH = 4.3, Fig. 3c) [146]. The discharge process involves sequential H^+^/Zn^2+^ insertion, Mn^3+^ disproportionation, and dissolution, delivering a high capacity of 550 mAh g^−1^ over 5000 cycles. Similarly, a nanomicellar electrolyte with methylurea (Mu) was designed to encapsulate Zn^2+^/Mn^2+^ ions, controlling their release to enable uniform deposition and suppress water activity. This system enhanced the 2e^−^ transfer process, achieving ~ 100% capacity retention after 800 cycles and a specific energy of 800 Wh kg^−1^ [147]. Advanced concepts like a two-compartment cell with an acidic Mn^2+^-containing catholyte and an alkaline Zn^2+^-containing anolyte expand the water stability window, effectively preventing water decomposition reactions (Fig. 3d) [142]. Meanwhile, adding Ni^2+^ to the catholyte further improves reaction kinetics. Density functional theory (DFT) calculations reveal that Ni^2+^ doping increases the activity of the O 2*p* electron states, promote charge transfers and thus lower the overpotential for electrolytic kinetics. As a result, the MnO_2_ cathode achieves a high voltage of 2.44 V, an impressive specific energy of ~ 650 Wh kg^−1^, a power density of 19 kW kg^−1^, and excellent cycling stability.

To sustain the 2e^−^ reaction and stabilize the Zn interface, another strategy is to employ a proton-redistributing hydrogel electrolyte, where distinct acidic (cathode), alkaline (intermediate), and neutral (anode) zones are present. This method achieves a specific capacity of 516 mAh g^−1^ at 0.05 A g^−1^ and remarkable cycling stability [153]. Compared to the conventional aqueous electrolytes (2 M ZnSO_4_ + 0.2 M MnSO_4_), such cross-linked hydrogels can broaden the electrolyte stability window and suppress Mn^2+^ shuttle, leading to a specific energy of 858 Wh kg^−1^ (α-MnO_2_) and stability over 7000 cycles [154].

An optimized acetate-based electrolyte incorporates a controlled Cr^2+^/Cr^3+^ redox reaction to enhance the potential and lifespan [155]. This electrolyte features an acidic layer adjacent to the cathode to support a 2e^−^ redox reaction, a neutral layer next to the Zn anode to mitigate dendrite formation, and a mildly alkaline intermediate layer to stabilize proton distribution and suppress water-splitting side reactions. The combination of efficient Mn^2+^/Mn^4+^ redox reactions and effective anode protection is essential for superior battery performance. By integrating a pre-cycling process and ultrasonic treatment, this optimized system triples the lifespan, achieving an impressive accumulated capacity of 10 Ah cm^−2^ and a power density exceeding 75 mW cm^−2^.

Electrolyte regulation is an effective strategy to promote multi-electron transfer in Zn||MnO_2_ batteries [156]. However, optimizing the electrolyte often requires the use of additional additives, such as metal salts or organic compounds, which may reduce the ionic conductivity of the electrolyte. Furthermore, complex electrolyte formulations may complicate the manufacturing process. Future research should focus on improving the stability, conductivity, and cost-effectiveness of electrolytes to facilitate the widespread practical application of Zn-based batteries.

#### Cathode Materials Design

The cathode design and doping strategy is promising to achieve multi-electron transfer in MnO_2_ cathodes and enhance its electrochemical performance. It is worth mentioning that doping can regulate the structural and electrochemical properties of the cathode material, effectively suppress the manganese dissolution and the shuttle effect while simultaneously increase the redox-active sites. These improvements result in higher manganese utilization, enhanced rate capability, and extended cycle life [157].

Structural engineering focuses on the construction of robust and conductive frameworks. To address the Jahn–Teller distortion, manganese oxides with a pre-embedded high Mn^2+^ content were electrodeposited onto the carbon cloth. This design, coupled with generated oxygen vacancies, facilitated efficient ion diffusion and mitigated side reactions, yielding a high areal capacity of 4.16 mAh cm^−2^ and stability over 4000 cycles [158]. A core–shell structured N-doped carbon@Mn_3_O_4_ (NC@Mn_3_O_4_) cathode was fabricated via a multi-step annealing process (Fig. 3e) [148]. The core–shell architecture buffers the volume change, while the N-doping elongates Mn–O bonds and suppressed Jahn–Teller distortion. Additionally, the mesoporous structure provides more active sites and shortens the ion diffusion pathway, facilitating fast reaction kinetics. Consequently, the cathode exhibits a specific capacity of 420 mAh g^−1^ at 1 A g^−1^, superior cycling stability of 1000 cycles with a high-capacity retention of 90.3%. A carbon-coated MnO_x_ nanocomposite has also been developed by *in situ* generation from a low-crystallinity birnessite-type MnO [46]. The low crystallinity favors the Mn^2+^/Mn^4+^ redox, which enhances both the capacity and cycle life of the cathode. The nanosized MnO_x_ particles, combined with the high conductivity of the carbon substrate, promote efficient oxidation reactions. When paired with Mn^2+^-containing ZnSO_4_ electrolyte, the MnO_x_ cathode demonstrates an exceptional specific energy of 845.1 Wh kg^−1^ and a prolonged lifespan of 1500 cycles. Furthermore, a Mn-rich Mn_4_N cathode with a plasma-functionalized carbon nanotube (PCNT) interlayer (Fig. 3f) effectively captured Mn^2+^, making them concentrated near the cathode to maximize the utilization. This approach achieved an ultrahigh specific energy of 821.9 Wh kg^−1^ and 90% capacity retention after 9000 cycles without adding MnSO_4_ [149].

Cation doping is also widely used to fine-tune electronic structures and modify reaction pathways. Cu-doped ε-MnO_2_ (CMO) fabricated by electrodeposition showed enhanced solubility, complete dissolution-redeposition behavior, and suppressed irreversible phase transitions. This resulted in a higher capacity of 450.1 mAh g^−1^ at 0.2 A g^−1^ compared to undoped ε-MnO_2_ (282.4 mAh g^−1^) and improved cycling stability (Fig. 3g) [150]. Mo-doped δ-MnO_2_ was reported to enable an ultrahigh capacity of 652 mAh g^−1^ at 0.2 A g^−1^ via the Mn^4+^/Mn^3+^/Mn^2+^ route [159]. DFT calculations reveal that Mo^5+^ significantly alters the bond angles between Mn–O bonds, enhances the structural stability, and exacerbates the Jahn–Teller distortion in the [MnO_6_] octahedra in Mo-ZnMn_2_O_4_. This distortion promotes the dissolution of Mo-ZnMn_2_O_4_, releasing Mn^2+^ into the electrolyte. Mo doping also enhances the reactivity between Mn^3+^ and Mn^2+^, while improving the electronic conductivity of the electrode, collectively benefiting the overall performance of the cathode.

Similarly, a MnO_2_/MoO_3_ composite cathode was fabricated using a one-step hydrothermal method [151]. The incorporation of MoO_3_ reduced the Mn–O bond energy within MnO_2_, induced oxygen vacancies, and facilitated the spontaneous interaction between H^+^ and lattice oxygen during discharge. This process lowered the energy barrier for Mn release and enhanced the MnO_2_/Mn^2+^ redox reaction. The O bonded with Mo in the MnO_2_/MoO_3_ composite exhibits a more negative electrostatic potential (Fig. 3h), which promotes Mn^2+^ re-deposition. The capacity retention of MnO_2_ in the ZnSO_4_ electrolyte, without pre-added Mn^2+^ salts, increased from 62.7% to 92.6% after 300 cycles at 0.1 A g^−1^, corresponding to a calendar life exceeding 1900 h. Furthermore, the MnO_2_/MoO_3_ composite demonstrated excellent capacity retention of 80.1% after 16,000 cycles at 1 A g^−1^ (> 3200 h).

Co-doping or co-intercalation can yield synergistic effects. Na^+^ and Cu^2+^ co-intercalated δ-MnO_2_ (NCMO) activated the Cu^2+^ on the surface and the Mn^2+^/Mn^4+^ redox couple, leading to improved capacity and cycling stability. The smaller ionic radius of Cu^2+^ facilitates rapid diffusion into the electrolyte, enabling the Cu^0^/Cu^2+^ redox reaction during charge/discharge cycles. Cu^2+^ generated during charging enhances the Jahn–Teller distortion of the mesophase Zn_x_MnO(OH)_y_, thereby accelerating its surface dissolution. The co-intercalation of Na^+^ and Cu^2+^ ions, in combination with the deposition and dissolution of Zn_x_MnO(OH)_y_ and zinc hydroxide sulfate, allows the active materials to maintain sustained capacity at low mass loadings and exhibits a highly reversible Mn^2+^/Mn^4+^ redox process at high mass loadings. The cathode demonstrates an impressive specific capacity of 576 mAh g^−1^ after 100 cycles at a low loading of ~ 1 mg cm^−2^ and a high areal capacity of 2.10 mAh cm^−2^ at a high loading of ~ 10.9 mg cm^−2^ [160].

Co-doping of Cr^2+^ and Ni^2+^ into MnO_2_ (CrNi-MnO_2_) cathodes (Fig. 3i) was shown to induce lattice distortion in Mn(III)-intermediates, preventing “dead MnO_2_” formation and enabling a complete MnO_2_/Mn^2+^ redox [152]. Additionally, the CrNi-MnO_2_ cathode demonstrates a reduced Zn^2+^ diffusion barrier and enhanced structural integrity due to the synergistic effects of Cr and Ni co-doping. As a result, the cathode exhibited an ultrahigh capacity of 568 mAh g^−1^ at 0.2 A g^−1^, excellent rate performance (208 mAh g^−1^ at 2.0 A g^−1^), and superior cycling stability. Theoretical and experimental investigations also highlighted the role of Cr/Ni co-doping in modulating electron states and charge transfer kinetics, contributing to high-rate capability (10C) and stability (600 cycles) at an areal capacity of 10 mAh cm^−2^ [161].

Critical Assessment and Future Priorities for Mn-Based Cathodes: While universal optimization strategies, including oxygen vacancy engineering and robust carbon heterostructure construction, can effectively alleviate the sluggish reaction kinetics and Jahn–Teller induced structural degradation of Mn-based cathodes, the realization of the highly desirable two-electron Mn^4+^ ↔ Mn^2+^ redox capacity remains severely constrained by the strong dependence on corrosive acidic electrolytes. Because these acidic environments are intrinsically hostile to the Zn anode, inevitably triggering severe hydrogen evolution and premature full-cell failure, future research paradigms must pivot away from merely pursuing peak specific capacities in idealized coin cells. Instead, the paramount priority must shift toward fundamentally unlocking the 2e^−^ redox pathway within mild or neutral aqueous systems, while simultaneously engineering ultra-stable, acid-resistant anodic interphases to successfully translate these high-energy cathodes into practically viable PRZBBs. This united approach will pave the way for the widespread adoption of high-performance, safe, and cost-effective Zn-based batteries [162].

### Vanadium Oxides-Based Multi-Electron Transfer Zn-Based Batteries

Conventional vanadium-based cathodes in Zn-based batteries suffer from severe capacity fading, primarily due to the dissolution of vanadium species and the irreversible structural changes during cycling [163]. Moreover, their intrinsically low electronic conductivity and limited Zn^2+^ diffusion kinetics hinder rate capability [164]. To address these challenges, the construction of heterostructured cathodes through advanced approaches, such as metal/non-metal doping and defect engineering, has proved to be a promising strategy [165].

**Fig. 4:**
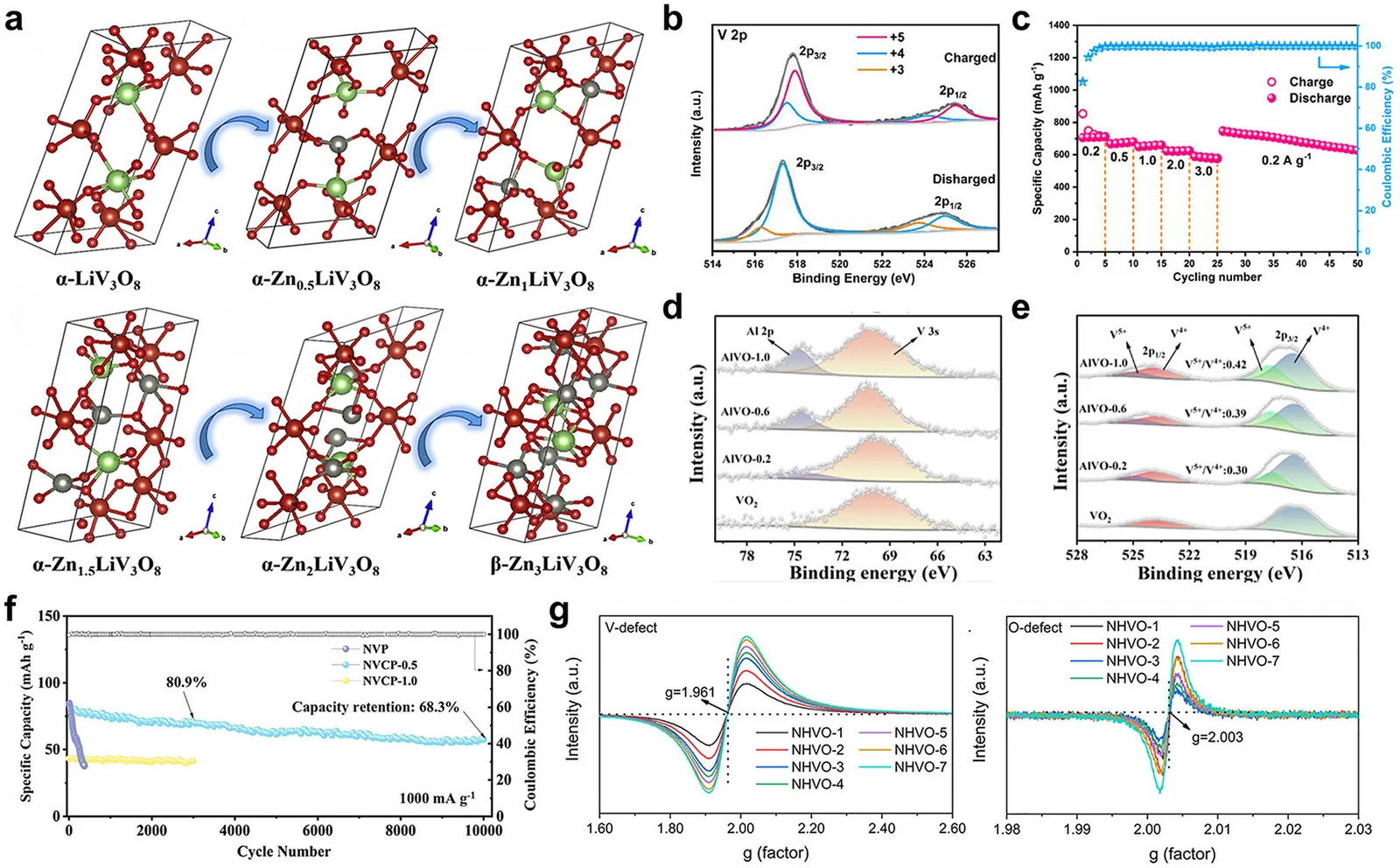
**a** Structural evolution mechanism of LiV_3_O_8_ during Zn^2+^ intercalation via DFT calculations. Reproduced with permission [167]. Copyright 2020, Elsevier. **b** V 2*p* XPS spectra of the electrode at fully charged and discharged states. Reproduced with permission [168]. Copyright 2021, Elsevier. **c** Rate performance of O-VN-based ZIBs. Reproduced with permission [168]. Copyright 2021, Elsevier. **d, e** XPS profiles of Al 2*p* and V 2*p* for both VO_2_ and AlVO. Reproduced with permission [170]. Copyright 2024, Elsevier. **f** Cycling performance of NVP, NVCP-0.5, and NVCP-1.0 at 1000 mA g^−1^. Reproduced with permission [171]. Copyright 2023, American Chemical Society. **g** EPR spectra recorded for O and V species in the as-prepared NHVO samples. Reproduced with permission [172]. Copyright 2024, Elsevier

Atomic doping and the design of novel vanadium-based cathode materials are critical for enhancing the performance of Zn-based batteries. These strategies effectively inhibit vanadium dissolution, improve the conductivity, and promote multi-electron redox reactions, ultimately boosting the specific capacity of battery [166]. Based on the V^3+^/V^5+^ couple, the lithium vanadium oxide (LiV_3_O_8_) undergoes two solid-solution reactions from α-LiV_3_O_8_ to α-Zn_0.5_LiV_3_O_8_ and to β-Zn_2_LiV_3_O_8_, which is separated by a two-phase transition from Zn_0.5_LiV_3_O_8_ to Zn_2_LiV_3_O_8_ and the other phase transformation from α-Zn_x_LiV_3_O_8_ (x < 2) to β-Zn_y_LiV_3_O_8_ (2 ≤ y ≤ 3) (Fig. 4a) [167]. This mechanism enables a 6e^−^ transition within LiV_3_O_8_ and causes minimal volumetric expansion of just 7.64%. As a result, the cathode achieves a capacity of 557.5 mAh g^−1^ with 85% capacity retention after 4000 cycles. The oxygen-doped vanadium nitride (O-VN) exhibits a cationic conversion reaction mechanism, which deviates from the conventional intercalation mechanism [168]. Under the influence of a strong electric field and mild acidic conditions, the O-VN cathode undergoes electrochemical oxidation, transforming into vanadium oxide and vanadium cations through *in situ* activation. During the discharge process, these cations are reduced to V_2_O_3_, which precipitates on the electrode surface. Upon charging, the V^3+^ ions are oxidized back into V^4+^/V^5+^ cations, dissolving into the electrolyte (Fig. 4b). This novel redox mechanism enables the ZIBs to deliver an ultrahigh discharge capacity of 705 mAh g^−1^. Furthermore, the cathode exhibits outstanding rate capability, maintaining a capacity of 581 mAh g^−1^ at 3 A g^−1^ (Fig. 4c). Another innovative approach is to utilize vanadium-based metal–organic frameworks (V-MOFs) as precursors to create a composite, where active vanadium nitride (VN) nanoparticles are uniformly distributed within an electrospun carbon nanofiber matrix. These nanoparticles transform in situ into a V_2_O_5_·nH_2_O phase during the initial charge process, which subsequently facilitates reversible Zn^2+^ intercalation and conversion reactions, leading to the formation of Zn_3_(V_2_O_7_)(OH)_2_(H_2_O)_2_ as the discharge product [169]. This architecture prevents the nanoparticle aggregation and provides a conductive and protective layer, thereby enabling exceptional cycling stability (482 mAh g^−1^ after 30,000 cycles at 50 A g^−1^) and ultrahigh-rate capability (297 mAh g^−1^ at 100 A g^−1^).

Cation doping can also directly modulate the valence state of vanadium. For instance, Al^3+^ doping into VO_2_ activates the V^4+^/V^5+^ redox couple via charge compensation, while the formation of stronger V^5+^-O and Al^3+^-O bonds enhances the structural stability and curtails dissolution (Fig. 4d, e) [170]. This doping strategy yields a specific capacity of 327.8 mAh g^−1^ at 0.1 A g^−1^ and surpasses the undoped material. Similarly, the Cr^3+^ doing into the NASICON-type Na_3_V_2-x_Cr_x_(PO_4_)_3_ can fully activate the V^4+^/V^3+^ and V^5+^/V^4+^ redox couples for 2e^−^ transfer reactions. The Cr^3+^ dopant aids in forming a robust cathode-electrolyte interphase, improves Zn^2+^ transport kinetics, and raises the average operating potential, resulting in a capacity of 84.4 mAh g^−1^ at 1 A g^−1^ with 68% capacity retention after 10,000 cycles (Fig. 4f) [171].

Defect engineering represents another powerful avenue for performance enhancement. Introducing defects into vanadium oxides increases the number of active sites, optimizes the electronic structures, and improves structural flexibility to accommodate volume changes, thereby facilitating multi-electron conversion. Oxygen-deficient V_2_O_5_ (V_2_O_5_-O_d_) nanosheets can expand the interlayer spacing of V_2_O_5_ and reduce the Zn^2+^ migration energy barrier, thereby facilitating Zn^2+^ diffusion [173]. Theoretical calculations further demonstrated that oxygen defects introduced an intermediate energy band and narrowed the bandgap of V_2_O_5_, which promoted electron transfer and enhanced reaction kinetics. Additionally, the formation energy of V_2_O_5_-O_d_ was significantly reduced, indicating a more stable framework structure. Consequently, the V_2_O_5_-O_d_ cathode exhibited a discharge capacity of 427.3 mAh g^−1^, a specific energy of 325.6 Wh kg^−1^, and 92.1% retention after 5000 cycles. A more sophisticated strategy involves the simultaneous introduction of both vanadium and oxygen defects into NH_4_V_4_O_10_ **(**NHVO, Fig. 4g) [172]. The synergistic effect of oxygen and vanadium defects promotes Zn^2+^ migration within the NHVO cathode by increasing active sites, lowering the migration barrier, and reducing the Gibbs free energy. Additionally, the introduction of O defects expands the valence change range of V, facilitating the occurrence of 2e^−^ reactions. As a result, the NHVO cathode achieved a specific capacity of 489 mAh g^−1^ at 0.5 A g^−1^ and capacity retention of 98% after 100 cycles.

Critical Assessment and Future Priorities for V-Based Cathodes: Although universally applicable strategies, including structural preintercalation and morphological nanoengineering, can effectively reduce the Zn^2+^diffusion barrier and stabilize layered VO_x_ frameworks, the exceptionally high capacities associated withV^5+^ ↔ V^3+^ multielectron transfer processes still require rigorous and critical evaluation. A substantial portion of these reported high capacities frequently stems from electrochemical artifacts, including parasitic water reduction and localized proton co-insertion, which are inevitably triggered by excessively wide voltage windows and often masked by impractically low mass loadings. To translate these promising chemistries into practical self-powered PRZBB devices, future research must urgently overcome the severe vanadium dissolution issue, which remains the fundamental Achilles heel of these systems. This necessitates a strategic paradigm shift toward engineering high mass loading electrodes and deploying advanced *operando* spectroscopy to definitively decouple genuine multi-valency redox chemistry from parasitic side reactions, ensuring true structural and electrochemical integrity under practical operating conditions [174].

### PBAs-Based Multi-Electron Transfer Zn-Based Batteries

**Fig. 5:**
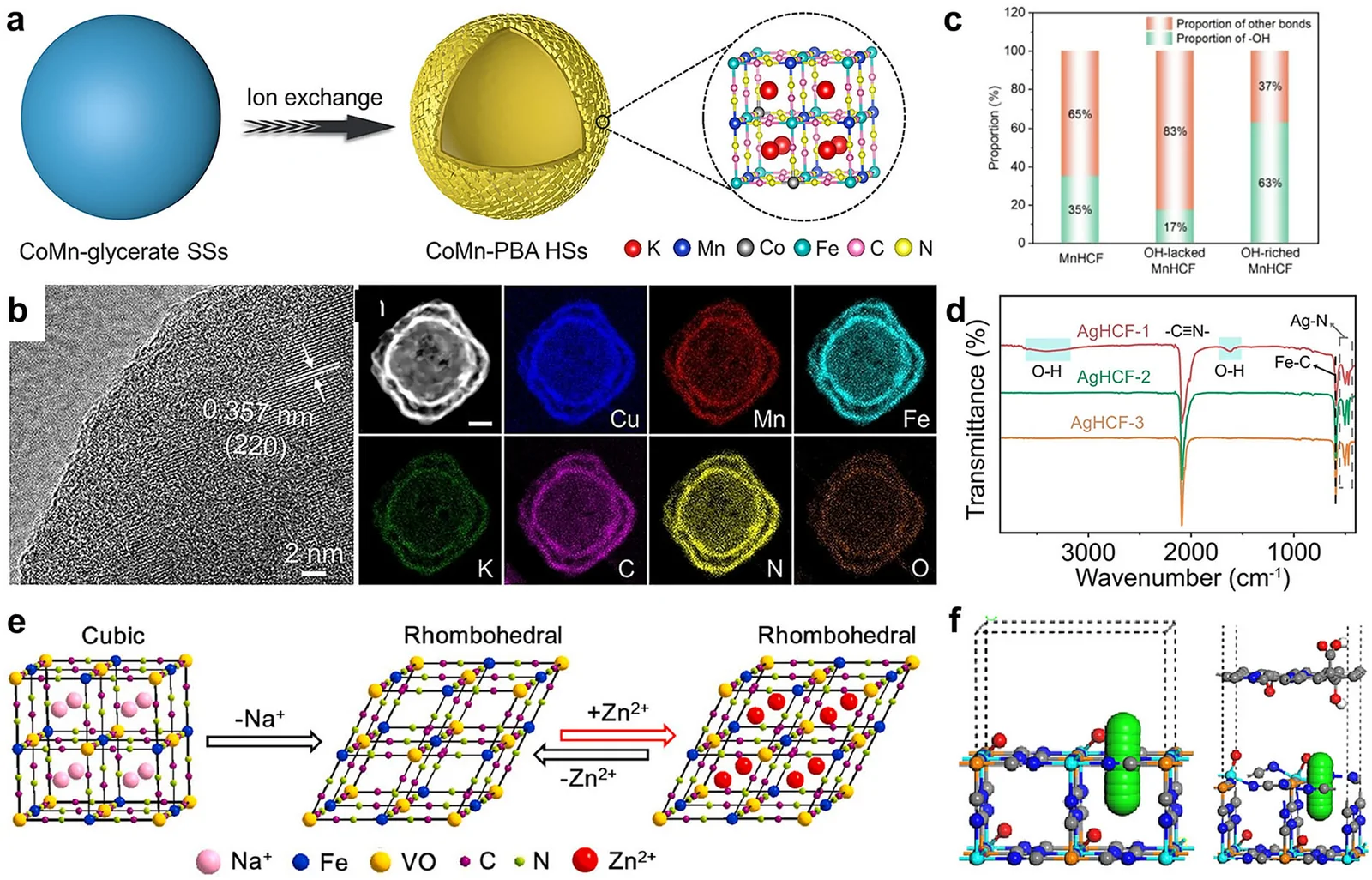
**a** Synthetic pathway for CoMn-PBA hollow spheres. Reproduced with permission [176]. Copyright 2021, Wiley–VCH. **b** HRTEM, HAADF-STEM, and elemental mapping images of CuMn-PBA. Reproduced with permission [177]. Copyright 2022, Wiley–VCH. **c** Comparative analysis of O-containing functional groups. Reproduced with permission [178]. Copyright 2023, Wiley–VCH. **d** FTIR spectra of AgHCF-1, AgHCF-2, and AgHCF-3. Reproduced with permission [109]. Copyright 2024, Wiley–VCH.** e** VHCF with dual redox-active sites as a cathode material for ZIBs. Reproduced with permission [179]. Copyright 2021, Elsevier. **f** Diffusion path of Zn^2+^ in KVO-HCF and GSAF@KVO-HCF. Reproduced with permission [180]. Copyright 2024, Wiley–VCH

Prussian blue analogs (PBAs), featuring a three-dimensional open framework, offer compelling advantages for ZIBs, including facile synthesis, resource abundance, low cost, and tunable operating potential [175]. However, their practical capacity is often limited by the presence of Fe(CN)_6_ vacancies, which reduces the number of active sites. To address this, compositional and structural engineering have been employed. Cobalt-substituted Mn-rich PBA hollow spheres (CoMn-PBA) were synthesized via a template ion-exchange method (Fig. 5a) [176]. The hollow structure not only provides a large surface area and sufficient active sites but also accommodates significant volume changes during ion insertion. Moreover, the Co substitution further stabilizes the crystal structure, leading to a specific capacity of 128.6 mAh g^−1^ at 0.05 A g^−1^ and 50.4 mAh g^−1^ at 2 A g^−1^, with a capacity retention of 76.4% after 1000 cycles.

Similarly, Cu-substituted Mn-PBA double-shelled nanoboxes (CuMn-PBA) were fabricated through tannic acid etching and cation exchange, which increased active centers and buffered the volume change (Fig. 5b). The partial Cu substitution also helped suppress Jahn–Teller distortion in Mn-N_6_ octahedra, contributing to a discharge capacity of 116.8 mAh g^−1^ at 0.1 A g^−1^ and 96.8% capacity retention over 2000 cycles [177]. Beyond structural design, activating inactive redox centers is another effective strategy. A hydroxylation strategy applied to manganese hexacyanoferrate (MnHCF) leverages hydroxyl groups adsorbed on Mn sites to continuously activate multiple redox centers (Fig. 5c), boosting the specific capacity to 136.1 mAh g^−1^ at 0.1 A g^−1^, with a specific energy of 228.8 Wh kg^−1^ [178].

Due to the easy formation of crystallization water during the PBA synthesis, the capacity degradation and inferior cycling performance are often observed. To address this issue, Ag was introduced into the ferrocyanide complex, and a novel Ag-based PBA of silver hexacyanoferrate (K_0.95_Ag_3.05_Fe(CN)_6_, AgHCF-3) was synthesized by co-precipitation at room temperature, which was free from vacancies and crystallization water (Fig. 5d) [109]. The open framework structure of AgHCF-3, with a large interlayer spacing of 7.11 Å, ensures a highly reversible Zn^2+^ (de)intercalation process, significantly improving Zn^2+^ storage capacity. AgHCF-3 undergoes a 4e^−^ transfer process based on the Ag^+^/Ag and Fe^3+^/Fe^2+^ redox couples. This material delivers a specific capacity of 179.6 mAh g^−1^ at 20 mA g^−1^, a specific energy of 200 Wh kg^−1^, and a capacity retention of 80.3% after 100 cycles. CoFe-PBAs significantly enhance the cycling stability of Zn-based batteries. Cobalt ions possess + 2 and + 3 oxidation states and can work in synergy with iron ions, thereby facilitating multi-electron transfer. The CoFe-PBA cathode exhibits a discharge platform of 1.75 V and a specific capacity of 173.4 mAh g^−1^ at 0.3 A g^−1^. Furthermore, VFe-PBA contains multivalent electron pairs such as Fe^2+^/Fe^3+^, V^3+^/V^4+^, and V^4+^/V^5+^, which can potentially undergo multi-electron transfer in Zn-based batteries. The VFe-PBA battery exhibits a specific capacity of 200 mAh g^−1^ at 0.5 A g^−1^. Using an aqueous eutectic electrolyte (5.55 mol L^−1^ glucose + 3 mol L^−1^ Zn(CF_3_SO_3_)_2_) with H_2_O, hydrogen evolution is suppressed through hydrogen bonding, while the deposition path of Zn is regulated, improving the cycling stability of the Zn anode. After 10,000 cycles at 10 A g^−1^, the VFe-PBA battery retains 55% of its capacity. Coating the surface of VFe-PBA nanoparticles with polypyrrole (PPy) enhances its conductivity and cycling stability [181]. However, due to the high solubility of vanadate ions in aqueous electrolytes, the cycling stability of VFe-PBA is relatively poor. The hexacyanovanadate cathode, which exhibits a typical nanoparticle morphology and a 3D lattice framework structure, undergoes a phase transition from the cubic phase to the rhombohedral phase after the first charge cycle [179]. Zn^2+^ can then be reversibly stored in the stable rhombohedral phase (Fig. 5e). The VFe-PBA demonstrates a specific capacity of 187 mAh g^−1^ at 0.5 A g^−1^ and still retains 122 mAh g^−1^ at 5 A g^−1^. By using V_6_O_13_ nanobelts as the vanadium source, a high crystallinity VFe-PBA nanocube is synthesized through an *in situ* conversion strategy, significantly improving the cycling stability of Zn-based batteries [175]. The VFe-PBA cathode maintains a specific capacity of 173 mAh g^−1^ at 0.1 A g^−1^, with 85% capacity retention after 500 cycles.

A novel three-dimensional conductive structure, GSAF@KVO-HCF, composed of graphene self-assembled fibers (GSAF) and potassium hexacyanovanadate (KVO-HCF), is introduced [180]. In GSAF@KVO-HCF, the KVO-HCF particles are dispersed at the nanoscale and can provide storage sites for Zn^2+^. Compared to the pristine KVO-HCF cathodes, the tight integration of PBAs with three-dimensional carbon reduces the Zn^2+^ transport distance and accelerates charge transfer (Fig. 5f). Additionally, the large surface area and porous structure of GSAF help mitigate the dissolution of vanadium in the electrolyte. The Zn||GSAF@KVO-HCF battery maintains a specific capacity of 103 mAh g^−1^ after 1000 cycles at 1 A g^−1^.

Managing phase transitions is of paramount importance for the long-term stability in PBAs. A core–shell double-atom PBA structure was constructed by epitaxially growing Fe-doped manganese hexacyanoferrates, which creates a protective three-phase heterostructure (MnHCF/FeHCF/amorphous layer) during cycling [182]. This design minimizes the structural distortion and Mn dissolution, enabling a capacity of 166.3 mAh g^−1^ at 0.1 A g^−1^ and 83.9% capacity retention after 4800 cycles at 2 A g^−1^.

The potential of PBAs as cathode materials for ZIBs lies in their high specific capacity and tunable structures. However, their main challenges are inferior rate performance and cycling stability, particularly under prolonged use and high-rate discharge conditions. During extended cycling, structural changes or material degradation may occur, which can compromise long-term stability. Modifications such as material doping or other engineering strategies can help enhance their electrochemical performance [183].

### Organic Electrodes-Based Multi-Electron Transfer Zn-Based Batteries

Organic materials have been emerging as a promising type of non-metal electrodes for sustainable energy storage, due to their abundant resources, low cost, and excellent environmental-friendliness. Based on their charge storage mechanisms, organic electrode materials include n-type, p-type, and bipolar electrode materials [184]. During the redox reaction, n‑type materials are reduced to anionic states, while p‑type materials are oxidized to cationic states. Bipolar materials combine the features of both, enabling bidirectional ion storage and release within a single electrode, which enhances the overall capacity and cycling stability of Zn-based batteries.

#### N-Type Electrode

Common n-type organic electrode materials include C=O, C=N, –NO_2_, and others [185–187]. During discharging, these groups gain electrons and are reduced to an anionic state, which then interacts with Zn^2+^ for charge compensation [188–190]. By employing molecular engineering strategies to integrate various redox-active centers within a single organic molecule, multiple electron transfers can be achieved, thereby enhancing the specific capacity of Zn-based batteries [191–193].

##### C=O-Based Electrode Materials

Organic electrode materials containing C=O groups offer advantages such as high theoretical capacity, good reversibility, and fast redox kinetics, making them one of the most prevalent electrodes in Zn-based batteries (Table 3) [116]. The C=O group facilitates charge storage for Zn^2+^ through enolization reaction processes [117].

**Table 3:** Summary of organic electrode materials based on C=O structure

Materials	Electrolyte	Current density (A g^−1^)	Specific capacity (mAh g^−1^)	Cycle life	Capacitance retentione	References
PQ	2 mol L^−1^ ZnSO_4_	0.054	250	36,000	96.3% at 5 A g^–1^	[53]
Ph-tri-BQ	4 mol L^−1^ Zn(ClO_4_)_2_	100	221	150,000	143 mAh g^−1^ at 100 A g^–1^	[54]
PQ-Δ	3 mol L^−1^ Zn(CF_3_SO_3_)_2_	0.15	210	500	99.9% at 0.15 A g^–1^	[55]
Cu-TBPQ MOF	3 mol L^−1^ Zn(CF_3_SO_3_)_2_	0.05	371.2	500	88% at 2 A g^–1^	[56]
TDT	1 mol L^−1^ ZnSO_4_	0.2	369	3,000	148 mAh g^−1^ at 10 A g^–1^	[57]
C4Q	1 mol L^−1^ Al(OTF)_3_	0.8	300	50	81% at 0.2 A g^–1^	[58]

**Fig. 6:**
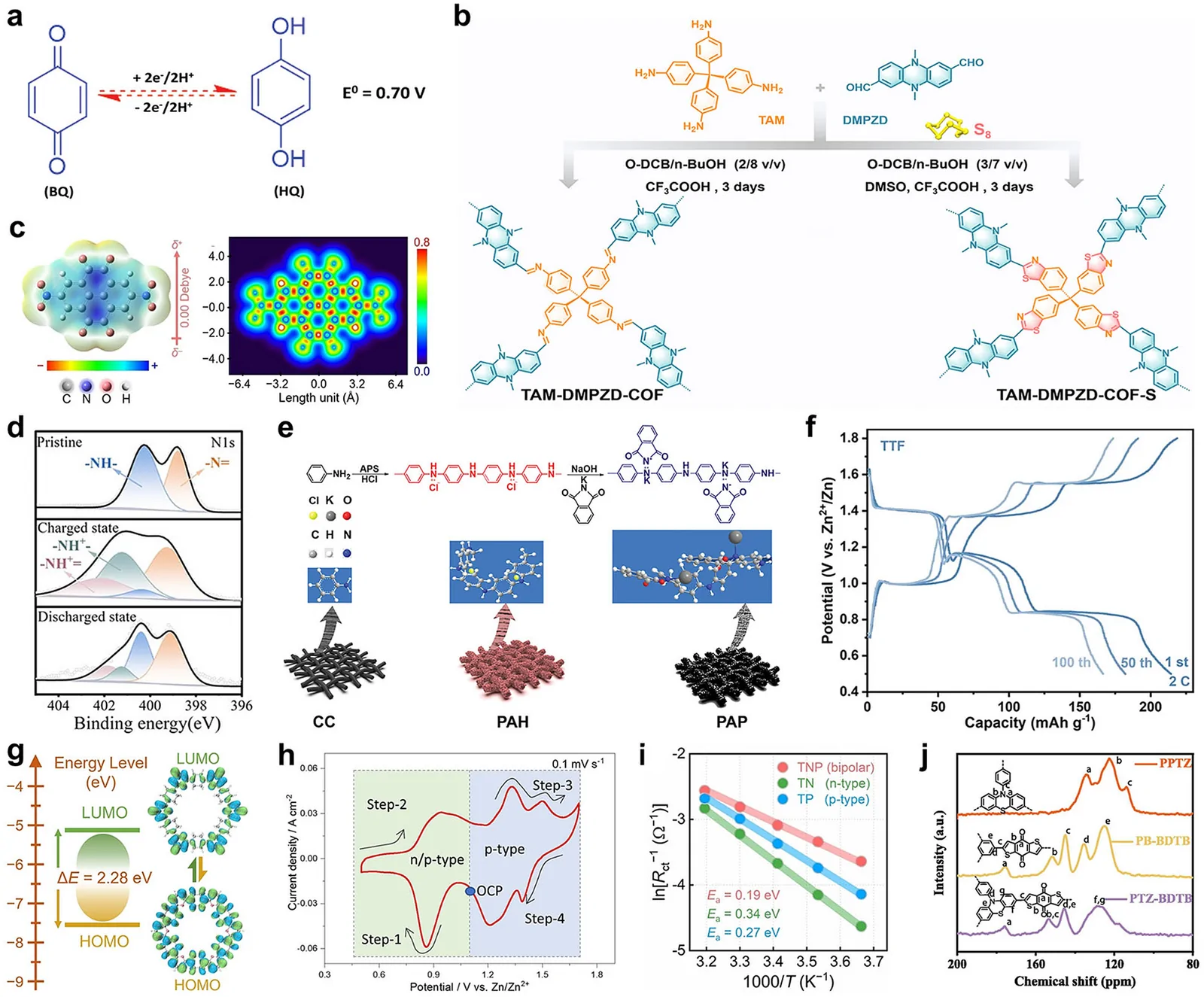
**a** Schematic illustration of the 2e^−^/2H^+^ redox mechanism in the BQ/HQ organic couple. Reproduced with permission [194]. Copyright 2024, Wiley–VCH. **b** Synthesis route of TAM-DMPZD-COF and TAM-DMPZD-COF-S. Reproduced with permission [61]. Copyright 2025, Elsevier. **c** Molecular electrostatic potential (ESP) simulation and localized orbital locator-π (LOL-π) analysis. Reproduced with permission [196]. Copyright 2023, Wiley–VCH. **d** Ex situ N 1*s* XPS spectra under different states. Reproduced with permission [65]. Copyright 2024, Wiley–VCH. **e** Schematic illustration of the fabrication process for PAP electrode material. Reproduced with permission [67]. Copyright 2024, Wiley–VCH. **f** GCD profile of TTF electrode at 2C. Reproduced with permission [72]. Copyright 2023, Nature Publishing. **g** Frontier molecular orbitals and energy levels of AOSs. Reproduced with permission [74]. Copyright 2024, Wiley–VCH. **h** CV curve of Zn||PTD-1 battery at a scan rate of 0.1 mVs^−1^. Reproduced with permission [75]. Copyright 2021, Wiley–VCH. **i** Calculated activation energy (*E*_a_) for interfacial charge migration within various organics. Reproduced with permission [78]. Copyright 2024, Wiley–VCH. **j** Solid state ^13^C NMR spectra of PPTZ, PB-BDTB, and PTZ-BDTB. Reproduced with permission [79]. Copyright 2025, Elsevier

Organic molecules containing multiple C=O groups can increase the number of electron transfers, thereby enhancing the specific capacity of Zn-based batteries. For example, the macrocyclic quinone (C4Q) based on four cross-linked anthraquinone units enables a 7e^−^ transfer [58]. The 9,10-anthraquinone (PQ) molecule, known for its structural stability and poor solubility in aqueous electrolytes, is considered an ideal cathode material for Zn-based batteries [53]. At a current density of 0.1 A g^−1^, PQ exhibits a specific capacity of 150.0 mAh g^−1^, and it still retains 96.3% capacity retention after 36,000 cycles at 5 A g^−1^. Benzohydroquinone (BQ) and hydroquinone (HQ) are simple small-molecule quinones that are widely used as organic cathodes for various metal-ion batteries [194]. The redox reactions of BQ and HQ involve 2e^−^ transfer (Fig. 6a). By mixing equal proportions of BQ and HQ, an electrode material with multiple active sites is created. The resulting electrode exhibits a specific capacity of 209.7 mAh g^−1^ at 10C, maintaining 86.4% capacity retention after 3000 cycles. By using phenyl-linked benzohydroquinone (Ph-tri-BQ), which can accommodate up to six Zn^2+^, a high specific capacity of 402 mAh g^−1^ is achieved [54]. Moreover, Ph-tri-BQ has significantly reduced solubility in solution, providing an exceptionally long cycle life of over 150,000 cycles. The redox-active triangular phenanthraquinone macrocycle (PQ-Δ) exhibits enhanced stability due to its rigid geometric conformation and layered architecture [55]. The π-conjugation in PQ-Δ promotes electron delocalization between the reduced forms of PQ units, free radical anions (PQ^−^), and dianions (PQ^2−^), enabling 6e^−^ transfer. The PQ-Δ electrode exhibits a specific capacity of 225 mAh g^−1^ at 30 mA g^−1^, retaining 99.9% capacity retention after 500 cycles. Additionally, quinone-rich polydopamine (PDA) has been used as a cathode material for Zn-based batteries, where the quinone groups serve as redox centers that can reversibly coordinate with Zn^2+^ [195]. The resulting cathode achieves a specific capacity of 372.3 mAh g^−1^ at 50 mA g^−1^.

By introducing additional functional groups into C=O-based organic molecules, the number of redox-active centers can be increased, thereby enhancing the specific capacity of cathodes. For example, 2,3,7,8-tetraamino-5,10-dihydrobenzoquinone-1,4,6,9-tetraone (TDT) contains four C=O active centers [57]. The p-electrons on the nitrogen atoms conjugate with the quinone rings, allowing electrons to delocalize throughout the molecule, which enhances the electrical conductivity of the material. The TDT cathode exhibits a specific capacity of 369 mAh g^−1^ at 0.2 A g^−1^ and retains 182 mAh g^−1^ at 10 A g^−1^. The methoxy groups in 2,6-dimethoxy-1,4-benzoquinone (m-DMBQ) form a p-π conjugated structure with the benzoquinone, which not only reduces the bandgap of m-DMBQ but also lowers the energy level of its lowest unoccupied molecular orbital (LUMO), thus increasing the discharge potential of Zn-based batteries [197]. The m-DMBQ electrode achieves a specific capacity of 312 mAh g^−1^, close to the theoretical limit, with a specific energy of 275 Wh kg^−1^.

Integrating quinones into a two-dimensional copper-based MOF (Cu-TBPQ) effectively solves the issue of quinone dissolution during battery cycling [56]. This integration not only generates a dense network of redox-active sites but also imparts high conductivity and a stable framework structure, while providing open channels for Zn^2+^ transport. The CV curve shows two redox peaks at 0.45/0.71 V and 0.88/1.17 V, where the 0.88/1.17 V peak corresponds to the Cu^2+^/Cu^+^ redox, and the 0.45/0.71 V peak represents the structural change between C=O and C–O^−^. In total, this electrode enables 3e^−^ transfer per molecule, which delivers a reversible capacity of 371.2 mAh g^−1^ at 50 mA g^−1^.

Organic materials with C=O structures offer high capacity and high rates [198]. However, they also face challenges such as the dissolution issue rooted in the C–O^−^ groups, which can reduce the cycling stability of electrodes. To address this, several molecular engineering strategies have proved effective. Increasing molecular weight and extending π-conjugation, as demonstrated by macrocyclic quinones and polymeric frameworks, inherently reduces solubility by raising the energetic cost of solvation. Introducing hydrophobic substituents or charged functional groups can further suppress dissolution by repelling water or immobilizing the active material through electrostatic interactions and hydrogen-bonding networks. Integrating quinone units into extended covalent networks, such as the two-dimensional Cu-TBPQ MOF discussed above, locks the redox-active sites into an insoluble framework while maintaining open channels for Zn^2+^ transport. These approaches, which can be combined within a single material system, provide a rational basis for designing dissolution-resistant C=O-based cathodes.

##### C=N-Based Electrode Materials

C=N-based compounds, which are categorized as n-type materials, exhibit outstanding redox activity in Zn-based batteries (Table 4) [199]. Compared to C=O bonds, compounds with C=N bonds are more prone to forming Schiff bases, offering higher efficiency [118]. Nitrogen, with its lower electronegativity compared to oxygen, reduces the electrostatic interactions between Zn^2+^ and the cathode, thereby promoting the diffusion of Zn^2+^. Compared to other organic materials, imine compounds synthesized through solvothermal condensation reactions between amine and carbonyl groups demonstrate higher structural flexibility, which provides more active sites and extends the π-conjugated system, thereby resulting in exceptional capacity and cycling stability [200].

**Table 4:** Summary of organic electrode materials based on C=N structure

Materials	Electrolyte	Current density (A g^−1^)	Specific capacity (mAh g^−1^)	Cycle life	Capacitance retentione	References
DQH	3 mol L^−1^ NH_4_CF_3_SO_3_	0.5	385	30,000	73.2% at 10 A g^−1^	[59]
QDPA	2 mol L^−1^ ZnSO_4_	0.1	340.9	13,000	99.998% at 20 A g^−1^	[60]
TAM-DMPZD-COF-S	–	0.1	325	50,000	92% at 10 A g^−1^	[61]
PA-COF	1 mol L^−1^ ZnSO_4_	0.1	247	10,000	0.38% capacity decay per cycle at 1 A g^−1^	[62]
TAPQ	1 mol L^−1^ ZnSO_4_	0.05	443	250	92% at 0.05 A g^−1^	[63]
HTPT-COF@CNT	1 mol L^−1^ lithium bis(trifluoromethanesulfonyl)imide	20	247.8	1000	77.4% at 5 A g^−1^	[64]

N-heterocyclic dipyrazino[2,3-f:2′,3′-h]quinoxaline-2,3,6,7,10,11-hexacarbonitrile (DQH) features a π-conjugated aromatic planar structure [59]. The DQH molecule contains six imine and cyano functional sites and can enable 6e^−^ transfer. The DQH cathode achieves a specific capacity of 385 mAh g^−1^ at 0.5 A g^−1^. Quinoxalino[2,3-i]diquinoxalino[2′,3′:6,7]quinoxalino[2,3-a:2,3-c]phenazine (QDPA) effectively suppresses the material dissolution issue, due to its high molecular weight and stable π-π stacking structure [60]. As a result, The QDPA cathode exhibits only a 0.002% capacity degradation after 13,000 cycles at 20 A g^−1^. The QDPA electrode accommodates three Zn^2+^ ions with 6e^−^ transfer, which corresponds to a specific capacity of 340.9 mAh g^−1^ at 0.1 A g^−1^. 5,7,12,14-Tetrazolo-6,13-pentene (TAPQ) possesses a quinone/pyridine hybrid structure, which enables 6e^−^ transfer with a theoretical capacity of 515 mAh g^−1^ [63]. At a current density of 50 mA g^−1^, TAPQ achieves a reversible capacity of 443 mAh g^−1^. Furthermore, its π–π structure reduces the dissolution during charge/discharge and thus improves the cycling stability. The TAPQ electrode retains 92% capacity retention after 250 cycles. Dibenzo[a,c]dibenzo[5,6:7,8]quinoxalino[2,3-i]phenazine-10,21-dione (TABQ-PQ) exhibits a π-conjugated plane, providing an ultralong cycling life of over 30,000 cycles [201]. The C=O and C=N redox centers in TABQ-PQ support 6e^−^ transfer, delivering a discharge capacity of 181.9 mAh g^−1^ at 0.2 A g^−1^. Benzo[i]benzo-[6,7]quinoxalino[2,3,9,10]phenanthrol[4,5-abc]phenazine-5,10,16,21-tetraone (PTONQ) facilitates 8e^−^ transfer, resulting in a higher specific capacity of 224.7 mAh g^−1^ at 175 mA g^−1^ [202].

By introducing sulfur into the reaction system, the 4-connected Td 4,4′,4′′,4′′′-methanetetrayltetraaniline molecule can react with 2-connected 5,10-methyl-5,10-dihydrophenazine-2,7-dicarbaldehyde, therefore converting the imine bonds into aromatic thiazole units. This reaction results in the thiazole-linked 3D COF (TAM-DMPZD-COF-S) (Fig. 6b) [61], which can accommodate up to 12e^−^, significantly enhancing the specific capacity. The condensation reaction of hexaketone with 2,3,7,8-phenazine tetraamine yields a pyrroline-based covalent organic framework (PA-COF), which exhibits a specific capacity of 247 mAh g^−1^ at 0.1 A g^−1^ [62]. By coupling 2,3,7,8-tetraamine-1,4,6,9-tetraone with hexaketone cyclohexane octahydrate, a two-dimensional fully conjugated covalent organic framework (HTPT-COF) rich in C=O and C=N groups was successfully synthesized [64]. Density functional theory (DFT) calculations reveal that the HTPT-COF redox reaction occurs in four steps, with the transfer of a total of 24e^−^.

Although the C=N bond theoretically demonstrates excellent redox potential, there is a risk of bond cleavage or reorganization during the charge–discharge cycles, which can reduce the Zn-based batteries lifespan. Beyond this chemical stability concern, the dissolution of C=N-containing molecules into the aqueous electrolyte also poses a challenge for cycling stability. The formation of covalent organic frameworks, such as TAM-DMPZD-COF-S and PA-COF, represents a particularly effective countermeasure, as it covalently locks the imine-based redox units into an insoluble extended structure while retaining sufficient porosity for Zn^2+^ diffusion. Engineering strong π–π stacking interactions, as exemplified by the ultralong cycle life of QDPA, offers an alternative supramolecular route to resist solvation. Future research will focus on combining C=N with other redox-active sites, such as C=O, and on integrating these groups into framework or supramolecular architectures to overcome the current limitations and promote the practical application of this class of materials.

##### –NO_2_-Based Electrode Materials

Nitro groups exhibit strong electron-withdrawing effects, which not only enhance the redox reactivity of Zn-based batteries but also provide higher redox potential, thereby improving the specific energy of electrodes [203]. Nitro groups can accept two electrons, enabling effective charge storage. O-Dinitrobenzene (o-DB), m-dinitrobenzene (m-DB), and p-dinitrobenzene (p-DB) have been used as electrode materials in Zn-based batteries [120]. With 6e^−^ transfer, these electrodes achieve specific capacities of 338, 302, and 402 mAh g^−1^, respectively. p-DB materials also demonstrate high cycling stability of 25,000 cycles and an energy density of 230 Wh kg^−1^ at 5 A g^−1^. 2,7-Dinitropyrene-4,5,9,10-tetraone (DNPT) contains eight redox-active centers, four of which are electron-withdrawing carbonyl groups and two are nitro groups, providing dual redox-active sites for charge chelation (Fig. 6c) [196]. During discharge, the carbonyl groups first coordinate, followed by coordination with the nitro groups. The DNPT electrode exhibits a specific capacity of 320 mAh g^−1^ and a cycling life of 60,000 cycles. Tetranitroporphyrin (TNP) features 4 electron-accepting nitro groups and 2 electron-donating amine groups, corresponding to a total transfer of 10e^−^ [78]. The TNP cathode exhibits a platform voltage of 1.08 V, a specific capacity of 338 mAh g^−1^, a specific energy of 365 Wh kg^−1^, and a capacity retention of 71.6% after 50,000 cycles at 10 A g^−1^.

Nitro-containing organic materials show significant potential for Zn-based batteries applications, especially in enhancing the energy density of the batteries. However, challenges remain in terms of cycling stability and capacity retention. Future research can address these issues through material design, organic–inorganic composites, electrolyte optimization, and other approaches, facilitating their widespread application in Zn-based batteries.

#### P-Type Electrode

P-type electrodes typically exhibit higher redox potentials compared to n-type electrodes, a characteristic that enhances the energy density of Zn-based batteries [204]. Furthermore, p-type electrodes do not involve the reorganization of chemical bonds during electrochemical reactions, which generally results in faster redox kinetics (Table 5).

**Table 5:** Summary of organic electrode materials based on p-type electrode

Materials	cc	Current density (A g^−1^)	Specific capacity (mAh g^−1^)	Cycle life	Capacitance retentione	References
DHTAT	3 mol L^−1^ Zn(ClO_4_)_2_	0.05	224	5000	73% at 5 A g^−1^	[65]
m-PTPA	2 mol L^−1^ ZnCl_2_	0.5	210.7	1000	87.6% at 6 A g^−1^	[66]
PAP- PANI	2 mol L^−1^ Zn(OTF)_2_	0.2	114.8	25,000	76.1% at 1 A g^−1^	[67]
PTZAN	0.5 mol L^−1^ Zn(OTF)_2_ (TMP: EC: DEC = 1: 1: 1)	0.1	145	2000	77% at 1 A g^−1^	[68]
Q-PANI	1 mol L^−1^ ZnSO_4_	0.2	186	1500	88% at 2 A g^−1^	[69]
dNPC	20 mol L^−1^ Li-N(SO_2_CF_3_)_2_ + 1 mol L^−1^ Zn(N(SO_2_CF_3_)_2_)_2_ (LiTFSI and Zn(TFSI)_2_	0.05	109	1000	96% at 0.5 A g^−−1^	[70]
DMPZ	0.5 mol L^−1^ Zn(OTf)_2_ and 1 mol L^−1^ NaClO_4_	0.051	231	5000	80% at 1.275 A g^−1^	[71]
TTF	3 mol L^−1^ Zn(ClO_4)2_·6H_2_O	2 C	220	10,000	68.8% at 20 C	[72]
PANI-S	1 mol L^−1^ ZnSO_4_	2	180	2000	110 mAh g^−1^ 0 A g^−1^	[73]

##### Aniline-Based Electrode Materials

Aniline-based materials have garnered significant attention due to their high theoretical capacity. Furthermore, electrodes with aniline structures exhibit excellent reaction reversibility, significantly enhancing the cycling life of Zn-based batteries [205]. Additionally, the synthesis of aniline-based materials is versatile, including methods such as chemical polymerization, electrochemical polymerization, and templating techniques. The diverse synthesis approach offers various options for tailoring electrodes with tunable morphologies and properties, thereby allowing researchers to optimize materials based on the particular needs or requirements [158, 206, 207].

Using a simple solid-state synthesis method, an organic material of 5,12-dihydro-5,6,11,12-tetraazatetracene (DHTAT) was successfully developed [65]. DHTAT features a high degree of conjugation and an extended aromatic molecular structure, effectively reducing its solubility in the electrolyte. The charge storage mechanism of DHTAT primarily relies on the redox reaction of its -NH- groups, corresponding to a 2e^−^ transfer reaction (Fig. 6d). The DHTAT cathode exhibited a specific capacity of 224 mAh g^−1^ at 50 mA g^−1^. The diazabutadiene (N−C=C−N) structure in the 5,10-dihydro-5,10-dimethylphenazine (DMPZ) molecule serves as its redox-active center [71]. During charging, DMPZ undergoes a two-electron transfer reaction and exhibits sequential oxidation potential at 1.1 V and 1.72 V *vs.* Zn/Zn^2+^. This cathode achieved a specific capacity of 231 mAh g^−1^ at 51 mA g^−1^, with 81% capacity retention after 1000 cycles. By employing an in situ solid-state electrochemical oxidation strategy, N-phenyl carbazole (NPC) was electrochemically transformed into reversible dicarbazolyl compounds (dNPC) [70]. During oxidation, NPC forms highly reactive radical cations, which then couple with another cation or the parent carbazole molecule to form stable dicarbazole derivatives. These derivatives undergo two reversible single-electron oxidation processes, yielding divalent cations (dNPC^2+^), which can be reduced back to neutral dicarbazole during discharge. The dNPC/dNPC^2+^ couple exhibits excellent performance, with an average operating voltage of 1.3 V, a specific capacity of 109 mAh g^−1^, and a capacity retention of 96% after 1000 cycles.

By introducing phthalimide, which possesses a π-conjugated structure and nitrogen atoms with lone-pair electrons, as a dopant in polyaniline (PANI), the electrode’s conductivity and cycling stability were significantly enhanced [67]. Specifically, the large size of K^+^ (0.138 nm) increases steric hindrance, reducing chain entanglement and thus improving the structural stability of the electrode (Fig. 6e). The phthalimide anion adjusts the local electron cloud density of the electrode, effectively lowering defect concentration and forming a stable triangular coordination structure. The –N= and –NH– groups in the PANI molecular chain undergo reversible protonation and deprotonation reactions during charging and discharging. The cathode delivered a discharge capacity of 114.8 mAh g^−1^ at 0.2 A g^−1^, and it maintained 76.1% capacity retention after 25,000 cycles at a current density of 1 A g^−1^.

Electrochemical methods were also used to introduce quinone-type active sites into PANI while simultaneously altering the conformation of its polymer chains, enhancing the electrochemical performance of Zn-based batteries [69]. During electrochemical treatment, PANI was oxidized to pernigraniline state, which then reacted with water in the electrolyte to form quinone end groups. This process not only introduces quinone-type active sites but also converts the PANI chain from a compact helical conformation to an expanded helical form, improving both the electrode’s conductivity and charge transfer kinetics. The treated PANI exhibited two pairs of redox peaks, corresponding to the oxidation/reduction of both PANI itself and the quinone active sites, thus increasing the electron transfer number in the system. This PANI cathode achieved a specific capacity of 186 mAh g^−1^ at 0.2 A g^−1^, with 88% capacity retention after 1500 cycles at 2 A g^−1^.

By doping PANI with self-doped sulfonic acid groups (PANI-S), high proton concentration around the polymer backbone is maintained, which facilitates the electrode’s redox process and preserves its electrochemical activity [73]. This strategy overcomes the issue of mismatch between the conventional PANI cathode and the Zn anode under active conditions. During discharge, PANI-S undergoes two reduction steps (*R*_1_ and *R*_2_). In the first reduction step (*R*_1_), protonated nitrogen (–NH^+^= and –NH^+^–) is reduced to -NH-, releasing SO_4_^2−^ to balance the charge. In the second reduction step (*R*_2_), the newly formed -NH^+^ is further reduced, releasing more SO_4_^2−^. As a result, each PANI unit can achieve 2e^−^ transfer. The Zn||PANI-S batteries exhibit a specific capacity of 180 mAh g^−1^ and maintains 130 mAh g^−1^ at 10 A g^−1^ with 2000 cycles of excellent cycling.

Nitrogen-rich conjugated microporous polytriphenylamine (m-PTPA), with a COF-like porous structure, facilitates fast Zn^2+^ transfer, thereby improving the specific energy and cycling stability of electrodes [66]. The redox process in m-PTPA involves a four-step Cl^−^ storage mechanism. Specifically, nitrogen sites in m-PTPA gradually bind with Cl^−^ during charging, forming a five-step anion storage process. Despite the theoretical binding of up to five Cl^−^ ions, the actual number is about four due to the open-framework structure of m-PTPA. This mechanism allows m-PTPA to achieve a specific capacity of 210.7 mAh g^−1^ and excellent capacity retention of 87.6% after 1000 cycles.

Although aniline-based materials initially exhibit excellent performance, their capacity degradation may significantly accelerate over prolonged cycling, due to the substantial volume changes during charge and discharge. These volume changes can lead to structural damage of the electrode material, adversely affecting the cycling stability and mechanical properties of the electrode, thus limiting their use in high-power applications. To overcome these drawbacks, future strategies may involve doping, composite formation, or integration with other high-performance materials (such as metal oxides, carbon-based materials, etc.) to enhance the electrochemical performance of aniline-based materials [208].

##### Sulfur-Containing Compounds

Sulfur-containing organic compounds typically exhibit high theoretical capacities, as sulfur atoms can engage in reactions with multiple electrons. These compounds can undergo multi-electron reactions, significantly enhancing the specific energy of cathodes [209, 210]. Through chemical synthesis, a variety of sulfur-containing organic compounds can be designed and prepared, with their molecular structures adjusted to optimize the electrochemical performance of Zn-based batteries [211–213].

Tetrathiafulvalene (TTF) is a typical sulfur-containing p-type organic electrode material [72]. During charging, the strength of the C=C bonds in its molecular structure weakens, while the strength of C–C and C=S bonds increases, indicating a redistribution of electrons during the oxidation process. To balance the charge, anions like ClO_4_^−^ can be stored. Upon discharge, these changes are reversed, as the C=C bond strength recovers and the C–C and C=S bond strengths decrease, enabling a 2e^−^ transfer. The cathode exhibits a specific capacity of 220 mAh g^−1^ at 2C, an average discharge voltage of 1.05 V, and a specific energy of 213 Wh kg^−1^ (Fig. 6f). TTF also demonstrates excellent rate performance and long-term cycling stability, retaining 68.8% of its capacity after 10,000 cycles at 20C. The designed 4,4'-(10H-phenothiazine-3,7-diyl) bis(N,N-diphenylaniline) (PTZAN) material undergoes a 4e^−^ transfer during charging [68]. The first electron transfer occurs early in the charge cycle, where the C–N–H bond in the phenothiazine heterocycle loses an electron and a proton (H^+^), forming a C=N bond. Subsequently, a neutral sulfur atom (–S–) loses another electron, becoming a positively charged sulfur atom, marking the second electron transfer. At this stage, the positively charged PTZAN electrode must adsorb an anion to balance the charge. The third and fourth electron transfers occur in further charging stages, where the neutral nitrogen atoms (–N–) in the two triphenylamine units lose two electrons and become positively charged (i.e., –N⁺–). These positively charged nitrogen atoms require the adsorption of two additional anions to balance the charge. This multi-electron transfer mechanism translates to a specific capacity of 145 mAh g^−1^ at 0.1 A g^−1^, which is close to its theoretical capacity 156.3 mAh g^−1^. This cathode also demonstrates a capacity retention of 77% after 2000 cycles.

Sulfur-containing compounds hold significant potential as cathode materials for Zn-based batteries, particularly in terms of high energy and tunability. However, it is important to note that some sulfur-containing compounds exhibit poor conductivity, which necessitates molecular design or composite integration with conductive materials [214–217]. Dissolution of small organosulfur molecules can further limit cycling stability, a challenge that can be mitigated by extending the conjugated backbone, forming polymeric structures, or anchoring the active material onto conductive carbon substrates. With continuous advancements in material design, the prospects of sulfur-containing compounds in Zn-based batteries remain promising, especially in high-performance battery systems [218, 219].

#### Bipolar Organic Electrode Materials

N-type materials typically exhibit lower potentials and higher capacities, while p-type materials tend to show higher voltages but lower capacities. In stark contrast, bipolar organic compounds containing both n-type and p-type redox groups are able to combine the advantages of both, compensating for the shortcomings of their respective monopolar counterparts [220]. Consequently, bipolar organic materials possess higher discharge plateaus and greater capacities, enhancing the overall performance of Zn-based batteries by increasing the number of redox-active sites through oxidation and exchange at the positively charged electrode (Table 6). This enables a comprehensive improvement in the electrochemical performance of the electrodes [78, 221, 222].

**Table 6:** Summary of organic electrode materials based on Bipolar organic electrode materials

Materials	Electrolyte	Current density (A g^−1^)	Specific capacity (mAh g^−1^)	Cycle life	Capacitance retention	References
AOSs	3 mol L^−1^ Zn(OTF)_2_	0.1	465	70,000	80.5% at 10 A g^−1^	[74]
PTD-1	2 mol L^−1^ ZnSO_4_	0.04	188.24	4000	82.5% at 1 A g^−1^	[75]
PDAN	0.5 mol L^−1^ Zn(OTF)_2_	0.1	140	10,000	82.3% at 5 A g^−1^	[76]
QA	3 mol L^−1^ Zn(OTF)_2_	0.2	212	10,000	90.8% at 2 A g^−1^	[77]
TNP	3 mol L^−1^ ZnSO_4_	0.2	338	50,000	71.6% at 10 A g^−1^	[78]
PTZ-BDTB	30 mol kg^−1^ ZnCl_2_	0.05	202	27,000	96% at 5 A g^−1^	[79]

The design of multi-nitrogen heterocyclic donor–acceptor conjugated amphoteric organic superstructures (AOSs) has been proposed, aiming to achieve high capacity, high voltage, and long cycling life in organic electrodes [74]. AOSs feature extended π-conjugated aromatic structures and low energy for electron injection, which accelerate the charge transfer rate (Fig. 6g). The molecular structure of AOSs enables an 18e^−^ amphoteric redox electrochemical process, which involves 6e^−^ transfer in the tertiary nitrogen reaction with the OTF^−^ anion storage and 12e^−^ transfer in the imine coordination reaction with the Zn^2+^ cation storage. As a result, the cathode demonstrates a specific capacity of 465 mAh g^−1^ at 0.1 A g^−1^ and exhibit a cycle life of up to 70,000 cycles with a capacity retention of 80.5% at 10 A g^−1^. Bis(phenylamino)phenothiazin-5-ium iodide (PTD-1) combines the characteristics of both n-type and p-type redox reactions, forming a hybrid charge storage mechanism [75]. This mechanism manifests as a combination of n-type and p-type reactions in the low voltage range, while predominantly exhibiting p-type behavior at higher voltages (Fig. 6h). The bipolar charge storage mechanism of PTD-1 arises from its molecular structure, which incorporates two characteristic functional groups: the phenylamine groups on both sides and the central phenothiazine heterocycle. The redox of PTD-1 proceeds in a continuous four-step electrochemical reaction. Specifically, during the initial charging process, the phenothiazine ring of PTD-1 accepts two electrons, simultaneously releasing SO_4_^2−^ (or other ions during the initial cycles) and absorbing protons. In further charging, the two phenylamine groups lose two electrons and form cations, with SO_4_^2−^ migrating to balance the generated charge. Ultimately, PTD-1 achieves a hybrid n/p-type and p-type charge storage mechanism, completing a 4e^−^ transfer. As a result, the PTD-1 cathode exhibits an average voltage of 1.1 V, a specific capacity of 188.24 mAh g^−1^ at 40 mA g^−1^, and 73% capacity retention after 4000 cycles.

A hydrogen bonding-stabilized bipolar quinacridone (QA) features n-type carbonyl and p-type amine dual active centers, enabling 2e^−^ transfer [77]. The QA cathode exhibits a specific capacity of 212 mAh g^−1^ at 0.2 A g^−1^, with a capacity retention of 90.8% after 10,000 cycles. The multi-electron redox bipolar tetranitroporphyrin (TNP) macrocycle compound possesses multiple redox-active sites, an extended conjugated aromatic framework, and excellent redox characteristics, facilitating rapid ion insertion kinetics (Fig. 6i) [78]. The TNP charge storage mechanism involves a 10e^−^ transfer, where four -NO_2_ groups undergo two steps of 8e^−^ transfer at low potential, and two amine sites support 2e^−^ transfer at high potential. The TNP electrode delivers a specific capacity of 338 mAh g^−1^ at 0.2 A g^−1^ and 71.6% capacity retention after 50,000 cycles at 10 A g^−1^.

Additionally, the bipolar donor–acceptor (D-A) conjugated microporous polymer (PTZ-BDTB) has been synthesized, consisting of electron-withdrawing benzo[1,2-b:4,5-b']dithiophene-4,8-dione (BDTB) units and electron-donating phenothiazine (PTZ) units (Fig. 6j) [79]. PTZ-BDTB undergoes a 4e^−^ transfer reaction. Thus, the electrode exhibits a specific capacity of 202 mAh g^−1^ at 0.05 A g^−1^ and retains 109 mAh g^−1^ at 15 A g^−1^. The bipolar poly(1,8-diaminonaphthalene) (PDAN) also demonstrates excellent performance in Zn-based batteries [76]. During the charging process, the amine groups lose electrons and are converted into imine groups. This reaction primarily occurs in the voltage range between 1.15 and 0 V, representing an n-type reaction. In subsequent charging, the amine groups (–NH–) in PDAN lose electrons and are oxidized to imine cations (=NH^+^), while CF_3_SO_3_^−^ from the electrolyte migrate to the electrode surface to balance the charge. During discharge, the imine cations regain electrons, transform back into amine groups, and release CF_3_SO_3_^−^. This reaction occurs mainly in the voltage range between 1.15 and 1.6 V, representing a p-type reaction. In the n-type reaction, each repeating unit of PDAN can transfer 2e^−^, and similarly, in the p-type reaction, each repeating unit transfers another 2e^−^. As a result, the PDAN electrode experiences a total of 3e^−^ transfer per charging-discharging cycle. The PDAN cathode exhibits a specific capacity of 140 mAh g^−1^ at 0.1 A g^−1^, with virtually no capacity degradation after 1000 cycles.

Bipolar organic materials hold great potential for application in Zn-based batteries. However, high-performance bipolar organic electrodes require complex synthesis processes, which increase production costs and limit their large-scale application. The dissolution of bipolar molecules into the aqueous electrolyte remains an additional concern, particularly for those containing both n-type and p-type groups that may exhibit enhanced solubility. The molecular engineering strategies discussed above, including covalent immobilization into framework structures, incorporation of charged or hydrophobic groups, and supramolecular stabilization through π–π stacking or hydrogen bonding, are equally applicable to bipolar systems and can be synergistically combined. Future research will focus on developing more efficient bipolar organic materials, optimizing their conductivity, electrochemical performance, and stability through strategies such as molecular engineering, copolymerization, and doping. This will enhance the specific capacity and power density of Zn-based batteries, while also improving their cycle life and safety [223].

### Iodine-Based Multi-Electron Transfer Zn-Based Batteries

As discussed in the previous section, iodine is an appealing multi-electron halogen-based cathode, which possesses various valence states ranging from − 1 to + 7. However, the high solubility of polyiodide ions causes the dissolution and shuttle issue, which reduces the Coulombic efficiency and hampers the cycling stability. Therefore, designing functional cathodes that anchor polyiodide ions, and incorporating additives that interact with iodine species, are promising approaches to enable highly reversible multi-electron transfer reaction [224]. These approaches collectively address the fundamental challenges of polyiodide shuttling, the instability of high‑valence iodine intermediates (e.g., I^+^), and sluggish reaction kinetics.

#### Electrolyte Engineering

Electrolyte engineering not only forms halogen compounds with I^+^ and enables multi-electron transfer from I^−^ to I^+^, but also facilitates uniform Zn deposition and suppresses side reactions such as Zn dendrite formation. Including high-concentrated electrolytes, hydrated eutectic electrolytes, organic molecules enhance the Zn||I_2_ battery specific capacity and cycling stability by modulating the electrolyte environment, promoting multi-electron transfer, and mitigating side reactions (Table 7) [225].

**Table 7:** Comparison of electrochemical performance in four-electron-transfer Zn||I_2_ batteries

Type of additives	Additives	Electrolyte concentration	Capacity (mAh g^−1^) or Energy density (Wh kg^−1^)	Stability	References
High-concentration salt solutions	ZnCl_2_, LiCl	1 M ZnSO_4_ + 19 mol kg^−1^ ZnCl_2_ + 19 mol kg^−1^ LiCl + 8 mol kg^−1^ ACN	700 Wh kg^−1^	2 A g^−1^ for over 6000 cycles	[80]
	CuCl_2_	1 M CuSO_4_ + 0.5 M CuCl_2_	557.7 mAh g^−1^ at 1.0 A g^−1^ 251.5 mAh g^−1^ at 4.0 A g^−1^		[81]
Hydrated eutectic electrolytes	Niacinamide (NA)	NA:DMSO:Zn(ClO_4_)_2_·6H_2_O = 0.5:3:1	412 mAh g^−1^ at 0.5 A g^−1^	Zn||Zn symmetric cells with current density of 0.5 mA cm^−2^ for 2000 h	[82]
	Ethylene glycol (EG)	30 M ZnCl_2_ + 15 M ChCl_2_ in a mixed solvent of EG and H_2_O at a 1:1 volume ratio	987 mAh g^−1^_I2_ at 0.1 A g^−1^	CE remains above 91.3% after 300 cycles	[83]
	Polyethylene glycol (PEG 200)	2 M ZnCl_2_ in a mixed solvent of PEG 200 and H_2_O at a 7:3 volume ratio	528.9 mAh g^−1^	a capacity retention of 81.5% and a nearly 100% CE after 20,000 cycles	[84]
Organic molecule	Trimethylaminehydrochloride (TAH)	2 M ZnSO_4_ + 0.5 M TAH	~ 450 mAh g^−1^ at 2 A g^−1^	2 A g^−1^ for 5000 cycles	[85]
	Tetraethylammonium chloride (TEACl)	15 M ZnCl_2_ + 1 M TEACl	217.1 mAh g^−1^ at 40 A g^−1^	A CE of over 99% at -50 °C at 0.05 A g^−1^	[86]
	Tetrapropylammonium chloride (Pr_4_NCl)	3 M ZnSO_4_ + 0.1 M Pr_4_NCl	442 mAh g^−1^ at 1C	over 1100 cycles at 5C with 74% capacity retention	[87]
	ZnBr_2_	1 M ZnSO_4_ + 0.2 M ZnBr_2_	350 mAh g^−1^ at 1 A g^−1^	94.6% capacity retention after 10,000 cycles	[88]

##### High-Concentration Electrolytes

**Fig. 7:**
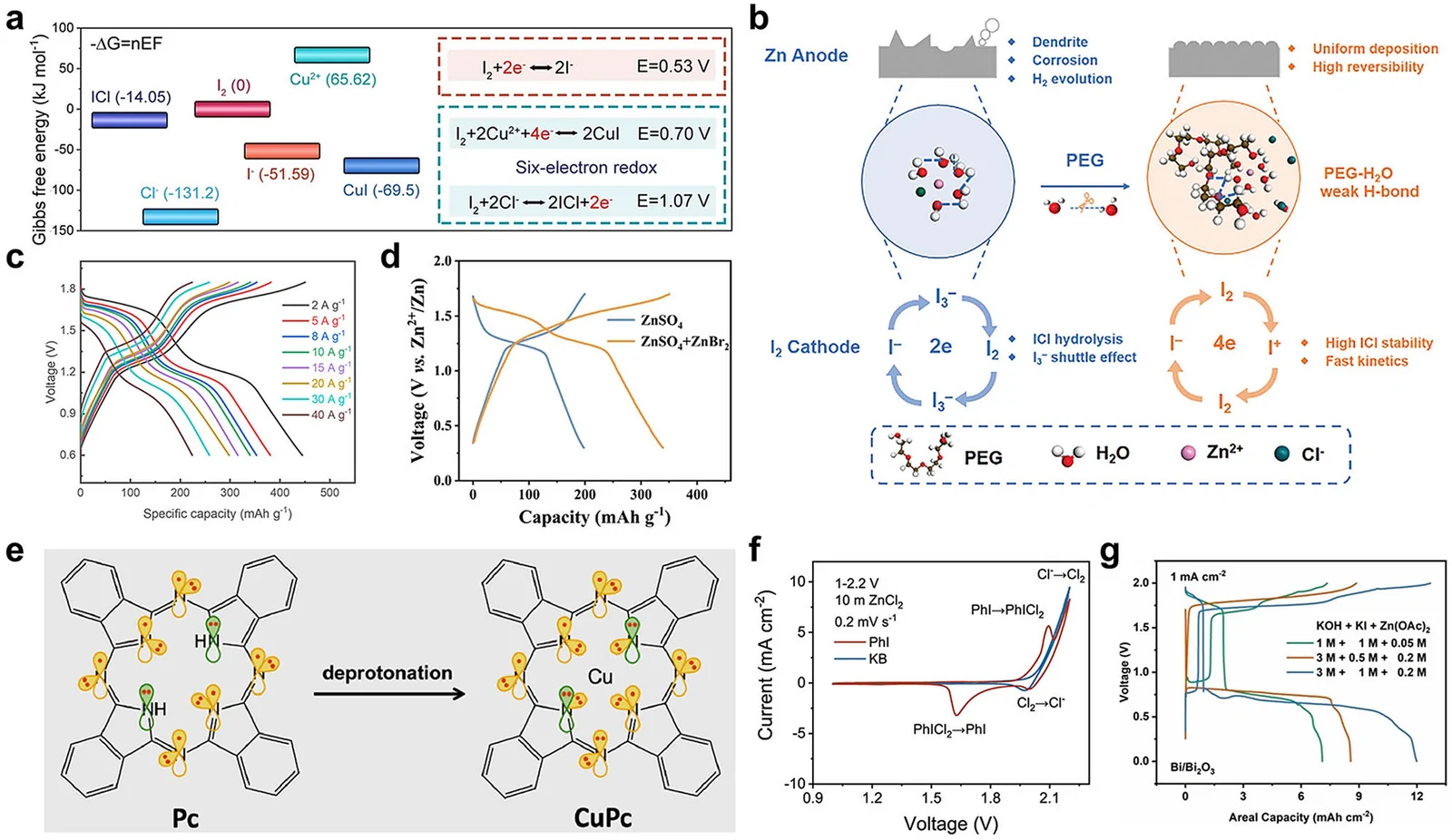
**a** Calculated Gibbs free energy and redox potentials of different I_2_-based redox couples. Reproduced with permission [81]. Copyright 2023, Wiley–VCH. **b** Schematic illustrating the role of the PEG-based hybrid electrolyte in regulating iodine redox chemistry. Reproduced with permission [84]. Copyright 2024, Wiley–VCH. **c** GCD profiles of Zn||I_2_ batteries with TAH electrolyte additive at various current densities. Reproduced with permission [85]. Copyright 2024, Wiley–VCH. **d** GCD profiles of the Ni–Fe-I electrode in ZnSO_4_ and ZnSO_4_ + ZnBr_2_ electrolytes at 1 A g^−1^. Reproduced with permission [88]. Copyright 2024, Wiley–VCH. **e** Schematic illustration of the CuPc molecule and the interfacial charge-transfer interaction with the iodine cathode. Reproduced with permission [227]. Copyright 2024, Elsevier. **f** CV curves of the PhI and inert KB electrodes in a ZnCl_2_ electrolyte at 0.2 mV s^−1^. Reproduced with permission [228]. Copyright 2024, American Chemical Society. **g** GCD profiles of the Super P or Bi/Bi_2_O_3_ electrodes in KOH or KOH + KI electrolytes. Reproduced with permission [229]. Copyright 2024, Wiley–VCH

High-concentration electrolytes help regulate ionic interactions and the ionic strength of the solution, facilitating stepwise redox reactions of iodine species. This enables multi-electron transfer and enhances the capacity of Zn||I_2_ batteries [226]. For instance, Cl^−^ anions can suppress water activity and mitigate the hydrolysis of I^+^ species. More importantly, free Cl^−^ participates in the redox process by forming inter-halogen compounds (e.g., ICl), thereby activating the I_2_/I^+^ couple and enhancing kinetics. This strategy has enabled Zn||I_2_ batteries with a specific energy up to 750 Wh kg^−1^ and a cycle life exceeding 6000 cycles [80].A synergistic 6e^−^ redox process has been realized by coupling redox-active Cu^2+^ ions with Cl^−^ anions. Cu^2+^ facilitates a low-potential reaction to form CuI, while Cl^−^ activates a high-potential I_2_/ICl redox couple, collectively achieving high capacity and stable cycling (Fig. 7a) [81]. Consequently, the developed Zn||I_2_ battery operates via this 6e^−^ redox mechanism, which confers a high discharge potential, superior capacity, excellent rate capability, and stable cycling performance. The 6e^−^ redox is compatible with various metal anodes in aqueous electrolytes, such as Zn, Mn, and Fe, facilitating the construction of metal-I_2_ hybrid batteries with enhanced capacities and energy densities. Notably, the Zn||I_2_ battery achieves a high capacity of 557.7 mAh g^−1^ at 1.0 A g^−1^ and retains 251.5 mAh g^−1^ at 4.0 A g^−1^. Although Cl^−^ effectively stabilizes I^+^ species, some interfacial side effects remain unavoidable. Moreover, this high-concentration approach involves a classic trade-off: While effectively suppressing polyiodide dissolution, the concomitant increase in viscosity and decrease in ionic conductivity (often dropping to < 20 mS cm^−1^ in ultra-concentrated electrolytes) can slow Zn^2+^ transport and limit the rate capability, which is especially detrimental for multi-electron reactions demanding high ion flux. Consequently, optimizing the salt concentration to balance shuttle suppression against adequate kinetics or developing alternative strategies like ion-sieving interfacial layers is essential.

##### Hydrated Eutectic Electrolytes

Hydrated eutectic electrolytes are mixtures composed of two or more salts or solvents, which form eutectic system at specific ratios. This eutectic system effectively regulates the solubility and chemical reaction pathway of iodine, which mitigates the shuttle effect. Furthermore, hydrated eutectic electrolytes enhance the electrochemical reaction efficiency between Zn and iodine by optimizing the ionic environment and increasing conductivity. A ternary eutectic electrolyte composed of dimethyl sulfoxide (DMSO), niacinamide (NA), and Zn perchlorate (Zn(ClO_4_)_2_) promotes the formation of stable [I(NA)_2_]^+^ complexes, stabilizes I^+^ species, and triggers a reversible 4e^−^ transfer process, resulting in a stable capacity of 412 mAh g^−1^ [82]. Similarly, introducing ethylene glycol (EG) as a co-solvent disrupts the water hydrogen‑bonding network, alters the Zn^2+^ solvation structure, and improves the reversibility of I^−^/I^0^/I^+^ and Cl^−^/Cl^0^ redox, leading to a remarkable capacity of 987 mAh g^−1^ and a specific energy of 1278 Wh kg^−1^ [83]. Polyethylene glycol (PEG 200) functions analogously as a co-solvent in a 2 M ZnCl_2_ aqueous solution, which addresses the I^+^ hydrolysis challenges and Zn plating instability (Fig. 7b) [84]. The PEG 200 reduces water activity to restrain I^+^ hydrolysis and facilitates I^−^/I^0^/I^+^ 4-electron redox kinetics, which enables stable operation across a wide-temperature range (40 to − 40 °C).

However, the synthesis of hydrated eutectic electrolytes is intricate, which necessitates precise control over the solvent-to-solute ratio and the conditions for eutectic formation. Moreover, the interfacial compatibility of hydrated eutectic electrolytes with certain electrode materials is suboptimal, potentially leading to increased interfacial resistance and detrimental effects on the cycling stability and power output.

##### Organic Molecules

There is a wide variety of organic molecule additives, whose structures can be tailored through chemical synthesis, offering greater design flexibility. Organic molecules can facilitate multi-step redox reactions of iodine by providing additional electron transfer sites or by modulating the solvent environment of the electrolyte. Trimethylamine hydrochloride (TAH), with a bidentate coordination structure, can adsorb onto the Zn anode surface, reducing the desolvation energy barrier of Zn^2+^ ions and promoting uniform deposition, thereby enhancing Zn utilization [85]. The Zn anode demonstrates stable cycling for 100 h with the Zn utilization rate of 97% and an exceptional areal capacity of 57 mAh cm^−2^. On the cathode side, TAH (containing amine groups and Cl^−^) establishes a distinctive bidentate coordination with I^+^, enabling an efficient 4e^−^ high-valence redox process. This enhances the specific capacity to nearly 450 mAh g^−1^ at 2 A g^−1^ and maintains over 200 mAh g^−1^ even at 40 A g^−1^ (Fig. 7c). Hydrophobic tetraethylammonium cations (TEA⁺) serve as ion sieves to dynamically modulate the electrode/electrolyte interfacial environment [86]. This approach creates a water-poor but chlorine-rich interfacial environment at the electrode surface. These modifications prevent the decomposition of ICl and enhance the Zn plating reversibility and the 4e^−^ iodine redox chemistry. The modified Zn||I_2_ battery exhibited excellent high-rate capability (217.1 mAh g^−1^ at 40 A g^−1^) at room temperature and demonstrated stable cycling at − 50 °C. Systematic studies on quaternary ammonium cations of varying chain lengths (tetramethylammonium, tetraethylammonium, tetrapropylammonium, tetrabutylammonium, tetraamylammonium, and tetraheptylammonium chloride) have shown their efficacy in forming stable complexes with polyhalides like ICl_2_^−^, effectively mitigating the hydrolysis and facilitating the 4e^−^ I^−^/I^0^/I^+^ redox process [87]. Additionally, Br^−^ anions can activate and stabilize I^+^ via interhalogen formation. When coupled with a suitable host material like Ni–Fe-I layered double hydroxide (LDH) nanoflowers, the I^−^/I^+^ reactions can be confined within the interlayers, further enhancing the cycling stability. This approach yields a specific capacity of 350 mAh g^−1^ at 1 A g^−1^ (Fig. 7d) [88].

While the implementation of special electrolytes, such as highly concentrated water in salt electrolytes, deep eutectic solvents or those heavily formulated with organic cosolvents, effectively widens the electrochemical stability window and suppresses parasitic reactions to enable high voltage operation, their practical application boundaries must be rigorously acknowledged. These specialized formulations often introduce severe trade-offs: The high concentration of salts drastically increases the overall cost and electrolyte viscosity, which inevitably compromises ionic conductivity and severely degrades low-temperature performance. Furthermore, the poor wettability of these viscous electrolytes with thick, commercial-level electrodes remains a formidable barrier to scale-up. Therefore, while these electrolytes yield impressive laboratory-scale metrics, transitioning them to commercially viable, large-scale energy storage systems requires solving these inherent physiochemical and economic bottlenecks.

#### Cathode Material Design

The development of suitable cathode host material is critical for confining active iodine species and providing catalytic sites. Carbon nanomaterials, MOFs, and layered materials have been widely explored for their high surface area and tunable porosity [230]. Chemical modification of these hosts with iodine‑philic groups (e.g., amine, quaternary ammonium) can enhance iodine adsorption and stabilize intermediates. Heterostructured hosts with charge transfer capabilities offer advanced functionality. For example, copper phthalocyanine (CuPc) incorporated into a carbon framework acts as an electron‑outflowing host (Fig. 7e) [227]. Its charge‑transfer interaction with I^+^ stabilizes these otherwise unstable species, enabling the reversible I^+^/I_5_^−^ conversion and resulting in a specific capacity of 229 mAh g^−1^ at 0.5 A g^−1^. Single‑atom catalysts represent another frontier. Fe single atoms on N‑doped carbon (FeSA-NC) catalyze iodine redox through d‑p orbital hybridization, suppressing I_3_^−^ formation and ICl hydrolysis, which leads to a high capacity of 376.4 mAh g^−1^ and stability over 10,000 cycles [231].

Beyond the elemental iodine, organoiodine compounds open new redox avenues. The reversible single-step transition from iodobenzene (PhI) to PhICl₂ (I^+^ to I^3+^) in aqueous ZnCl_2_ electrolyte has been demonstrated, delivering a high capacity of 422 mAh g^−1^ (based on iodine) and stable cycling over 400 cycles, bridging hypervalent iodine chemistry with battery technology (Fig. 7f) [228]. For the ambitious 12e^−^ I^−^/IO_3_^−^ couple, the large voltage hysteresis poses a major challenge. Employing a Bi/Bi_2_O_3_ redox mediator in alkaline electrolyte has been shown to catalyze IO_3_^−^ reduction, slashing the voltage gap from 1.6 to 1 V and significantly improving the round-trip energy efficiency and rate capability (Fig. 7g) [229].

Cathode materials can be tailored with various structures and surface modifications to fulfill multiple functions, such as providing catalytic active sites and anchoring iodine species. These modifications effectively immobilize iodine species, reduce their dissolution and shuttle effects, and facilitate multi-step reactions for multi-electron transfer. However, such cathode materials often require complex synthesis and design processes, involving costly procedures and higher production expenses. The lengthy development cycle from conceptual design to practical application further limits the potential for rapid commercialization.

It is worth noting that while this section primarily focuses on iodine-based cathodes, other members of the halogen group, such as bromine (Br), also possess theoretical potential for multi-electron transfer (e.g., Br^−^/Br_2_/Br^+^ transitions). However, translating these theoretical pathways into practical aqueous zinc batteries remains exceptionally challenging. The highly positive redox potentials required to trigger the multi-electron transfer of Br or Cl typically exceed the thermodynamic stability window of conventional aqueous electrolytes, inevitably triggering severe oxygen evolution reactions (OER). Furthermore, the extreme corrosiveness of these high-valence halogen species imposes stringent requirements on current collectors and cell packaging. Therefore, until breakthrough electrolyte formulations (such as highly concentrated water-in-salt or deep eutectic solvents) and corrosion-resistant components are fully developed, iodine will continue to dominate the research landscape for multi-electron halogen zinc batteries.

### S-Based Multi-Electron Transfer Zn-Based Batteries

Zn||S batteries integrate the cost and capacity advantages of both Zn anode and S cathode, which are considered as promising high-energy battery systems. Although the aqueous zinc-sulfur system offers inherent safety advantages, it continues to face considerable challenges in interfacial side reactions and sluggish reaction kinetics [232]. Valuable insights can be drawn from non-aqueous lithium-sulfur batteries, where the critical role of solvation chemistry in governing interfacial stability has been increasingly recognized [233]. For example, recent studies have shown that salt anions can compete with solvent molecules to occupy the inner solvation shell of polysulfide intermediates, and that promoting anion coordination while reducing weak solvent coordination can effectively lower the charge-transfer barriers that limit reaction kinetics [234]. This concept is transferable to aqueous Zn||S systems, where electrolyte formulations that favor an anion-dominated Zn^2+^ solvation sheath may similarly reduce the activation energy of the sluggish solid–solid ZnS/S conversion. In addition, studies on thick sulfur cathodes in Li||S batteries have revealed that preferential solid deposition on the cathode surface can obstruct mass transfer pathways and trigger premature discharge termination [235]. This finding is relevant to aqueous Zn||S batteries, where the insulating ZnS discharge product accumulates on the cathode surface and similarly impedes ion transport. Strategies demonstrated in Li||S pouch cells that employ macroporous cathode architectures to mitigate irreversible volume expansion and maintain electrolyte infiltration can be adapted to extend the cycling life of aqueous Zn||S batteries [236]. Taken together, these cross-system analogies indicate that solvation chemistry and cathode structural design are two key factors governing interfacial stability and reaction reversibility in both organic and aqueous sulfur-based batteries. In this section, we highlight some representative strategies to overcome these issues in Zn||S batteries, including the electrolyte optimization and cathode catalyst engineering. Electrolyte optimization aims to improve ionic conductivity and suppress side reactions involving Zn^2+^ and sulfides by adjusting concentration, selecting appropriate solvents, and incorporating functional additives, thereby improving capacity and cycling stability. Concurrently, cathode catalyst engineering involves selecting catalytic materials to enhance sulfur capacity utilization and mitigate the dissolution of polysulfide intermediates, which improves charge–discharge efficiency and long-term stability. These strategies collectively provide valuable insights for the advancement of Zn||S batteries.

#### Additives

The introduction of additives such as I_2_, ZnI_2_, eutectic electrolytes, and organic molecular can significantly enhance the electrochemical performance of the Zn||S batteries by suppressing the polysulfide shuttle effect, improving stability, accelerating the reaction kinetics, and increasing the utilization efficiency (Table 8) [93].

**Table 8:** Performance comparison of Zn||S batteries with different additives

Additive	Electrolyte	Battery structure	Energy density (Wh kg^−1^)	Specific capacity	Current density (A g^−1^)	Cycle life	Capacitance retention	References
ZnI_2_	3 mol L^−1^ ZnSO_4_	Zn||KB/S	742 (S)	1580	0.5	550	100% at 5 A g^−1^	[89]
I_2_ + Ethylene glycol (EG)	1 mol L^−1^ Zn(OAc)_2_	Zn||S@VC	605	1210	0.075 mA g^−1^	250	91% at 0.075 A g^−1^	[90]
Succinonitrile (SN)	8 mol L^−1^ Zn(ClO_4_)_2_	Zn||S	527 Wh kg_S_^−1^	304	0.1	500	85.7% at 2 A g^−1^	[91]
Me_3_PhNI	1 mol L^−1^ Zn(OAc)_2_	Zn||KB/S	1327	1659	0.1	100	59% at 3 A g^−1^	[92]
ZnI_2_ + Thiourea (TU)	2 mol L^−1^ ZnSO_4_	Zn||ZnS		1478	0.1	300	53.3% at 2 A g^−1^	[93]
Thiourea (TU)	2 mol L^−1^ ZnSO_4_	Zn||S@KB	803.3	1785	1	300	67% at 5 A g^−1^	[94]
Ethylene glycol (EG) + ZnI_2_	2 mol L^−1^ Zn(OTF)_2_	Zn||S@NPC	730	1435	0.1	250	70% at 3 A g^−1^	[95]
PEG-400	1 mol L^−1^ Zn(OAc)_2_	Zn||S@CNTs		1116	0.1	300	81.7% at 1 A g^−1^	[96]
ZnI_2_	2 mol L^−1^ Zn(OTF)_2_	Zn||S@AC	582	1453	0.1	300	72% at 5 A g^−1^	[97]
Ethylene glycol (EG)	1 mol L^−1^ Zn(OAc)_2_ + 1 mol L^−1^ ZnBF	Zn||AC-S	184.8	675	0.25	800	98% at 3 A g^−1^	[98]
I_2_	1 mol L^−1^ Zn(OAc)_2_	Zn||S@CNTs	502	1105	0.1	225	44.8% at 1 A g^−1^	[99]
LiCl + Acetonitrile (ACN)	0.5 mol L^−1^ ZnCl_2_	Zn||S@C	259	846	0.5	50	4.54% at 0.5 A g^−1^	[100]
NMP + KI	1 mol L^−1^ Zn(OTF)_2_	Zn||N-CNTs/S		1549	0.5	300	77.5% at 5 A g^−1^	[90]
Acetonitrile (ACN) + I_2_	3 mol L^−1^ Zn(OTF)_2_	Zn||S@PECNTs		1370	0.1	25	66.8% at 0.1 A g^−1^	[93]
ZnI_2_	3 mol L^−1^ ZnSO_4_	ZnS||S@S,N-CNF	925	1481	0.5	100	42.4% at 1 A g^−1^	[101]

##### I_2_ as an Additive

**Fig. 8:**
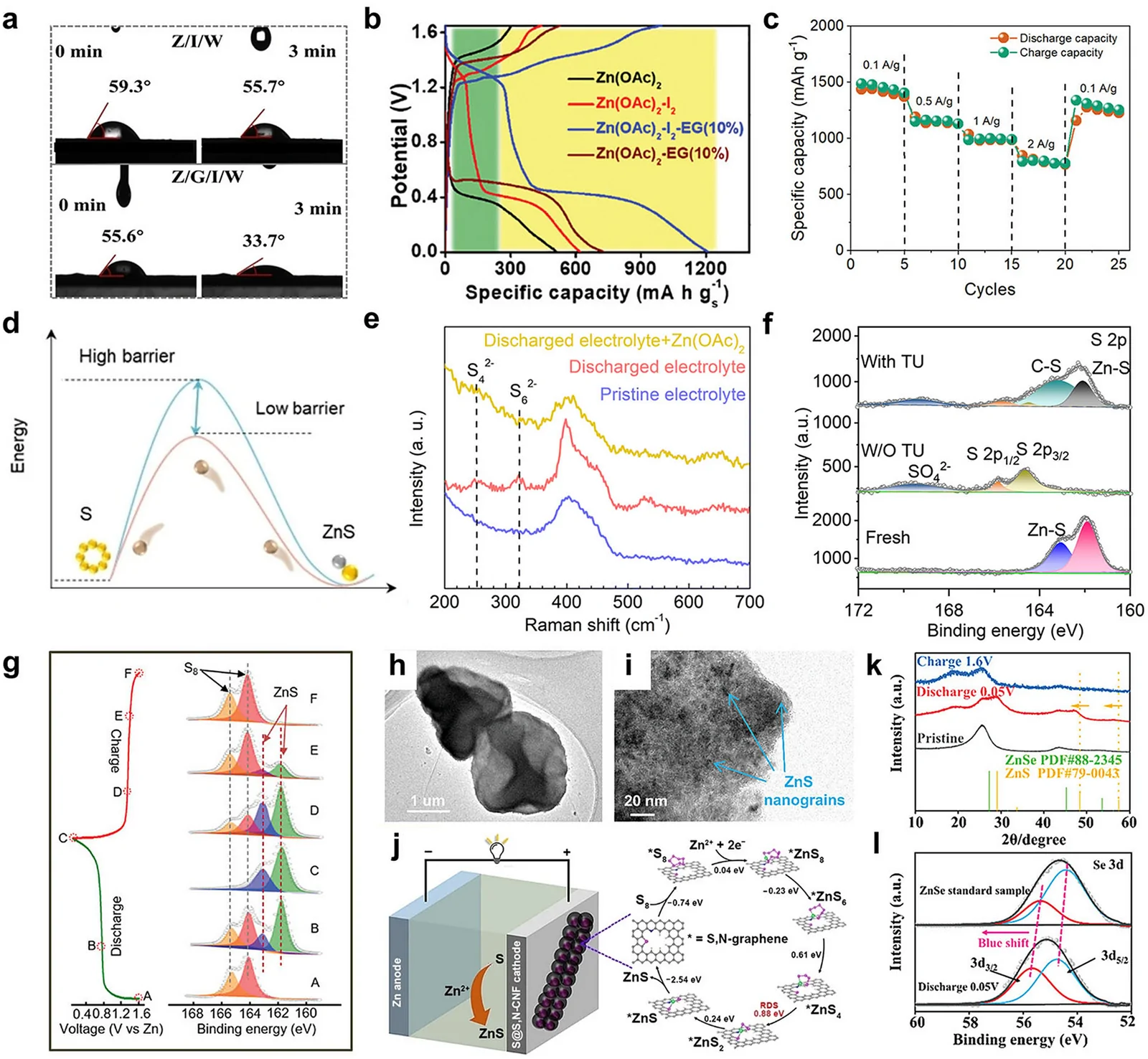
**a** Water contact angle profiles of Z/I/W and Z/G/I/W on the S cathode measured within 3 min. Reproduced with permission [237]. Copyright 2022, Wiley–VCH. **b** GCD profiles of Zn||S battery in various electrolytes. Reproduced with permission [90]. Copyright 2024, Wiley–VCH. **c** Rate performance of Zn/50-S@NPC in the hybrid electrolyte. Reproduced with permission [95]. Copyright 2023, Wiley–VCH. **d** Impact of electrolyte composition on the reaction energy barriers of sulfur species. Reproduced with permission [239]. Copyright 2024, Wiley–VCH. **e** Raman spectra of the electrolyte at different stages. Reproduced with permission [92]. Copyright 2023, Royal Society of Chemistry. **f** Comparative S 2*p* XPS analysis of the pristine and first-charged ZnS cathode. Reproduced with permission [94]. Copyright 2024, Elsevier. **g** Evolution of sulfur species probed by Ex Situ XPS on the S@FeNC/NC/CC electrode during cycling. Reproduced with permission [240]. Copyright 2023, Wiley–VCH. **h, i** TEM images of the MN-ZnS/C-H. Reproduced with permission [241]. Copyright 2024, Wiley–VCH. **j** Schematic of the SRR on the S, N‑CNF electrocatalyst in a Zn||S battery. Reproduced with permission [103]. Copyright 2024, Wiley–VCH. **k** Phase evolution monitored by ex-situ XRD. **l** Chemical state of Se at discharged 0.05 V probed by XPS, compared with standard ZnSe. Reproduced with permission [102]. Copyright 2024, Wiley–VCH

Incorporating I_2_ into the electrolyte enhances sulfur conversion kinetics, facilitates Zn^2+^ transport, and suppresses disproportionation during sulfur conversion. A “cocktail-optimized” electrolyte prepared with tetraethylene glycol dimethyl ether (G4), water, Zn(OTF)_2_, and I_2_ improves the electrolyte wettability (Fig. 8a) and activates an efficient I_3_^−^/I^−^ redox catalyst, reducing polarization and lowering activation energy [237]. This synergy also fosters a stabilizing organic–inorganic interface on the Zn anode. The proposed reactions involve I_2_ facilitating the reversible conversion between S and ZnS. Zn||Zn symmetric cells with this electrolyte exhibited a cycling life of 1800 h at 5 mA cm^−2^ and 5 mAh cm^−2^. The corresponding Zn||S battery delivered a specific capacity of 1140 mAh g^−1^ at 0.5 A g^−1^. In a 10% ethylene glycol system with Zn(OAc)_2_ and I_2_, ethylene glycol suppresses hydrogen evolution and forms a protective interface on Zn, while I_2_ accelerates the reaction kinetics [90]. The assembled battery showed a specific capacity of 1210 mAh g^−1^ at 0.1C (Fig. 8b) and long-term cycling stability with 64.5% capacity retention after 3000 cycles at 1C. I_2_ addition also enabled flexible fiber Zn||S batteries with a wide operating temperature range from − 25 to 25 °C, a specific energy of 184.8 Wh kg^−1^, and a 200% strain in tensile testing [98]. Adding 0.05 wt% I_2_ to a 1 mol L^−1^ Zn(CH_3_COO)_2_ (pH = 6.5) electrolyte improved the cycling kinetics, reduced low-voltage hysteresis, and minimized voltage gaps [99]. The Zn||S battery achieved a specific capacity of 1105 mAh g^−1^ and specific energy of 502 Wh kg^−1^ at 100 mA g^−1^. Similarly, trace amounts of I_2_ in the acetonitrile/water co-solvent system enhanced wettability, reduced charge transfer resistance, suppressed polysulfide dissolution, and achieved a capacity of 1370 mAh g^−1^ at 0.1 A g^−1^ [238].

##### ZnI_2_ as an Additive

To address the slow kinetics and poor reversibility of sulfur, ZnI_2_ has been proposed as additives to promote the complex formation with S_8_ and facilitate the reaction with Zn^2+^ to form ZnS. During charging, I^−^ assists in converting ZnS back to sulfur. Iodide ions act as catalysts, promoting reversible sulfur conversion. Without them, insulating ZnS accumulates, hindering reactions. ZnI_2_ introduction induces crystal distortion at the ZnS surface, weakens the Zn–S bond, and accelerates its electrochemical decomposition [89]. Batteries with ZnI_2_ additives demonstrated a specific capacity of 1580 mAh g^−1^ at 0.5 A g^−1^ and excellent cycling stability. Synergistic use of N-methylpyrrolidone (NMP) and KI further improves kinetics. NMP modifies the Zn^2+^ solvation sheath, promotes uniform Zn deposition, and suppresses dendrites, while I^−^/I_3_^−^ complexes participate in sulfur conversion, yielding a capacity of 1549 mAh g^−1^ at 0.5 A g^−1^ [242]. Adding ZnI_2_ to an ethylene glycol/water mixture also accelerates the oxidation of ZnS back to polysulfides and sulfur, achieving 1435 mAh g^−1^ at 0.1 A g^−1^ (Fig. 8c) and 70% capacity retention after 250 cycles at 3 A g^−1^ [95].

##### Eutectic Electrolyte

Eutectic electrolytes can significantly enhance the electrochemical performance of Zn||S batteries through multiple effects, including improved ionic conductivity, enhanced electrode interface stability, suppression of polysulfide side reactions, and reinforced thermal stability. A high-concentration Zn(ClO_4_)_2_ (8 M) electrolyte was employed to facilitate multivalent sulfur conversion (S^2−^ to S^0^ to S^4+^), with spectroscopic and theoretical analyses confirming the pathway [91]. The fabricated Zn||S battery exhibited a high operating voltage of 1.54 V, a capacity of 1284 mAh g^−1^ after 500 cycles at 2 A g^−1^, and a specific energy of 527 Wh kg^−1^. Another eutectic solvent based on urea and choline chloride with various zinc salts (ZnSO_4_, ZnCl_2_, Zn(NO_3_)_2_, and Zn(OAc)_2_) suppressed hydrogen evolution and dendrite formation, enabling stable Zn||Zn batteries with 3920 h’ cycling and Zn||S batteries with a high capacity of 846 mAh g^−1^ at 0.5 A g^−1^ [100]. Additionally, adding propylene glycol monomethyl ether (PM) to ZnI_2_ as a co-solvent provides electrons for the redox reaction of I^−^/I_3_^−^, improving the kinetics of the sulfur cathode (Fig. 8d) [239]. The hydroxyl (-OH) and ether (C–O–C) groups in PM also effectively modulate the solvation structure of Zn^2+^ and the hydrogen bonding network, accelerating Zn^2+^ transport and suppressing side reactions during sulfur conversion. When the volume fraction of PM is 60% in the electrolyte, the Zn||S battery achieves a specific capacity of 1456 mAh g^−1^ and a specific energy of 471.8 Wh kg^−1^ at 0.2 A g^−1^.

##### Organic Molecule Additives

The incorporation of organic additives also effectively improves the solid–solid conversion kinetics between sulfur and zinc sulfide. Furthermore, these additives play a crucial role in enhancing the Zn anode interface stability, suppressing dendrite growth, and improving ionic conductivity, which are key to enhancing the overall electrochemical performance of Zn||S batteries. To address the issues of slow solid-to-solid reaction kinetics between the sulfur cathode and discharge product ZnS, and poor redox kinetics of the sulfur cathode, quaternary ammonium iodide (R_4_N^+^I^−^) was introduced as a functional additive into the electrolyte [92]. R_4_N^+^ cations induce the formation of soluble polysulfide intermediates (Fig. 8e), converting the reaction to a solid–liquid–solid pathway, thereby accelerating kinetics. During charging, the I^−^/I_3_^−^ redox mediator catalyzes ZnS oxidation. The lower solubility of R_4_N^+^I_3_^−^ compared to Zn(I_3_)_2_ helps mitigate polysulfide shuttling. The battery delivered 1659 mAh g^−1^ at 0.1 A g^−1^ and retains 851 mAh g^−1^ at 5 A g^−1^. Thiourea iodide serves as a dual-function redox mediator, where I⁻ lowers overpotentials and thiourea regulates Zn deposition and reduces corrosion [45]. A capacity of 1410 mAh g^−1^ at 0.1 A g^−1^ was achieved. A trace thiourea additive in ZnSO_4_ forms a metal–organic complex protective layer on the cathode (“raincoat” strategy), blocking water contact while allowing Zn^2+^ transfer, achieving 1478 mAh g^−1^ at 0.1 A g^−1^ [243]. Thiourea also improves the reversibility of the ZnS/S conversion, with intermediates weakening the Zn–S bond and preventing SO_4_^2−^ formation (Fig. 8f), leading to a decay rate of only 0.11% per cycle at 5 A g^−1^ [94]. The addition of PEG-400 to Zn(OAc)_2_ electrolyte enhances the solvation structure of Zn^2+^, resulting in the formation of more uniform and smaller ZnS, which benefits the reversible conversion from ZnS to sulfur [96]. This improvement effectively increases the reversibility of the S cathode. Zn||Zn symmetric batteries using this PEG-based electrolyte demonstrate stable cycling for over 480 h, while the Zn||S batteries using this electrolyte exhibit a low voltage hysteresis of 0.47 V and a high specific capacity of 1116 mAh g^−1^ at 0.1 A g^−1^. A hybrid electrolyte with dimethylacetamide (DMA) and ZnI_2_ features DMA participating in the Zn^2+^ solvation shell, restructuring it to suppress side reactions and promote rapid sulfur conversion, resulting in a capacity of 1453 mAh g^−1^ at 0.1 A g^−1^ and 72% retention after 300 cycles at 5 A g^−1^ [97].

Despite their advantages in improving capacity, rate performance, and cycling stability, these additives present certain drawbacks. I_2_ and ZnI_2_ may induce side reactions with the Zn anode, eutectic electrolyte design is complex, and organic additives can reduce electrolyte conductivity. Therefore, the selection of appropriate additives based on application requirements and cost, and the exploration of synergistic combinations, are crucial for further optimization.

#### Sulfur Cathode

The cathode catalyst support enhances the redox reactions of sulfur, reduces side reactions, and improves the rate performance and cycling stability of Zn||S batteries (Table 9) [101]. By injecting single Fe atoms into nitrogen-doped porous carbon to prepare Fe-N_4_ structured catalysts, the complete conversion of sulfur is realized, and the energy barrier for ZnS decomposition is minimized, thereby significantly improving the cycling stability [240]. The Fe sites favor strong adsorption toward sulfur and polysulfide intermediates, ensuring almost complete conversion of sulfur to ZnS during discharge. In the subsequent charging process, the Fe sites reduce the activation energy of ZnS, facilitating the conversion of ZnS to sulfur (Fig. 8g). Consequently, Zn||S batteries exhibit a discharge capacity of 1143 mAh g^−1^. Biomass-derived carbon with single-atom Co (MMPC-Co) serves as a sulfur host accelerates charge transfer and sulfur reduction kinetics, enabling a rapid solid–solid transition and inhibiting polysulfide and SO_4_^2−^ formation [106]. The abundant porous structure not only physically reduces sulfur loss but also accelerates Zn^2+^ transport. Moreover, the macropores of MMPC-Co mitigate the stress effects caused by the electrode volume expansion, enhancing the cycling stability. Zn||S batteries deliver a specific capacity of 507.21 mAh g^−1^ at 5 A g^−1^ and retain 729.96 mAh g^−1^ after 500 cycles at 4 A g^−1^. A composite of CoO nanocrystals anchored on N-doped porous carbon promotes sulfur conversion and lowers the ZnS activation barrier, achieving 1172 mAh g^−1^ at 0.2 A g^−1^ with 71.6% capacity retention after 500 cycles [65]. Using coal tar pitch as a precursor and K_2_CO_3_ as an activator, three-dimensional amorphous carbon was prepared [119]. In addition, industrial-grade sulfur powder was used, with sulfur content ranging from 60 to 80 wt%, which is higher than the typical sulfur content (40 to 60 wt%) in most Zn||S batteries. This could reduce battery cost and increase the cell-level energy density. The S electrode delivers a specific capacity of 633.5 mAh g^−1^ at 0.5 A g^−1^ and a specific energy of 297.5 Wh kg^−1^.

**Table 9:** Performance comparison of Zn||S batteries with different cathode catalysts

Cathode catalysts	Electrolyte	Energy density	Specific capacity	Current density (A g^−1^)	Cycle life	Capacitance retention	References
Se_0.06_S_0.94_@HCS	2 mol L^−1^ Zn(OTF)_2_		1490	0.5	500	72.8% at 4 A g^−1^	[102]
S@S,N-CNF	3 mol L^−1^ ZnSO_4_	925 Wh kg^−1^	1481	0.5	100	42.4% at 1 A g^−1^	[103]
Te_1_S_7_/C	1 mol L^−1^ ZnSO_4_	934.6 Wh kg^−1^	1335	0.1	25	73.9% at 0.2 A g^−1^	[104]
S@Fe-PANi	2 mol L^−1^ ZnSO_4_	720 Wh kgs^−1^	1205	0.2	500	85% at 0.2 A g^−1^	[105]
S@MMPC-Co	2 mol L^−1^ Zn(OTF)_2_		1445	1	500	729.96 mAh g^−1^ at 4 A g^−1^	[106]
ZnS_0.93_Se_0.07_	2 mol L^−1^ ZnSO_4_		552	0.1	200	52% at 2 A g^−1^	[107]

##### Nanosizing of Sulfur

The preparation of nano-sized sulfur not only increases the specific surface area but also shortens the ion diffusion path within the S electrode, allowing Zn^2+^ to react efficiently with sulfur. This effect improves the charge/discharge rate and efficiency of Zn||S batteries. Embedding ZnS nanoparticles (< 10 nm) within a carbon microsphere scaffold enhances ion transport and reaction kinetics (Fig. 8h, i), providing a long lifespan of 210 cycles with 93.2% capacity retention at 2 A g^−1^ [241]. Confining sulfur nanoparticles within S, N co-doped carbon nanofibers via a yolk–shell structure synergistically modulates polysulfide adsorption and lowers the energy barrier for conversion, achieving 1481 mAh g^−1^ and 925 Wh kg^−1^ at 0.5 A g^−1^ (Fig. 8j) [103]. Doping sulfur nanoparticles with the Fe(CN)_6_^4−^ redox couple and polymerizing polyaniline creates a composite cathode, where the Fe(CN)_6_^4−^/Fe(CN)_6_^3−^ redox couple activates sulfur and accelerates kinetics, yielding 1205 mAh g^−1^ at 0.2 A g^−1^ and a specific energy of 720 Wh kg^−1^ [105]. The fabricated flexible all-solid-state Zn||S battery demonstrates an energy density of 375 Wh kg^−1^.

##### Heteroatom Doping (Se, Te, and I)

Doping sulfur with Se, Te, or I can significantly enhance its electric conductivity, reduce internal resistance, and consequently improve the rate performance and power density of Zn||S batteries. Introducing Se-S bonds into sulfur establishes an internal electron transfer path from Se to S, thereby accelerating the redox reaction of sulfur [102]. Conductive Se acts as an electron donor, while electron-deficient Se sites exhibit affinity for I^−^, boosting catalytic efficiency. The cathode undergoes reversible conversion between Se_0.06_S_0.94_ and ZnSe_x_S_1-x_ (Fig. 8k, l), preventing soluble Se intermediate dissolution. A capacity of 1490 mAh g^−1^ at 0.5 A g^−1^, 1038 mAh g^−1^ at 4 A g^−1^, and a capacity retention rate of 70.8% after 500 cycles were achieved. Doping Se into ZnS (ZnS_0.93_Se_0.07_) also mitigates the issue of electrode volume expansion during cycling [107]. Se doping alters the band structure of ZnS, reducing its bandgap from 1.86 eV to 1.40 eV, which enhances the intrinsic conductivity of ZnS and accelerates the kinetics of the cathode reaction. The resulting Zn||S battery demonstrates a specific capacity of 1116 mAh g^−1^ at 0.1 A g^−1^. Doping sulfur with Te to prepare a Te_1_S_7_ cathode reduces voltage polarization, improves sulfur utilization, and contributes additional capacity via ZnTe formation, delivering 1335.0 mAh g^−1^ at 0.1 A g^−1^ [104].

Using catalysts as sulfur carriers in Zn||S batteries can lower the activation energy barrier for sulfur redox reactions, thereby increasing the reaction rate and enhancing the power density. Furthermore, catalysts improve the interfacial reactions for cathode, help suppress the dissolution of polysulfides, and enhance the cycling stability of the Zn||S battery. However, selecting the appropriate catalyst material and ensuring its stability over long-term cycling is a challenge. Some catalysts may undergo side reactions in Zn||S batteries, adversely affecting the battery lifespan and efficiency. Preparing sulfur as nanoparticles can shorten the diffusion path for Zn^2+^, thus improving the rate performance and efficiency of Zn||S batteries while reducing internal resistance. However, nano-sized sulfur may face issues such as particle aggregation or structural degradation during cycling, which can lead to insufficient cycle life. Doping elements such as Se, Te, and I can enhance conductivity and reactivity. However, doped elements may react with the electrolyte or other components during prolonged cycling, leading to the dissolution or deactivation of the dopants and thus degrading the performance Zn||S batteries. While these three strategies can each improve the performance to some extent, each also has its limitations. To achieve further performance enhancement, it is feasible to combine these approaches to overcome their respective challenges in practical applications.

## Systematic Analysis of Failure Modes and Degradation Mechanisms

While the pursuit of multi-electron transfer strategies has yielded remarkable capacities, a critical evaluation reveals that these very mechanisms often trigger severe and complex degradation pathways. To provide a balanced perspective, it is imperative to systematically analyze the major failure modes that currently hinder the long-term cycling stability of these advanced cathodes.

### Severe Active Material Dissolution and the Shuttle Effect

The most pervasive failure mode across multi-electron systems is the generation of highly soluble intermediate species. Unlike solid-state single-electron intercalation, multi-electron pathways frequently cross the solid–liquid phase boundary. For inorganic materials like MnO_2_, the two-electron reduction inevitably generates highly soluble Mn^2+^. If not immediately re-oxidized or confined, these ions diffuse into the bulk electrolyte, causing catastrophic capacity decay. Similarly, in elemental and organic cathodes (e.g., iodine, sulfur and small molecule organics), the multi-electron processes inherently produce soluble polyhalides, polysulfides, or organic radical anions. These species migrate across the separator to parasitically react with the Zn anode (the shuttle effect), leading to rapid self-discharge, active mass loss, and severe anode passivation.

### Irreversible Structural Collapse and Phase Transitions

Multi-electron transfer fundamentally requires the accommodation of a larger number of charge carriers (e.g., sequential insertion of H^+^ and Zn^2+^) and significant variations in the oxidation states of transition metals. This induces immense electrostatic repulsion and mechanical strain within the host lattice. For instance, the deep reduction of manganese to Mn^3+^ triggers severe Jahn–Teller distortion, destabilizing the MnO_6_ octahedra and inducing irreversible phase transformations (e.g., forming electrochemically inactive spinel phases). In vanadium-based systems, the excessive extraction/insertion of Zn^2+^ during deep multi-electron cycling often leads to the pulverization of the electrode and the collapse of the layered vanadate structure, permanently trapping Zn^2+^.

### Aggravated Parasitic Reactions (OER/HER)

To fully unlock multi-electron redox couples, a significantly widened electrochemical voltage window is typically required. However, this extended potential range frequently exceeds the thermodynamic stability window of conventional aqueous electrolytes (1.23 V). Consequently, multi-electron cathodes are highly susceptible to severe parasitic reactions. Charging to high potentials to trigger deep oxidation (e.g., Mn^3+^ to Mn^4+^, or halogen oxidation) severely aggravates the Oxygen Evolution Reaction (OER). Conversely, lowering the discharge cut-off voltage to maximize capacity exacerbates the Hydrogen Evolution Reaction (HER) and localized OH^−^ accumulation. These parasitic reactions not only consume the electrolyte and cause cell swelling but also precipitate insulating byproducts (e.g., basic zinc salts) that block the cathode's active sites, accelerating cell failure.

## Analytical Methods of Multi-Electron Transfer Zn-Based Batteries

**Fig. 9:**
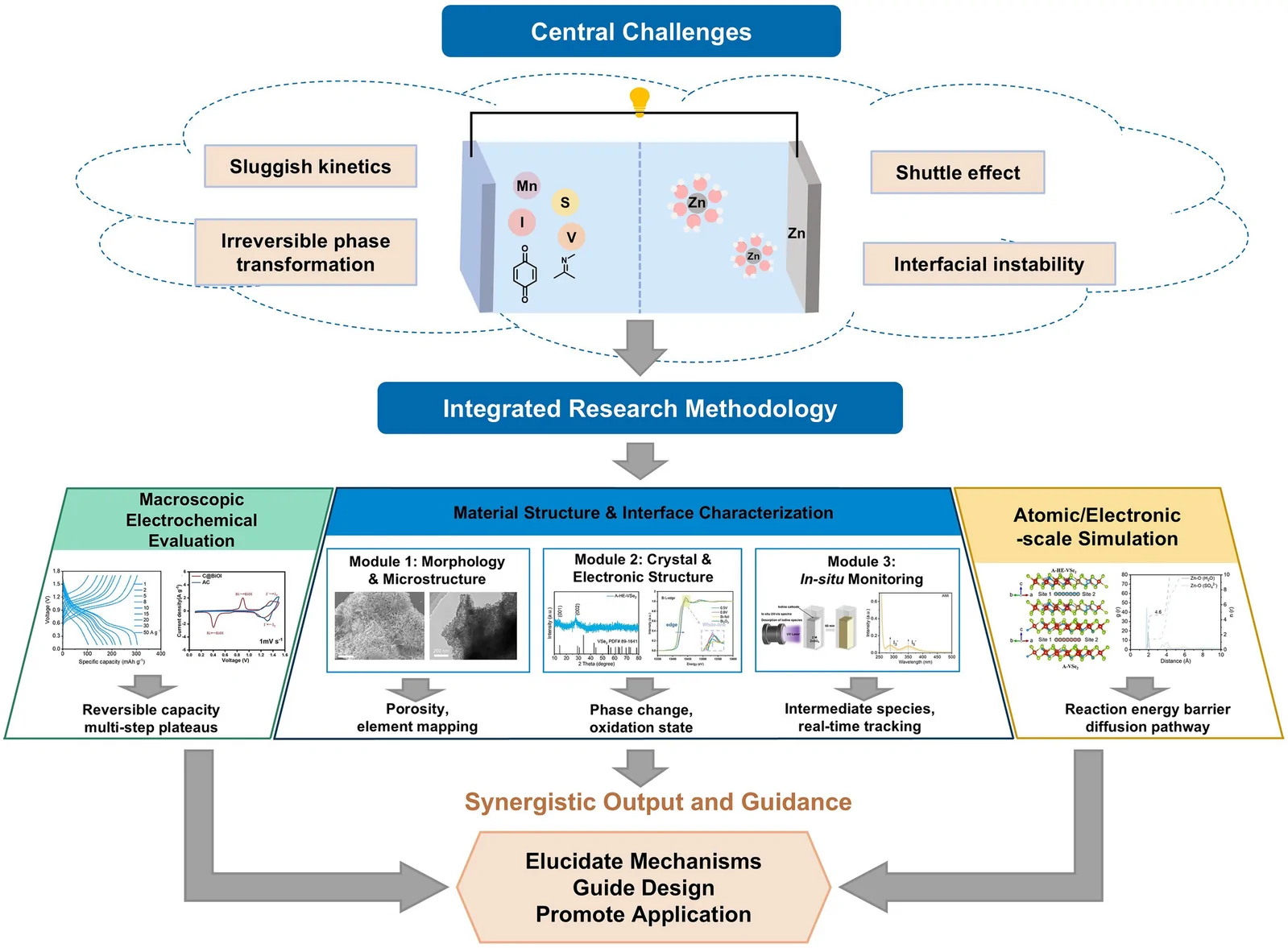
Integrated methodological framework for investigating multi-electron transfer mechanisms in Zn-based batteries [245–248]. Copyright 2025 Wiley–VCH, Copyright 2025 Wiley–VCH, Copyright 2026 Wiley–VCH, Copyright 2025 Wiley–VCH

Elucidating the intrinsic mechanisms of multi‑electron transfer in Zn‑based batteries is fundamental to enhancing their electrochemical performance and paving the way for practical deployment [128]. In practical research, the mechanisms of multi‑electron transfer in Zn‑based batteries can be systematically decoupled through a combination of electrochemical tests, characterization techniques, and theoretical calculations (Fig. 9). This integrated approach provides crucial theoretical insights and experimental evidence, ultimately guiding the rational design of more advanced battery systems toward practical applications [36, 244].

While a diverse array of analytical methods exists, it is scientifically critical to distinguish between multi-electron processes that are directly proved and those that are merely inferred. To rigorously evaluate the literature, we propose a three-tier hierarchy of evidence. Level I (high confidence) consists of *operando* or *in-situ* techniques (e.g., in-situ XANES/EXAFS, in-situ Raman) that capture real-time valence and structural dynamics during cycling, providing direct proof of dynamic electron transfer. Level II (moderate confidence) comprises *ex-situ* spectroscopic and morphological analyses (e.g., XPS, ex-situ XRD, TEM-EDS), which confirm static state changes but are susceptible to relaxation artifacts or sample contamination during preparation. Level III (Supporting/Indirect Evidence) relies on electrochemical signatures (e.g., CV redox peaks, GCD plateaus) and theoretical computations (e.g., DFT). While Level III is indispensable for initial hypotheses and understanding kinetic pathways, it cannot standalone as definitive proof of a multi-electron reaction mechanism. A critical assessment mapping representative cathode system to their respective levels of evidence is summarized in Table 10, highlighting areas where the field currently lacks definitive *operando* validation.

**Table 10:** Critical assessment of the "Level of Evidence" supporting multi-electron transfer mechanisms in representative Zn-based battery cathodes

Cathode family and redox target	Level I (high confidence)	Level II (moderate confidence)	Level III (supporting/indirect)	Overall mechanistic confidence
Manganese Oxides (Mn^4+^ ↔ Mn^2+^)	In-situ XRD (phase tracking)	Ex-situ XPS (valence states), TEM (morphology)	CV multiple peaks, high capacity, DFT	High (Well-documented by multiple groups)
Vanadium Oxides (V^5+^ ↔ V^3+^)	In-situ XAFS (rare but definitive)	Ex-situ XPS (V^4+^/V^3+^ ratios), Ex-situ XRD	Stepwise GCD plateaus, DFT (diffusion barriers)	Moderate to High (Often relies heavily on ex-situ XPS)
Organic (Bipolar)(e.g., n/p dual type)	In-situ FTIR (tracking specific bond cleavage/formation)	Ex-situ XPS (N 1s / O 1s shifts)	CV peak separation, DFT HOMO/LUMO mapping	Moderate (Highly dependent on specific molecule; in-situ data is often lacking)
Halogens (Iodine) (I^−^ ↔ I^+^ ↔ IO_3_^−^​)	In-situ Raman, In-situ UV–Vis (tracking I_3_^−^​, I_2_​, I^+^ species)	Ex-situ XPS (challenging due to volatility)	Capacity matching theoretical values, DFT	High for I^−^ ↔ I^+^; Low to Moderate for 12e^−^ IO_3_^−^​ pathway (heavily inferred)
Sulfur (S_8_​ ↔ S_x_^2−^​ ↔ ZnS)	In-situ Raman (tracking polysulfide evolution)	Ex-situ XPS (S 2p shifting), TEM-EDS	Large voltage hysteresis in GCD, GITT kinetics	Moderate (Solid–solid conversion details remain partially inferred)

### Electrochemical Test Methods

To decipher the complex multi-electron transfer mechanism in Zn-based batteries, researchers routinely employ a combination of key electrochemical methods such as cyclic voltammetry (CV), electrochemical impedance spectroscopy (EIS), galvanostatic charge–discharge (GCD) tests, and the galvanostatic intermittent titration technique (GITT) [249].

CV serves as a fundamental tool for investigating the properties, mechanisms, and kinetic parameters of electrode reactions. This technique applies a linearly varying potential to the electrode, inducing alternating oxidation and reduction reactions within the designated scanning window. The kinetic process can be assessed by analyzing CV curves collected at different scan rates. Specifically, the b-value is derived from the relationship log(*i*) = *b* log(*v*) + log(*a*) [51, 250]. A b value close to 0.5 indicates a diffusion-controlled electrochemical process, while a value of 1.0 suggests a capacitance-like redox process [251, 252]. Furthermore, the quantitative contributions of capacitive (*k*_1_*v*) and diffusion-limited (*k*_2_*v*^1/2^) processes can be distinguished. The observation of multiple, well-separated redox peaks, coupled with a clear correlation between peak current and scan rate, often suggests a multi-step electron transfer process. Such behavior is characteristic of metal ions transitioning between different oxidation states (e.g., V^4+^/V^5+^, Mn^2+^/Mn^3+^/Mn^4+^), where peak positions and shapes can help deduce the underlying multi-electron transfer pathway [253]. In Zn||Organic batteries, redox-active functional groups such as C=O, C=N, N=N, and –NO_2_ undergo redox reactions at different potentials, leading to multiple redox peaks corresponding to each functional group in the CV curves [254]. The potential and current responses of these peaks align with characteristics of multi-electron transfer reactions. Similarly, in Zn||I_2_ or Zn||S batteries, the distinct oxidation states of iodine or sulfur produce separate redox features. Analyzing these CV signatures provides direct electrochemical evidence for multi-electron transfer at the microscopic level, thereby offering a clear and efficient approach to understand the charge storage mechanisms and multi-electron pathways in Zn-based batteries [243, 255].

GCD testing serves as a foundational method for evaluating Zn‑based batteries [256]. Key parameters such as plateau characteristics, voltage hysteresis, and Coulombic efficiency derived from the GCD profiles allow for direct quantification of specific capacity, cycling stability, and energy efficiency. Well-defined voltage plateaus often correspond to stepwise redox reactions or phase transitions, providing preliminary evidence of possible multi-electron transfer.

EIS is primarily employed to probe the kinetics of ion (de)insertion. This technique can resolve charge-transfer resistance at the electrode/electrolyte interface, ion diffusion within the host lattice, and surface adsorption/desorption phenomena. Low charge-transfer resistance and favorable ionic diffusion behavior generally indicate rapid reaction kinetics, which is an essential prerequisite for efficient multi-electron transfer processes [257].

The GITT operates by applying short current pulses and monitoring the voltage relaxation during open-circuit intervals, enabling the calculation of the chemical diffusion coefficient of ions in the electrodes [33]. For organic materials, GITT helps understand the diffusion behavior of ions in the organic electrode and the influence of functional group interactions on electron transfer. If the specific potential changes of functional groups and ion diffusion behavior align with theoretical models of multi-electron reactions, strong support is provided for the occurrence of multi-electron transfer, thereby contributing to the understanding of ion transport and electron transfer mechanisms in organic molecules during the redox reaction [258].

### Characterization Techniques

Characterizing the cathode materials of Zn-based batteries is essential for deepening the understanding of the structure-performance correlation, as well as for revealing the underlying electron transfer mechanisms [259]. This approach provides a critical foundation for optimizing cathode design and synthesis, thereby enhancing overall electrochemical performance.

Commonly employed techniques primarily include morphological and structural analyses [260]. Morphological characterization methods frequently utilize scanning electron microscopy (SEM) and transmission electron microscopy (TEM) technologies. SEM is mainly applied to observe the surface morphology, roughness, porosity, and inter-particle connectivity of electrode materials, which significantly influence ion diffusion. Observed changes in surface structure, such as pore blockage or particle agglomeration, can lead to increased resistance to electron transfer, offering insights into the microscopic origins of performance degradation [241]. In contrast, TEM can be used to investigate particle size, shape, agglomeration, and internal lattice features of electrode materials. It provides direct visualizations of particle distribution and homogeneity, and it enables monitoring of structural evolution after cycling. Furthermore, the elemental analysis capability of TEM can determine the distribution of ions within the cathode material. The migration pathways and distributional variations in elements during electron transfer can be directly observed via TEM-EDS, providing crucial spatial information for studying the charge storage mechanism [261].

A comprehensive, multiscale analysis of material structure is equally critical for elucidating the charge storage mechanisms. Characterizing the structural evolution of cathode materials in multi‑electron transfer reactions requires a combination of techniques that can reveal structural changes, material stability, phase transformations, and their correlation with electrochemical performance during charge‑discharge cycles [262]. Key techniques include X‑ray absorption fine structure (XAFS) spectroscopy, which comprises X‑ray absorption near‑edge structure (XANES) and extended X‑ray absorption fine structure (EXAFS) [263]. These techniques provide detailed electronic and local structural information on electrode materials. XANES accurately determines the oxidation states of metal elements, which is crucial for understanding chemical composition and valence changes. By comparing spectra obtained at different charge–discharge states, the redox processes occurring at the cathode can be identified. EXAFS focuses on analyzing the local atomic environment, including the type, number, and distance of coordination atoms. This information is important for understanding material stability and ionic transport, and it allows monitoring of local structural changes during material cycling [264]. In electron transfer studies, XAFS plays a pivotal role. Electron transfer during charge–discharge cycles accompanies changes in the oxidation states of metal elements, and XANES can directly capture these changes, thereby clarifying the direction and extent of electron transfer [49]. For instance, when Zn^2+^ undergoes a valence change, its absorption edge shift provides direct evidence of this transformation.

X-ray diffraction (XRD) is a powerful tool for studying crystal structures. It can precisely determine the crystalline structure of electrode materials, including unit cell parameters and lattice types. During cycling, XRD can monitor changes in the crystal structure of cathode materials, which helps understand how ion insertion affects the material structures. Structural changes in the electrode crystal lattice are closely linked to electron transfer processes. The embedding of various ions into the cathode lattice can cause lattice expansion, resulting in peak shifts in XRD patterns. Using Bragg’s Law, these shifts can be quantified to infer the ion insertion degree and the associated electron transfer [188].

X-ray photoelectron spectroscopy (XPS) precisely measures the chemical states of elements on the cathode surface. Analysis of the characteristic chemical shifts in the spectra enables accurate determination of the local chemical environments and oxidation states of constituent elements [265].

For organic electrode materials, Fourier transform infrared (FT-IR) spectroscopy is commonly used to analyze molecular structures based on the vibrational modes of functional groups. Tracking spectral changes allows for monitoring the chemical structure evolution induced by electron transfer [81].

It is noteworthy that for Zn||I_2_ batteries, in situ Ultraviolet–Visible (UV–Vis) absorption spectroscopy can be used to investigate the structural changes and electron transfer processes [266]. In Zn||I_2_ batteries, iodine exists in multiple chemical states, such as I_2_, I^−^, and I_3_^−^, each possessing characteristic UV–Vis absorption spectra. According to Lambert Beer’s Law [267], the intensity of an absorption peak is proportional to concentration under specific conditions, allowing concentration changes to be tracked. Therefore, monitoring variations in the characteristic absorption peaks of iodine species in the UV–Vis spectrum provides direct information on their concentration changes. Furthermore, changes in the intensity of these peaks reveal the direction and extent of electron transfer.

In situ Raman spectroscopy can identify different iodine species and directly link spectral changes to electron transfer processes [138]. During charging, the oxidation of I_2_ to I_3_^−^ manifests as a decrease in the characteristic Raman peak intensity of I_2_ alongside the potential emergence of new peaks corresponding to I_3_^−^. This real-time shift in Raman peaks offers direct evidence of electron transfer mechanisms, providing valuable insights into the transfer of electrons between different iodine chemical states.

### Theoretical Calculations

Theoretical calculations used to investigate multi-electron transfer mechanisms include density functional theory (DFT), molecular dynamics (MD) simulations, and first-principles calculations [268]. DFT enables the calculation of electronic structures by solving the Kohn‑Sham equations, which yield the electronic wavefunctions and electron density distribution of the system. This approach facilitates analysis of bonding characteristics, orbital hybridization, and the density of states in cathode materials, thereby revealing electron distribution and transfer pathways. MD simulations are employed to investigate the solvation structure of Zn^2+^ ions and their diffusion behavior within the electrolyte or cathode materials. By calculating mean-square displacement and deriving diffusion coefficients, MD provides critical insights into ion migration rates and pathways. First-principles calculations, based on the fundamental principles of quantum mechanics, precisely solve the electronic wavefunctions and energy eigenvalues of the cathode material system. This provides accurate information about the electronic states, such as electron cloud distribution and charge density, offering a deeper understanding of the microscopic details of electron transfer.

While theoretical calculations provide indispensable microscopic insights, their true predictive power in multi-electron transfer mechanisms relies on rigorous validation against macroscopic experimental data. Specifically, thermodynamic accuracy should be verified by cross-referencing DFT calculated redox potentials and Gibbs free energies with the corresponding multi-step voltage plateaus observed in GCD profiles. From a kinetic perspective, the theoretical ion migration energy barriers derived from MD or NEB simulations must tightly correlate with the empirical diffusion coefficients (*D*_Zn_) quantitatively extracted from GITT measurements. Furthermore, the calculated Density of States (DOS) and bandgap evolution provide the fundamental basis for the material's intrinsic electronic conductivity, which should be directly corroborated by the charge-transfer resistance variations mapped via EIS. Finally, the structural fidelity of the simulated models, including lattice distortions, phase transitions, and local defect engineering, requires rigorous verification using advanced crystallographic techniques such as in situ XRD and HRTEM mapping. This closed-loop correlation between atomic scale theoretical predictions and multi-dimensional experimental observations is paramount for elucidating the authentic multi-electron transfer pathways in high-performance zinc-based batteries.

## Photo-Rechargeable Zn-Based Batteries: A Path to Sustainable Development

**Fig. 10:**
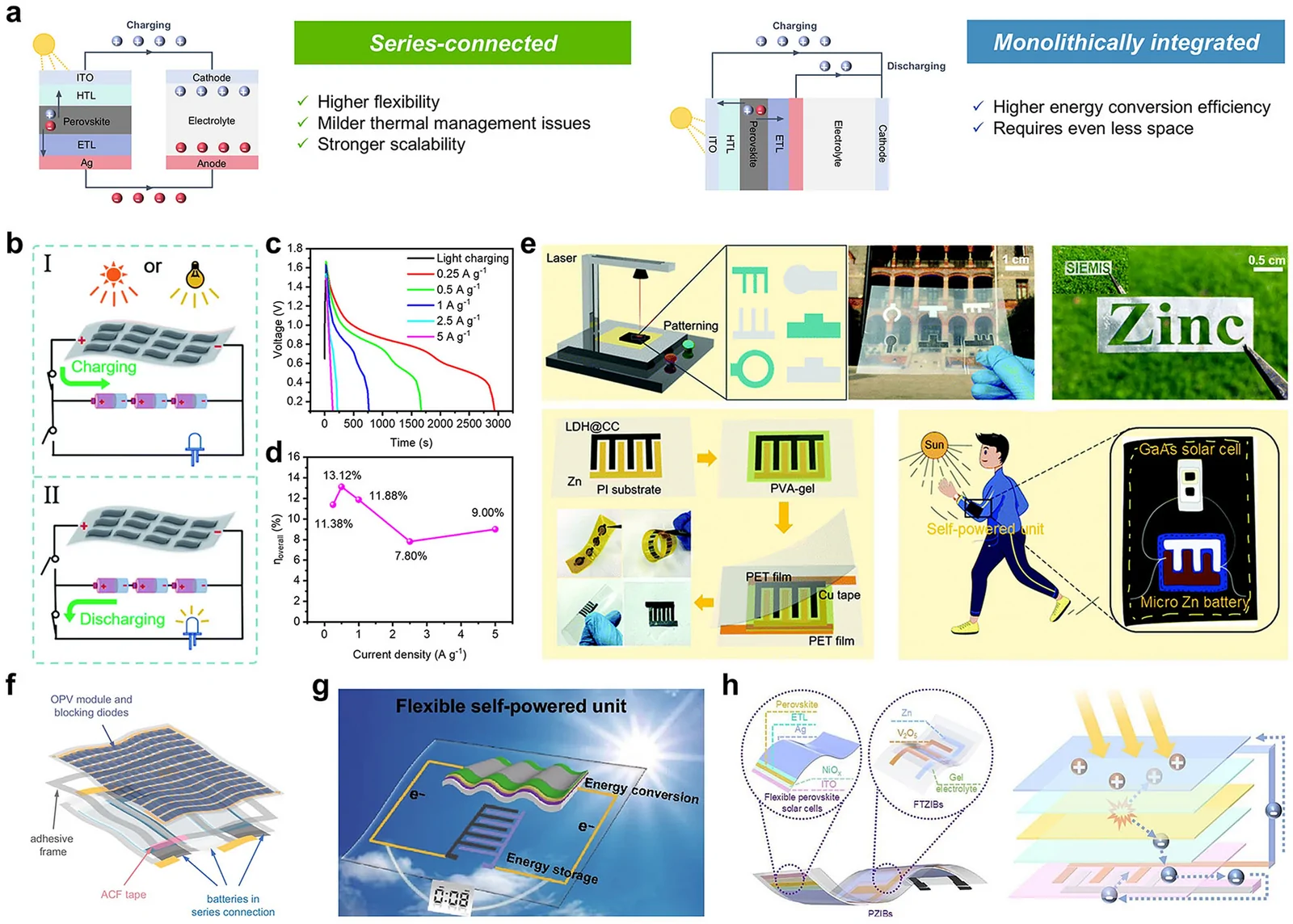
**a** Schematic diagram of the structure of PRZBBs. **b** Schematic diagram of the charging and discharging processes of the PRZBBs. Reproduced with permission [269]. Copyright 2020, American Chemical Society. **c** GCD curves of the PRZBBs and **d** overall conversion efficiency at different current densities. Reproduced with permission [27]. Copyright 2024, Wiley–VCH. **e** Schematic diagram of solar-charging integrated unit. Reproduced with permission [270]. Copyright 2021, Royal Society of Chemistry. **f** Structure schematic of energy harvesting and storage system. Reproduced with permission [271]. Copyright 2024, Nature Publishing. **g** Schematic diagram of the solar-charged self-powered device. Reproduced with permission [272]. Copyright 2020, Nature Publishing. **h** Schematic diagram of the structure and charge transport mechanism of thin-film Zn-based batteries. Reproduced with permission [273]. Copyright 2025, Wiley–VCH

Solar energy offers a highly competitive alternative to traditional fossil fuels due to its abundant reserves, environmental compatibility, and ease of harvesting. Integrating solar cells with Zn-based batteries to create PRZBBs can significantly promote the widespread adoption of renewable energy, accelerate the energy transition, and mitigate climate change, contributing positively to sustainable development. As indicated by Eq. 1, fabricated PRZBBs can achieve a high overall conversion efficiency (*η*_overall_) if high-capacity cathode materials are utilized. Structurally, these integrated devices can be configured in two principal designs: series‑connected or monolithically integrated (Fig. 10a), each presenting distinct advantages and disadvantages in terms of application scenarios and electrochemical performance (Table 11).

**Table 11:** Performance comparison of series-connected structure and monolithically integrated structure PRZBBs

Feature	Series-connected structure	Monolithically integrated structure
Energy conversion efficiency (*η*_overall_)	Low Due to energy transmission loss in external circuits	High More direct energy conversion process
Flexibility	High Photovoltaic cells and batteries can be optimized separately)	Low Tight integration limits optimization space
Space requirements	Large Open design requires more space for components	Compact Suitable for space-limited applications, e.g., portable devices
Thermal management issues	Relatively High Photovoltaic cells and batteries operate separately	Lower Photovoltaic cells and batteries operate together, requiring optimization
Scalability	Strong Can expand by adding photovoltaic or battery units in series	Limited Expansion is constrained post-integration

**Table 12:** Summary of the performance of PRZBBs

Solar cell	Zn-based batteries	Integration	Illumination Conditions (mW cm^−2^)	Specific capacity	*η*_overall_ (%)	Ref
Flexible perovskite solar cells	Zn||MnO_2_	Series-Connected	100	212.4 mAh cm^−3^	5.28	[274]
Perovskite solar cells	Zn-PDPTT||NVO	Series-connected	100	–	13.12	[27]
GaAs Perovskite solar cells	Zn||LDH@CC	Series-connected	951 W m^−2^	20 μAh cm^−2^	23.11	[270]
Organic photovoltaics	Zn||MnO_2_	Series-connected	10	–	6.91	[271]
Organic solar cells	Zn||Kelp-Carbon	Series-connected	0.135	–	17.8	[272]
Carbon-based perovskite solar cells	Zn||Co_2_P-CoP-NiCoO_2_	Monolithically integrated	100	170 mAh g^−1^	6.4	[275]
Janus-jointed photocathode	Zn||Te	Monolithically integrated	7	362 mAh g^−1^	12	[276]
Flexible perovskite solar cell	Zn||V_2_O_5_	Monolithically integrated	100	250 mAh cm^−3^	18.44	[273]

The series‑connected design, which physically links a discrete solar cell and a Zn-based battery, typically offers greater flexibility in component selection and independent optimization. In contrast, the monolithically integrated design combines the light‑harvesting and energy‑storage functions into a single device, often leading to a more compact format and reduced interfacial energy loss. The choice between these architectures depends on the specific requirements of the target application, such as energy density, power demand, stability under intermittent illumination, and scalability. Continued research into interfacial engineering, dynamic stability under practical operating conditions, and scalable fabrication methods will be crucial for translating the high theoretical promise of PRZBBs into practical, high‑performance devices for future self‑powered systems.

To transition PRZBBs from laboratory curiosities to commercially viable autonomous power sources, a rigorous standardization of device-level evaluation is paramount. In addition to reporting the specific capacity and overall energy conversion efficiency, the community must systematically document a matrix of interconnected operational parameters. Specifically, the input solar power is strictly dictated by the light intensity (typically standardized under AM 1.5G, 100 mW cm^−2^) and the effective irradiation area of the integrated photovoltaic component. Furthermore, the charging cutoff conditions, whether governed by a strict voltage upper limit to prevent electrolyte decomposition or a time-controlled pulse, fundamentally determine the state of charge and the boundary safety of the multi-electron cathode. Finally, the stable operating time under continuous or intermittent illumination reveals the true photo-electrochemical fatigue and chemical compatibility of the solar battery interfaces. In the following sections, we review recent progress through the lens of these critical benchmarking metrics (Table 12).

### Series-Connected Structure

In a series-connected configuration, solar cells and Zn-based batteries are typically designed as separate units and interconnected through an external circuit. While operating independently, they collaborate during the charging/discharging processes through the series connection. This architecture offers several advantages. Firstly, it provides high flexibility, allowing for the independent optimization of both components. This enables the selection of the most suitable solar cell technology. For instance, organic, perovskite, and silicon type solar cells can be chosen for the photovoltaic component, while the Zn-based batteries could include Zn||inorganic batteries, Zn||organic batteries, Zn||I_2_ batteries, or Zn||S batteries, among others. Secondly, the physical separation simplifies individual management and protection of each unit, helping to prevent performance mismatches. Finally, this structure is highly scalable. Additional photovoltaic modules or energy storage units can be incorporated as needed, and by connecting them in series, the overall capacity or output voltage of the system can be increased, making it suitable for large-scale energy storage.

To improve the energy density of PRZBBs, MnO_2_ was treated with simple lithium to prepare defect-rich MnO_2-x_ nanosheets. When used as the cathode in Zn-based batteries, this material exhibited a specific energy of 3.63 mWh cm^−2^ with no significant degradation after 5000 cycles. By integrating six series-connected perovskite solar cells with three series-connected Zn-based batteries, stable power supply was achieved for a smart wristband. The use of carbon-based supports with a honeycomb structure increased the MnO_2_ loading to 51.0 mg cm^−2^, and coupling the Zn-based batteries with flexible perovskite solar cells resulted in a PRZBBs with a capacity of 306.7 mAh g^−1^, successfully powering wearable electronics (Fig. 10b) [269]. Our team improved charge storage rates via interfacial modification, and the fabricated Zn||NaV_3_O_8_·1.5H_2_O battery exhibited a capacity of 315.8 mAh g^−1^ and an energy density of 238.0 Wh kg^−1^ at a power density of 3966.7 W kg^−1^ [27]. When coupled with the trans-flexible perovskite solar cells developed by our team, the overall efficiency of the PRZBBs reached 13.1% (Fig. 10c–d). For highly integrated Zn-based batteries and perovskite solar cells, Zn-MnO_2_ micro-batteries and flexible perovskite solar cells were fabricated on PET flexible substrate, achieving a total conversion efficiency of 5.28% for the PRZBBs. Zn-based batteries on PET were fabricated by employed a combination of inkjet printing and electrodeposition, using Ni electroplated between conductive Ag and Zn current collectors to prevent galvanic corrosion between Zn and Ag [274]. A quasi-solid-state electrolyte was used to enhance the safety and mechanical stability of the battery. By connecting two flexible perovskite solar cells in series, the charging voltage required for the Zn||MnO_2_ micro-battery was met. With an open-circuit voltage of 1.94 V, the PRZBBs enabled ultra-fast light charging within 30 s, providing power to functional electronic devices such as LED bulbs and pressure sensors for several minutes. During the integration of Zn-based batteries with photovoltaic cells, active materials may experience cracking or delamination, leading to a decline in electrochemical performance. To address this, laser engraving technology was used to fabricate an array of Zn-based batteries, allowing for fine patterning and manufacturing on flexible conductive substrates (Fig. 10e) [270]. This technique avoids issues related to electrode material deformation that occur with traditional manufacturing methods. By efficiently integrating the Zn-based batteries with a GaAs solar cell, the PRZBBs demonstrated a sustained power supply for 110 s under a current density of 0.52 mA cm^−2^ after just 5 s of solar charging. It also exhibited excellent long-term cycle durability, maintaining an overall conversion efficiency of 23.11%.

While organic photovoltaics and Zn-based batteries hold significant potential for PRZBBs, they still face challenges, such as stability and power output issues in organic photovoltaics. To enhance the electrochemical properties of organic solar cell, a ternary blend of PM6 as the donor material, Y6 as the acceptor, and O-IDTBR was utilized (Fig. 10f) [271]. By paralleling or serially connecting organic solar cell, modules with different output characteristics can be fabricated to meet varying power demands. When integrated with thin-film Zn-based batteries, the resulting PRZBBs with a thickness of only 90 µm demonstrated outstanding flexibility and mechanical durability, achieving an overall conversion efficiency of 6.91%. On a polyimide (PI) substrate, a planar asymmetric printing technique was used to sequentially print carbon cathodes, zinc powder anodes, and a polyacrylamide hydrogel electrolyte doped with Zn(CF_3_SO_3_)_2_ [272]. The fabricated Zn-based batteries exhibited a specific capacity of 196.7 mAh g^−1^ at 0.1 A g^−1^. When integrated with a four-junction organic solar cell, the PRZBBs achieved an overall conversion efficiency of 17.8% (Fig. 10g), with stable operation within a light intensity range of 0.135–4.14 mW cm^−2^. The system also demonstrated adjustable charging times and maintained 95% capacity retention after 100 cycles.

Although the series-connected structure offers greater flexibility in the integration of solar cell and Zn-based batteries, it typically requires external circuits for energy transfer, which may result in some energy loss. Additionally, the series configuration necessitates extra circuit designs and interconnection components, which increases the size and cost of the PRZBBs, thereby reducing the portability of the device. Therefore, series-connected PRZBBs are better suited for applications that demand high scalability and flexibility, particularly for large-scale energy storage systems or high-power applications. This structure allows for the easy addition of more solar cell or battery units to increase capacity or voltage.

### Monolithically Integrated Structure

In the monolithically integrated structure, the solar cell and Zn-based batteries are integrated onto a common substrate, allowing photogenerated charges to be directly transferred to the Zn-based batteries, thereby reducing charge transfer losses. Additionally, the structure of the PRZBBs is more compact and streamlined, significantly enhancing the device’s portability. A heterostructure-based Co_2_P-CoP-NiCoO_2_ nanorod array has been used as a shared electrode for both Zn-based batteries and perovskite solar cells [275]. The resulting sandwich-structured PRZBBs deliver a specific energy of 366 Wh kg^−1^ at 2 A g^−1^, an overall efficiency of 6%, and a stable cycling performance over 200 charge–discharge cycles within a voltage range of 1.40–1.90 V. The Zn||Te battery exhibits a high specific capacity and stable output potential, and Te shows excellent compatibility with perovskite. As a result, integrating Zn||Te with perovskite solar cells enables the fabrication of a high-energy PRZBBs with a higher overall conversion efficiency [276]. On hydrophobic carbon paper, perovskite and Te electrodes were fabricated on opposite sides to design a Janus structure. The Janus structure prevents the water-based electrolyte from damaging the perovskite, while the interface between CH_3_NH_3_PbI_3_ and ZnTe (400) and ZnTe_2_ (200) promotes efficient photogenerated charge transfer. The resulting PRZBBs demonstrate excellent rate capability and cycling stability, with a charge/discharge capacity of 785/720 mAh g^−1^ under illumination, and 358/358 mAh g^−1^ in the dark, achieving an overall conversion efficiency of 12%. Our team has successfully integrated thin-film Zn-based batteries with flexible perovskite solar cells to fabricate portable Zn-based batteries, and we obtained both high overall conversion efficiency and superior specific capacity (Fig. 10h) [273]. The thin-film Zn-based batteries were fabricated on one side of a flexible polyimide substrate, while the perovskite solar cells were integrated on the opposite side, resulting in an exceptionally compact and portable device architecture. To enhance electrochemical and mechanical stability, we employed Ag–Cu alloy as current collectors for the thin-film electrodes. The PRZBBs achieve an outstanding overall conversion efficiency of 18.44% and maintain stable operation during continuous photo-charging/discharging cycles and repeated bending tests.

The monolithically integrated structure requires a high degree of compatibility between the solar cell and the Zn-based batteries, which may necessitate precise structural design between the battery and solar cell units, making the fabrication process more complex. Additionally, the scalability of the system is limited. In practical applications, if an increase in power generation capacity is required, the entire system may need to be redesigned, unlike the series configuration, which allows for more flexible scalability. Therefore, monolithically integrated PRZBBs are primarily applied in wearable electronic devices, smart homes, drones, and other similar applications.

### Strategies for Solar Battery Coupling and Voltage Matching

A rigorous device-level design for PRZBBs must directly address the thermodynamic and kinetic coupling between the multi-electron cathode and the photovoltaic unit. Unlike single-electron intercalation cathodes that often present a single, sloping voltage profile, multi-electron cathodes (such as vanadium oxides, iodine or organics) frequently exhibit multiple, distinct voltage plateaus corresponding to sequential stepwise redox reactions.

The core challenge in the PRZBB architecture is aligning the operational voltage of the solar cell, specifically its maximum power point (*V*_mpp_), with the multistep charging plateaus of the battery. If the *V*_mpp_ is significantly higher than the battery's charging plateau, the excess potential is dissipated as heat, drastically reducing *η*_overall_. Conversely, if *V*_mpp_ falls below the highest voltage plateau required for the final multi-electron oxidation step, the battery cannot be fully charged, severely capping its practical capacity. Therefore, tunable multi-electron cathodes, such as bipolar organic materials or compositionally engineered PBAs, offer significant architectural advantages, as their redox potentials can be molecularly tailored to exactly match the specific *V*_mpp_ of diverse solar cells (e.g., silicon, perovskite, or organic photovoltaics).

In monolithically integrated structures, direct physical coupling necessitates exquisite intrinsic chemical matching, often leading to compromises. However, in series-connected modular designs, advanced charging strategies can be implemented. The integration of miniaturized Maximum Power Point Tracking (MPPT) circuits acts as a dynamic bridge, adjusting the fluctuating solar voltage to consistently meet the dynamic, multi-plateau voltage requirements of the Zn-based battery. Future PRZBB designs must optimize the synergistic trade-off between the complexity of adding MPPT energy management systems and the foundational approach of intrinsically matching the multi-electron cathode redox potentials to the photovoltaic output.

## Conclusion and Perspective

### Conclusion

In this review, we have discussed the advancements in multi-electron transfer reactions in Zn-based batteries, particularly focusing on their applications in PRZBBs. The unique capability of multi-electron transfer reactions enhances the electrochemical performance of Zn-based batteries, enabling higher specific capacity and energy densities. Through a comprehensive analysis of various materials, characterization techniques, and theoretical insights, we highlighted the potential for further optimization of these systems. However, challenges remain, particularly in the design of advanced electrode materials and electrolytes that can efficiently facilitate multi-electron transfer, while maintaining long-term stability and fast charge–discharge kinetics.

### Perspective

#### Development of High-Capacity Multi-electron Transfer Materials

Future research should focus on the design and synthesis of novel electrode materials capable of achieving efficient multi-electron transfer. The cathode material should first be able to accommodate more Zn^2+^ during the multi-electron transfer process, as the ability to store Zn^2+^ directly impacts the battery specific capacity and energy density. Therefore, the material must possess sufficient sites, porous structures, or channels that facilitate the rapid diffusion of Zn^2+^ within the electrode. Secondly, the material must support multi-electron transfer. The cathode material needs to effectively accommodate and facilitate the transfer of two or more electrons, ensuring that the electrode can stably undergo multiple electron transfers during charging and discharging without severe degradation caused by the accumulation of reaction intermediates. Finally, the interface compatibility between the electrode material and the electrolyte is crucial for the performance of Zn-based batteries. Especially during multi-electron transfer, the interfacial reactions between the electrode and the electrolyte will impact the migration rate and reaction efficiency of Zn^2+^.

#### Theoretical Modeling and In-Situ Characterization

To further optimize multi-electron transfer processes, future research should integrate advanced theoretical modeling with in-situ characterization techniques. While density functional theory (DFT), molecular dynamics (MD) simulations, and first-principles calculations have provided crucial theoretical support for studying multi-electron transfer reactions in Zn-based batteries, discrepancies between experimental conditions and computational models can lead to deviations between theoretical predictions and actual experimental results. For instance, theoretical calculations often assume that materials operate under ideal conditions, whereas real-world experiments are influenced by complex factors such as temperature, pressure, and interfacial effects. Therefore, it is essential to establish a more accurate research framework that combines both experimental and computational approaches, using experimental data to validate and refine theoretical models, thereby enhancing the precision and reliability of simulations.

Electrochemical testing methods and characterization techniques provide valuable information on the kinetics, capacity, and stability of Zn-based batteries. However, due to the complexity of the charging and discharging processes, these methods often struggle to fully reveal the details of multi-electron transfer reactions. For example, microstructural changes in electrode materials during charge/discharge cycles may not be captured by traditional electrochemical characterization techniques, which often lack the spatial resolution needed to examine these changes in detail. A key challenge moving forward will be the development of characterization techniques that can provide both temporal and spatial information, such as in-situ/dynamic characterization techniques (e.g., in-situ X-ray diffraction (XRD), in-situ transmission electron microscopy (TEM)). These techniques will allow real-time monitoring of structural changes in electrode materials during battery cycling. Future research should emphasize a multi-scale, multidisciplinary collaborative approach that combines characterization, theoretical simulations, and electrochemical experiments to analyze the multi-electron transfer mechanisms in Zn-based batteries across micro, nano, and macro-scales.

#### Photo-Rechargeable Energy Storage Systems

The integration of multi-electron-transfer Zn-based batteries with solar cells offers a promising strategy for developing high-performance PRZBBs. However, this integration demands excellent interfacial compatibility between the two components, as conventional series connections through external circuits would compromise the volumetric energy density and portability of the resulting PRZBBs. Additionally, energy losses from external resistance would lower the overall conversion efficiency. A further intrinsic challenge arises from the multi‑electron redox mechanisms of the battery itself. Such mechanisms typically involve sequential reaction steps with distinct thermodynamic potentials, leading to multiple voltage plateaus during charging. This dynamic voltage profile inevitably deviates from the relatively fixed output voltage (especially the maximum power point voltage, *V*_mpp_) of the solar cell, creating a fundamental mismatch that complicates direct, efficient coupling and can significantly reduce the energy transfer efficiency if not properly managed.

Integrating Zn‑based thin‑film batteries with flexible solar cells can address part of the structural and interfacial compatibility challenges. To address this voltage mismatch, two complementary strategies can be adopted. From the circuit side, an adaptive maximum power point tracking (MPPT) controller can dynamically adjust the charging voltage to follow the shifting plateaus of the battery. When the required charging voltage for a high-voltage plateau exceeds the open-circuit voltage of the solar cell, a DC-DC boost converter becomes necessary, although it introduces additional conversion losses. The most energy-efficient configuration is one in which the primary high-capacity plateau of the battery lies just below the *V*_mpp_ of the solar cell, allowing direct charging for the majority of the stored. From the materials side, the redox potential of the cathode can be tuned through compositional engineering. For instance, pre-intercalation of metal ions or structural water in vanadium oxides can shift the redox potentials of the V^5+^/V^4+^ and V^4+^/V^3+^ couples, while electron-withdrawing or electron-donating ligands in organic molecules can modulate the redox potentials of C=O or C=N groups. The objective is to design a cathode in which the dominant charging plateau aligns closely with the *V*_mpp_ of the coupled solar cell. This represents a new materials design paradigm for PRZBBs, where voltage tuning is as critical as capacity optimization. Therefore, achieving higher solar energy conversion and storage efficiency necessitates not only a supply‑demand balance model for PRZBBs but also the development of advanced solar charging control systems. These systems should incorporate functions such as maximum power point tracking (MPPT), adaptive multi‑stage constant‑voltage charging, and float charging to dynamically accommodate the battery's multi‑plateau charging characteristics. This approach will help optimize energy transfer and elucidate the storage mechanism of photogenerated charges in Zn‑based batteries under realistic operating conditions. Beyond voltage matching, the practical energy density of PRZBBs is also severely constrained by the mass imbalance between the multi-electron cathode and the Zn anode. A critical but often overlooked consequence of high-capacity multi-electron cathodes is that they require a correspondingly high areal capacity from the Zn anode. Thick Zn foils, while ensuring cycling stability, drastically dilute the practical cell-level energy density, with higher N/P ratios. An anode-free design, in which Zn is plated from the electrolyte onto a lightweight current collector, offers a compelling solution by eliminating excess Zn. However, these designs demand exceptionally high Zn plating/stripping Coulombic efficiency (> 99.9%) and dendrite-free morphology, both of which remain significant research targets.

**Fig. 11:**
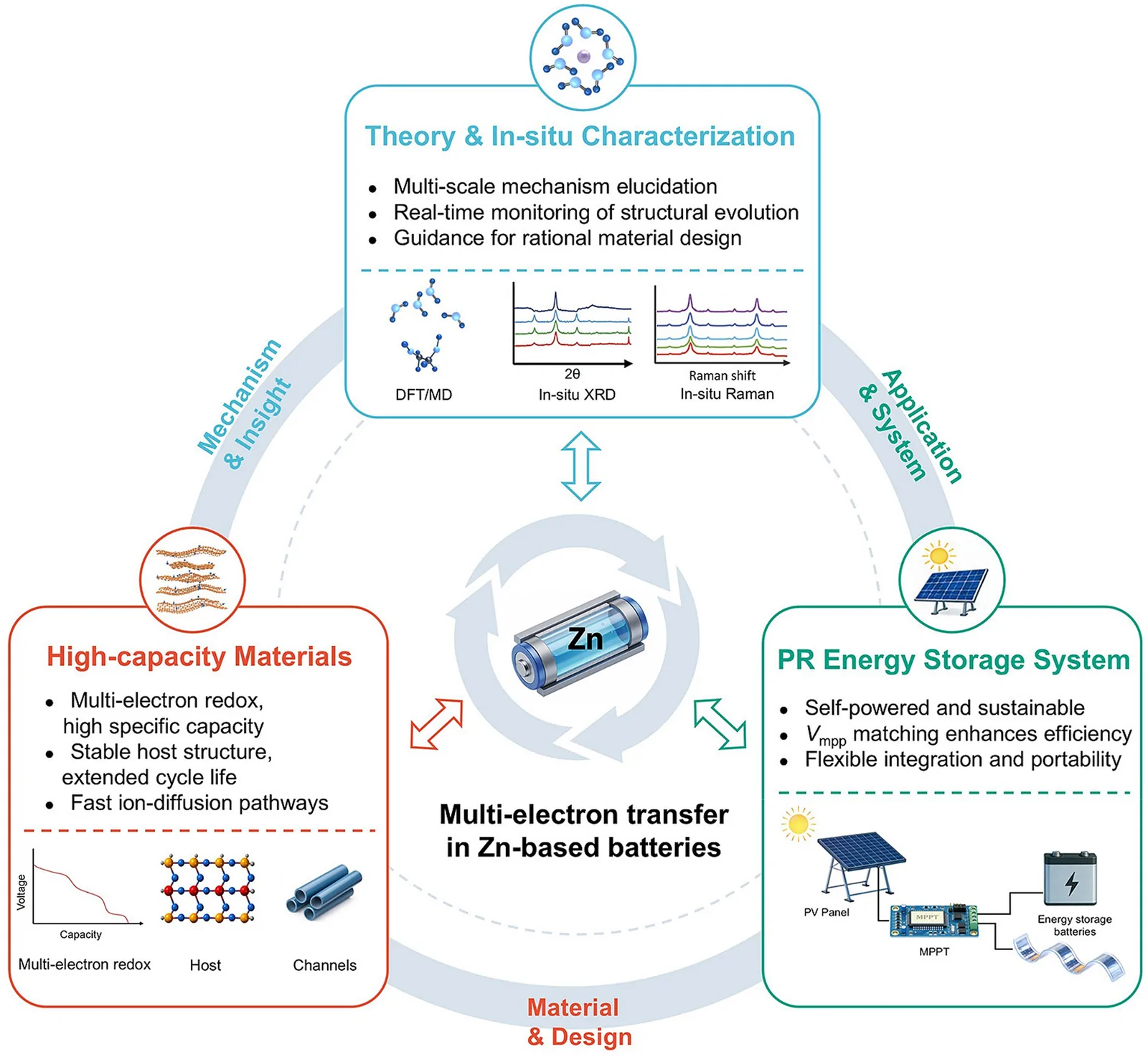
Potential future research avenues for PRZBBs

While the exploration of multi-electron transfer mechanisms has unlocked unprecedented theoretical capacities for zinc-based batteries, bridging the gap between academic research and practical commercialization requires a rigorous assessment against five key industrial metrics: (*i*) Cost: The inherent affordability of metallic zinc and aqueous solvents is a primary driver for this technology. However, a significant gap remains in translating this to a low system level cost (kWh). Current high-performance prototypes heavily rely on expensive highly concentrated electrolytes, customized functional additives, and complex cathode synthesis, which must be optimized for scalable manufacturing. (*ii*) Safety: Aqueous zinc batteries are intrinsically non-flammable, offering a massive safety advantage over lithium-ion systems. Nevertheless, under practical deployment, continuous parasitic gas evolution (such as H_2_ and O_2_) creates severe internal pressure, posing critical risks of swelling, leakage or rupture in sealed, large-format commercial pouch cells. (*iii*) Rate Capability: Multi-electron reactions often involve complex phase transitions and sluggish solid–solid conversions, leading to inferior power densities. To meet commercial fast charging and high power demands, future research must focus on integrating advanced catalytic materials and rationally designed dual phase composites to effectively lower the activation energy barriers and accelerate these sluggish conversion kinetics. (*iv*) Low-Temperature Performance: The freezing of aqueous electrolytes and the exponential increase in charge transfer resistance at sub-zero temperatures severely restrict geographic deployability. Developing cost effective anti-freezing strategies, such as localized high concentration electrolytes or eutectic solvents, without compromising the overall system cost is an ongoing challenge. (*v*) Cycle Life: Perhaps the most formidable commercial gap lies in long-term cyclability. While thousands of cycles are routinely reported in coin cells utilizing massive zinc excess, these results rarely translate to practical applications. Under stringent commercial parameters, including lean electrolytes, high areal mass loadings and low negative to positive capacity ratios, the cycle life declines sharply because of accelerated water consumption, irreversible active material loss and severe dendrite penetration. Ultimately, the successful commercialization of multi-electron zinc batteries will depend on a paradigm shift from solely maximizing theoretical capacity to systematically balancing these five interdependent pragmatic metrics. Future research efforts should focus on the development of innovative electrode architectures, coupled with improved electrolyte formulations and advanced modeling techniques to fully harness the capabilities of multi-electron transfer processes (Fig. 11). This will pave the way for the next generation of high-performance, photo-rechargeable energy storage devices that can contribute to the transition towards sustainable energy solutions.

### Perspective: A Roadmap Toward Practical PRZBBs

**Fig. 12:**
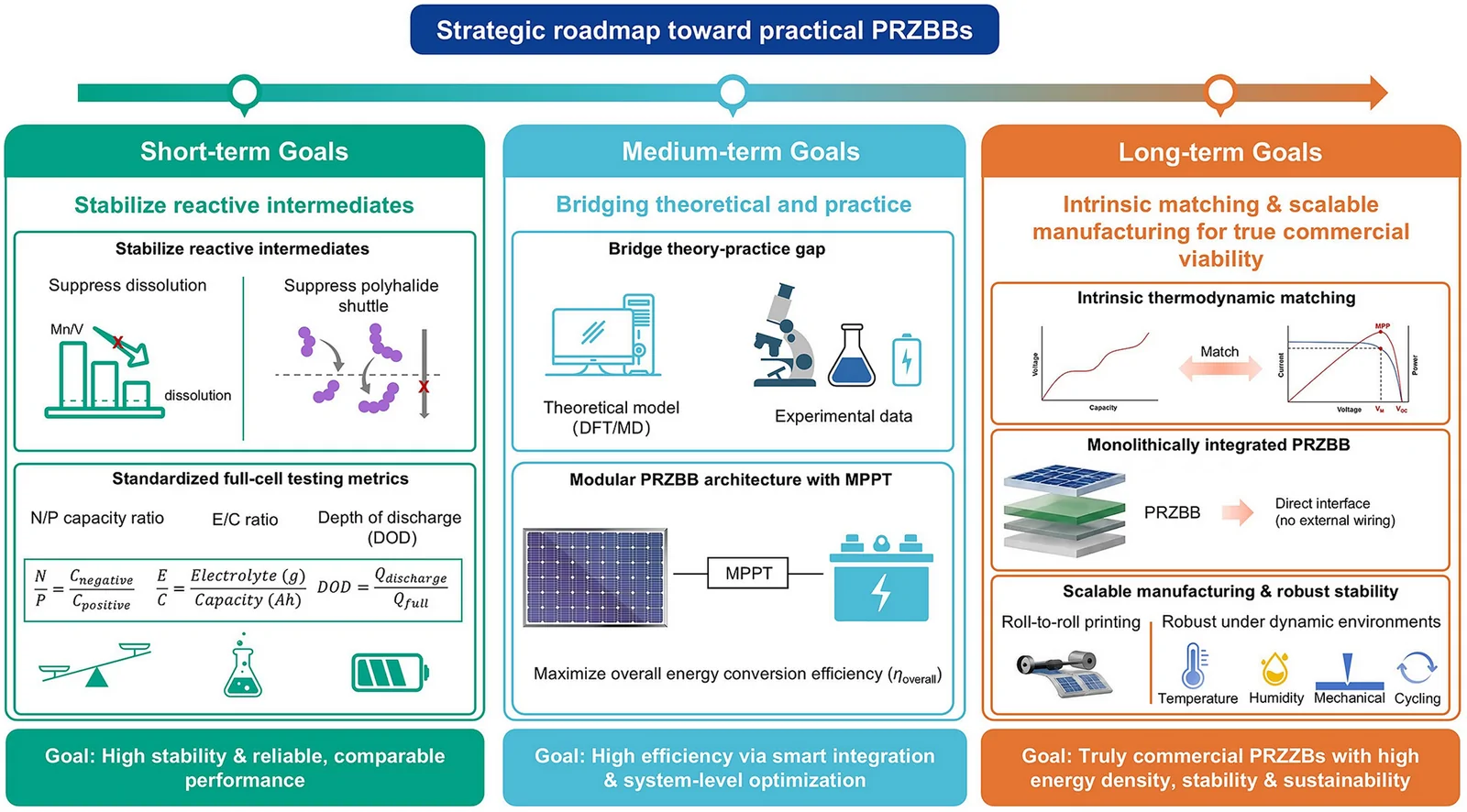
A roadmap toward practical PRZBBs

Realizing practical PRZBBs requires a strategic roadmap spanning short-, medium- and long-term objectives (Fig. 12). In the short term, research must prioritize stabilizing highly reactive intermediates to prevent active material loss, for example by mitigating Mn^2+^ or vanadium dissolution and completely suppressing polyhalide shuttle effects, while simultaneously adopting rigorous and standardized full-cell testing metrics such as the Negative to Positive (N/P) capacity ratio, electrolyte to capacity (E/C) ratio and realistic depths of discharge (DOD). Medium-term goals shift toward bridging the gap between theoretical models and practical environments using advanced *operando* characterization, alongside developing modular, series-connected PRZBB architectures equipped with Maximum Power Point Tracking (MPPT) circuits to overcome voltage mismatches and maximize overall energy conversion efficiency (*η*_overall_). Ultimately, the long-term vision demands intrinsic thermodynamic matching of cathode redox potentials to the photovoltaic *V*_mpp_ for true monolithically integrated devices, coupled with scalable manufacturing (e.g., roll-to-roll printing) and robust stability under dynamic environmental conditions to achieve true commercial viability.

## References

[1] S. Cho, M.R. Khawar, Y. Ra, S. Jang, D. Lee et al., Advances in 3d-printed triboelectric nanogenerators and supercapacitors for self-sustainable energy systems. Mater. Today **85**, 189–211 (2025). 10.1016/j.mattod.2025.02.010

[2] G. Whang, R. Chen, J.T.T. Yu, C. Choi, P. McNeil et al., Do micropower sources meet the needs of the internet of things? Adv. Energy Mater. **15**(48), e03921 (2025). 10.1002/aenm.202503921

[3] D. Yang, C. Xin, H.-K. Liu, Y. Zhang, T. Xiong, From photo-assisted to photo-rechargeable: advancing zn-ion batteries with light-driven energy storage. Green Energy Environ. **11**(2), 387–400 (2026). 10.1016/j.gee.2025.12.002

[4] L. Tang, H. Peng, J. Kang, H. Chen, M. Zhang et al., Zn-based batteries for sustainable energy storage: strategies and mechanisms. Chem. Soc. Rev. **53**(10), 4877–4925 (2024). 10.1039/d3cs00295k 38595056

[5] K. Jin, Y. Yu, Principles, progress, and prospects of photo-rechargeable zinc-ion batteries. J. Energy Chem. **104**, 382–396 (2025). 10.1016/j.jechem.2024.12.058

[6] T. Yamamoto, M. Hishinuma, A. Yamamoto, Zn∣ZnI_2_∣iodine secondary battery using iodine-nylon-6 adduct as positive electrode, and its charge-discharge performance. Inorg. Chim. Acta **86**(2), L47–L49 (1984). 10.1016/S0020-1693(00)82320-8

[7] M. Minakshi, K. Nallathamby, D.R.G. Mitchell, Electrochemical characterization of an aqueous lithium rechargeable battery: the effect of CeO_2_ additions to the MnO_2_ cathode. J. Alloys Compd. **479**(1–2), 87–90 (2009). 10.1016/j.jallcom.2008.09.082

[8] L. Zhang, L. Chen, X. Zhou, Z. Liu, Towards high-voltage aqueous metal-ion batteries beyond 1.5 V: the zinc/zinc hexacyanoferrate system. Adv. Energy Mater. **5**(2), 1400930 (2015). 10.1002/aenm.201400930

[9] M.H. Alfaruqi, V. Mathew, J. Gim, S. Kim, J. Song et al., Electrochemically induced structural transformation in a γ-MnO_2_ cathode of a high capacity zinc-ion battery system. Chem. Mater. **27**(10), 3609–3620 (2015). 10.1021/cm504717p

[10] L. Zhang, L. Chen, X. Zhou, Z. Liu, Morphology-dependent electrochemical performance of zinc hexacyanoferrate cathode for zinc-ion battery. Sci. Rep. **5**(1), 18263 (2015). 10.1038/srep18263 PMC4680913 26669272

[11] D. Kundu, B.D. Adams, V. Duffort, S.H. Vajargah, L.F. Nazar et al., A high-capacity and long-life aqueous rechargeable zinc battery using a metal oxide intercalation cathode. Nat. Energy **1**(10), 16119 (2016). 10.1038/nenergy.2016.119

[12] H. Pan, B. Li, D. Mei, Z. Nie, Y. Shao et al., Controlling solid–liquid conversion reactions for a highly reversible aqueous zinc–iodine battery. ACS Energy Lett. **2**(12), 2674–2680 (2017). 10.1021/acsenergylett.7b00851

[13] M.M. Gross, A. Manthiram, Rechargeable zinc-aqueous polysulfide battery with a mediator-ion solid electrolyte. ACS Appl. Mater. Interfaces **10**(13), 10612–10617 (2018). 10.1021/acsami.8b00893 29561586

[14] Z. Wang, J. Huang, Z. Guo, X. Dong, Y. Liu et al., A metal-organic framework host for highly reversible dendrite-free zinc metal anodes. Joule **3**(5), 1289–1300 (2019). 10.1016/j.joule.2019.03.012

[15] M. Na, Y. Oh, H.R. Byon, Effects of Zn^2+^ and H^+^ association with naphthalene diimide electrodes for aqueous Zn-ion batteries. Chem. Mater. **32**(16), 6990–6997 (2020). 10.1021/acs.chemmater.0c01790

[16] S. Ding, M. Zhang, R. Qin, J. Fang, H. Ren et al., Oxygen-deficient β-MnO_2_@ graphene oxide cathode for high-rate and long-life aqueous zinc ion batteries. Nano-Micro Lett. **13**(1), 173 (2021). 10.1007/s40843-021-00727-7 PMC8363675 34387758

[17] Y. Zeng, J. Xu, Y. Wang, S. Li, D. Luan et al., Formation of CuMn Prussian blue analog double-shelled nanoboxes toward long-life Zn-ion batteries. Angew. Chem. Int. Ed. **61**(48), e202212031 (2022). 10.1002/anie.202212031 36177990

[18] S. Zhao, S. Wang, J. Guo, L. Li, C. Li et al., Sodium‐ion and polyaniline co‐intercalation into ammonium vanadate nanoarrays induced enlarged interlayer spacing as high‐capacity and stable cathodes for flexible aqueous zinc‐ion batteries. Adv. Funct. Mater. **33**(48), 2305700 (2023). 10.1002/adfm.202305700

[19] Y. Wang, X. Jin, J. Xiong, Q. Zhu, Q. Li et al., Ultrastable electrolytic Zn–I_2_ batteries based on nanocarbon wrapped by highly efficient single-atom Fe-NC iodine catalysts. Adv. Mater. **36**(30), 2404093 (2024). 10.1002/adma.202404093 38717804

[20] J. Lei, J. Lin, Y. Zuo, Y. Yan, Y. Lu et al., Reconstructing interfacial electric double layer for efficient sulfur conversion reaction in aqueous zinc sulfur batteries. Nat. Commun. **16**(1), 10144 (2025). 10.1038/s41467-025-63969-z PMC12630962 41257842

[21] H. Chen, X. Li, K. Fang, H. Wang, J. Ning et al., Aqueous zinc‐iodine batteries: from electrochemistry to energy storage mechanism. Adv. Energy Mater. **13**(41), 2302187 (2023). 10.1002/aenm.202302187

[22] J. Li, Z. Liu, S. Han, P. Zhou, B. Lu et al., Hetero nucleus growth stabilizing zinc anode for high-biosecurity zinc-ion batteries. Nano-Micro Lett. **15**(1), 237 (2023). 10.1007/s40843-023-00602-4 PMC10603014 37882885

[23] J. Cui, Z. Guo, J. Yi, X. Liu, K. Wu et al., Organic cathode materials for rechargeable zinc batteries: mechanisms, challenges, and perspectives. Chemsuschem **13**(9), 2160–2185 (2020). 10.1002/cssc.202000327 32043825

[24] Z. Wang, N. Liu, Q. Li, X. Wang, J. Liu et al., Dual redox reactions of silver hexacyanoferrate prussian blue analogue enable superior electrochemical performance for zinc-ion storage. Angew. Chem. Int. Ed. **64**(4), e202416392 (2025). 10.1002/anie.202416392 39401949

[25] W. Zhang, M. Wang, J. Ma, H. Zhang, L. Fu et al., Bidirectional atomic iron catalysis of sulfur redox conversion in high‐energy flexible Zn–S battery. Adv. Funct. Mater. **33**(11), 2210899 (2023). 10.1002/adfm.202210899

[26] R. Li, R. Zhao, A. Tang, C. Wan, X. Meng et al., Hydration-driven structural heterogeneity for robust Zn^2+^ storage in vanadium oxide cathodes. Adv. Funct. Mater. **36**(19), e202520415 (2026). 10.1002/adfm.202520415

[27] M. Chen, X. Guo, X. Jiang, B. Farhadi, X. Guo et al., Multi-group polymer coating on Zn anode for high overall conversion efficiency photorechargeable zinc-ion batteries. Angew. Chem. Int. Ed. **63**(39), e202410011 (2024). 10.1002/anie.202410011 38937260

[28] Y. Cao, S. Ju, Q. Zhang, K. Gao, A. Marcelli et al., Recent progress in aqueous zinc-ion batteries based on conversion-type cathodes. Adv. Powder Mater. **4**(2), 479 (2025). 10.1016/j.apmate.2025.100278

[29] N. Zhang, J.-C. Wang, Y.-F. Guo, P.-F. Wang, Y.-R. Zhu et al., Insights on rational design and energy storage mechanism of Mn-based cathode materials towards high performance aqueous zinc-ion batteries. Coord. Chem. Rev. **479**(15), 215009 (2023). 10.1016/j.ccr.2022.215009

[30] X. Chen, H. Zhang, J.-H. Liu, Y. Gao, X. Cao et al., Vanadium-based cathodes for aqueous zinc-ion batteries: mechanism, design strategies and challenges. Energy Storage Mater. **50**, 21–46 (2022). 10.1016/j.ensm.2022.04.040

[31] M. Cui, Y. Zhu, H. Lei, A. Liu, F. Mo et al., Anion-cation competition chemistry for comprehensive high-performance prussian blue analogs cathodes. Angew. Chem. Int. Ed. **63**(23), e202405428 (2024). 10.1002/anie.202405428 38563631

[32] Z. Li, J. Tan, Y. Wang, C. Gao, Y. Wang et al., Building better aqueous Zn-organic batteries. Energy Environ. Sci. **16**(6), 2398–2431 (2023). 10.1039/d3ee00211j

[33] H. Chen, X. Li, K. Fang, H. Wang, J. Ning et al., Aqueous zinc-iodine batteries: From electrochemistry to energy storage mechanism. Adv. Energy Mater. **13**(41), 2302187 (2023). 10.1002/aenm.202302187

[34] X. Wang, L. Liu, Z. Hu, C. Peng, C. Han et al., High energy density aqueous zinc–chalcogen (S, Se, Te) batteries: Recent progress, challenges, and perspective. Adv. Energy Mater. **13**(44), 2302927 (2023). 10.1002/aenm.202302927

[35] P. He, J. Liu, X. Zhao, Z. Ding, P. Gao et al., A three-dimensional interconnected V_6_O_13_ nest with a V^5+^-rich state for ultrahigh Zn ion storage. J. Mater. Chem. A **8**(20), 10370–10376 (2020). 10.1039/d0ta03165h

[36] X. Chen, W. Li, D. Reed, X. Li, X. Liu, On energy storage chemistry of aqueous Zn-ion batteries: from cathode to anode. Electrochem. Energy Rev. (2023). 10.1007/s41918-023-00194-6

[37] P. Ruan, S. Liang, B. Lu, H.J. Fan, J. Zhou, Design strategies for high‐energy‐density aqueous zinc batteries. Angew. Chem. Int. Ed. **61**(17), e202200598 (2022). 10.1002/anie.202200598 35104009

[38] T. Liu, C. Lei, H. Wang, J. Li, P. Jiang et al., Aqueous electrolyte with weak hydrogen bonds for four-electron zinc–iodine battery operates in a wide temperature range. Adv. Mater. **36**(32), 2405473 (2024). 10.1002/adma.202405473 38837833

[39] P. Canepa, G. Sai Gautam, D.C. Hannah, R. Malik, M. Liu et al., Odyssey of multivalent cathode materials: open questions and future challenges. Chem. Rev. **117**(5), 4287–4341 (2017). 10.1021/acs.chemrev.6b00614 28269988

[40] G. Li, L. Sun, S. Zhang, C. Zhang, H. Jin et al., Developing cathode materials for aqueous zinc ion batteries: challenges and practical prospects. Adv. Funct. Mater. **34**(5), 2301291 (2023). 10.1002/adfm.202301291

[41] Q. Li, K.K. Abdalla, J. Xiong, Z. Song, Y. Wang et al., High-energy and durable aqueous Zn batteries enabled by multi-electron transfer reactions. Energy Mater. **4**(4), 400040 (2024). 10.20517/energymater.2024.12

[42] J. Luan, H. Yuan, J. Liu, C. Zhong, Recent advances on charge storage mechanisms and optimization strategies of Mn-based cathode in zinc–manganese oxides batteries. Energy Storage Mater. **66**, 103206 (2024). 10.1016/j.ensm.2024.103206

[43] W. Su, Z. Chen, X. Wang, P. Cui, M. Yao et al., Dynamic transport of Zn^2+^ in ionic liquid intercalated V_2_O_5_ cathode for high-performance aqueous Zn-ion batteries. Chem. Eng. J. **504**, 158827 (2025). 10.1016/j.cej.2025.158827

[44] Y. Liu, J. Zhi, M. Sedighi, M. Han, Q. Shi et al., Mn^2+^ ions confined by electrode microskin for aqueous battery beyond intercalation capacity. Adv. Energy Mater. **10**(42), 2002578 (2020). 10.1002/aenm.202002578

[45] J. Wu, J. Huang, X. Chi, J. Yang, Y. Liu, Mn^2+^/I^−^ hybrid cathode with superior conversion efficiency for ultrahigh-areal-capacity aqueous zinc batteries. ACS Appl. Mater. Interfaces **14**(48), 53627–53635 (2022). 10.1021/acsami.2c12997 36417686

[46] J. Huang, J. Zeng, K. Zhu, R. Zhang, J. Liu, High-performance aqueous zinc–manganese battery with reversible Mn^2+^/Mn^4+^ double redox achieved by carbon coated MnOx nanoparticles. Nano-Micro Lett. **12**(1), 110 (2020). 10.1007/s40820-020-00445-x PMC7770770 34138114

[47] X. Ye, D. Han, G. Jiang, C. Cui, Y. Guo et al., Unraveling the deposition/dissolution chemistry of MnO_2_ for high-energy aqueous batteries. Energy Environ. Sci. **16**(3), 1016–1023 (2023). 10.1039/d3ee00018d

[48] A. Zhang, R. Zhao, Y. Wang, J. Yang, C. Wu et al., Regulating the electronic structure of manganese-based materials to optimize the performance of zinc-ion batteries. Energy Environ. Sci. **16**(8), 3240–3301 (2023). 10.1039/d3ee01344h

[49] Y. Wang, H. Chen, S. Zhang, L. Hou, X. Li et al., Recent advances of vanadium-based cathodes toward aqueous Zn-ion batteries. Next Energy **4**, 100119 (2024). 10.1016/j.nxener.2024.100119

[50] N. Zhang, Y. Dong, M. Jia, X. Bian, Y. Wang et al., Rechargeable aqueous Zn–V_2_O_5_ battery with high energy density and long cycle life. ACS Energy Lett. **3**(6), 1366–1372 (2018). 10.1021/acsenergylett.8b00565

[51] Y. Wang, Z. Wang, W.K. Pang, W. Lie, J.A. Yuwono et al., Solvent control of water O–H bonds for highly reversible zinc ion batteries. Nat. Commun. **14**(1), 2720 (2023). 10.1038/s41467-023-38384-x PMC10175258 37169771

[52] J. Guo, L. Li, J. Luo, W. Gong, R. Pan et al., Polypyrrole‐assisted nitrogen doping strategy to boost vanadium dioxide performance for wearable nonpolarity supercapacitor and aqueous zinc-ion battery. Adv. Energy Mater. **12**(41), 2201481 (2022). 10.1002/aenm.202201481

[53] B. Yang, Y. Ma, D. Bin, H. Lu, Y. Xia, Ultralong-life cathode for aqueous zinc-organic batteries via pouring 9,10-phenanthraquinone into active carbon. ACS Appl. Mater. Interfaces **13**(49), 58818–58826 (2021). 10.1021/acsami.1c20087 34846135

[54] L. Lin, Z. Xue, T. Qiu, J. Zhu, G. Zhang et al., Non-conjugated linkage enabling a quinone-based cathode material with long cycle life and high energy density for aqueous zinc batteries. Energy Environ. Sci. **17**(18), 6499–6506 (2024). 10.1039/d4ee02097a

[55] K.W. Nam, H. Kim, Y. Beldjoudi, T.W. Kwon, D.J. Kim et al., Redox-active phenanthrenequinone triangles in aqueous rechargeable zinc batteries. J. Am. Chem. Soc. **142**(5), 2541–2548 (2020). 10.1021/jacs.9b12436 31895548

[56] J. Liu, Y. Zhou, G. Xing, M. Qi, Z. Tang et al., 2d conductive metal–organic framework with anthraquinone built-in active sites as cathode for aqueous zinc ion battery. Adv. Funct. Mater. **34**(21), 2312636 (2024). 10.1002/adfm.202312636

[57] L. Lin, Z. Lin, J. Zhu, K. Wang, W. Wu et al., A semi-conductive organic cathode material enabled by extended conjugation for rechargeable aqueous zinc batteries. Energy Environ. Sci. **16**(1), 89–96 (2022). 10.1039/d2ee02961h

[58] Y. Li, L. Liu, Y. Lu, R. Shi, Y. Ma et al., High-energy-density quinone-based electrodes with [Al(OTf)]^2+^ storage mechanism for rechargeable aqueous aluminum batteries. Adv. Funct. Mater. **31**(26), 2102063 (2021). 10.1002/adfm.202102063

[59] Y. Zhang, Z. Song, L. Miao, Y. Lv, L. Gan et al., Duodecuple H-bonded NH^4+^ storage in multi-redox-site N-heterocyclic cathode for six-electron zinc–organic batteries. Adv. Funct. Mater. **34**(45), 2405710 (2024). 10.1002/adfm.202405710

[60] J. Yang, H. Hua, H. Yang, P. Lai, M. Zhang et al., A high utilization and environmentally sustainable all-organic aqueous zinc-ion battery enabled by a molecular architecture design. Adv. Energy Mater. **13**(25), 2204005 (2023). 10.1002/aenm.202204005

[61] Z. Liu, L. Gong, G. Gao, J. Wang, H. Pan et al., Transformation of vulnerable imine bond into aromatic in 3d cof for ultrastable lithium-ion batteries. Energy Storage Mater. **74**, 103931 (2024). 10.1016/j.ensm.2024.103931

[62] W. Wang, V.S. Kale, Z. Cao, S. Kandambeth, W. Zhang et al., Phenanthroline covalent organic framework electrodes for high-performance zinc-ion supercapattery. ACS Energy Lett. **5**(7), 2256–2264 (2020). 10.1021/acsenergylett.0c00903

[63] Y.J. Gao, G.F. Li, F. Wang, J. Chu, P. Yu et al., A high-performance aqueous rechargeable zinc battery based on organic cathode integrating quinone and pyrazine. Energy Storage Mater. **40**, 31–40 (2021). 10.1016/j.ensm.2021.05.002

[64] Y. Wang, J. Wang, J. Peng, Y. Jiang, Y. Zhu et al., Crafting core–shell heterostructures with enriched active centers for high-energy-density symmetric lithium-ion batteries. ACS Nano **18**(35), 23958–23967 (2024). 10.1021/acsnano.3c11780 39169879

[65] Y. Zhang, M. Li, Z. Li, Y. Lu, H. Li et al., A high capacity p-type organic cathode material for aqueous zinc batteries. Angew. Chem. Int. Ed. **63**(48), e202410342 (2024). 10.1002/anie.202410342 39223696

[66] H. Zhang, L. Zhong, J. Xie, F. Yang, X. Liu et al., A cof-like n-rich conjugated microporous polytriphenylamine cathode with pseudocapacitive anion storage behavior for high-energy aqueous zinc dual-ion batteries. Adv. Mater. **33**(34), e2101857 (2021). 10.1002/adma.202101857 34259360

[67] S. Gong, K. Sun, N. Cao, D. Chao, X. Jia et al., Potassium phthalimide doped with delta-site structures to construct ultra-long cycle life aqueous Zn-polymer batteries. Adv. Funct. Mater. **34**(51), 2409805 (2024). 10.1002/adfm.202409805

[68] X. Qiu, J. Xu, K. Zhou, X. Huang, M. Liao et al., Molecular tailoring of p–type organics for zinc batteries with high energy density. Angew. Chem. Int. Ed. **62**(30), e202304036 (2023). 10.1002/anie.202304036 37231580

[69] W. Wu, H.-Y. Shi, Z. Lin, X. Yang, C. Li et al., The controlled quinone introduction and conformation modification of polyaniline cathode materials for rechargeable aqueous zinc-polymer batteries. Chem. Eng. J. **419**, 129659 (2021). 10.1016/j.cej.2021.129659

[70] U. Mittal, F. Colasuonno, A. Rawal, M. Lessio, D. Kundu, A highly stable 1.3 v organic cathode for aqueous zinc batteries designed in-situ by solid-state electrooxidation. Energy Storage Mater. **46**, 129–137 (2022). 10.1016/j.ensm.2022.01.004

[71] M.H. Lee, G. Kwon, H. Lim, J. Kim, S.J. Kim et al., High-energy and long-lasting organic electrode for a rechargeable aqueous battery. ACS Energy Lett. **7**(10), 3637–3645 (2022). 10.1021/acsenergylett.2c01535

[72] H. Dai, Y. Chen, Y. Cao, M. Fu, L. Guan et al., Structural isomers: small change with big difference in anion storage. Nano-Micro Lett. **16**, 13 (2023). 10.1007/s40820-023-01239-7 PMC10643786 37955747

[73] H.Y. Shi, Y.J. Ye, K. Liu, Y. Song, X. Sun, A long-cycle-life self-doped polyaniline cathode for rechargeable aqueous zinc batteries. Angew. Chem. Int. Ed. **57**(50), 16359–16363 (2018). 10.1002/anie.201808886 30307094

[74] Z. Song, Q. Huang, Y. Lv, L. Gan, M. Liu, Multi-n-heterocycle donor-acceptor conjugated amphoteric organic superstructures for superior zinc batteries. Angew. Chem. Int. Ed. **64**(6), e202418237 (2025). 10.1002/anie.202418237 39496567

[75] N. Wang, Z.W. Guo, Z.G. Ni, J. Xu, X. Qiu et al., Molecular tailoring of an n/p-type phenothiazine organic scaffold for zinc batteries. Angew. Chem. Int. Ed. **60**(38), 20826–20832 (2021). 10.1002/anie.202106238 34278677

[76] L. Yan, Q. Zhu, Y. Qi, J. Xu, Y. Peng et al., Towards high-performance aqueous zinc batteries via a semi-conductive bipolar-type polymer cathode. Angew. Chem. Int. Ed. **61**(42), e202211107 (2022). 10.1002/anie.202211107 36050284

[77] S. Ting, H. Chengmin, H. Qi, S. Ziyang, L. Yaokang et al., Hydrogen bonding-stabilized bipolar organic cathode achieved all-round enhancement in zinc batteries. Chem. Eng. J. **500**, 157627 (2024). 10.1016/j.cej.2024.157627

[78] Z. Song, L. Miao, H. Duan, Y. Lv, L. Gan et al., Multielectron redox-bipolar tetranitroporphyrin macrocycle cathode for high-performance zinc-organic batteries. Angew. Chem. Int. Ed. **63**(16), e202401049 (2024). 10.1002/anie.202401049 38372434

[79] L.-W. Luo, S. Zhu, W. Ma, D. Li, C. Zhang et al., A donor-acceptor conjugated bipolar polymer with multielectron redox sites for long-cycle-life and high-rate aqueous zinc dual-ion batteries. J. Energy Chem. **102**, 144–150 (2025). 10.1016/j.jechem.2024.10.042

[80] Y. Zou, T. Liu, Q. Du, Y. Li, H. Yi et al., A four-electron Zn-I_2_ aqueous battery enabled by reversible I^−^/I_2_/I^+^ conversion. Nat. Commun. **12**(1), 170 (2021). 10.1038/s41467-020-20331-9 PMC7794333 33419999

[81] S. Bi, H. Wang, Y. Zhang, M. Yang, Q. Li et al., Six-electron-redox iodine electrodes for high-energy aqueous batteries. Angew. Chem. Int. Ed. **62**(49), e202312982 (2023). 10.1002/anie.202312982 37861096

[82] W. Li, H. Xu, H. Zhang, F. Wei, T. Zhang et al., Designing ternary hydrated eutectic electrolyte capable of four-electron conversion for advanced Zn–I_2_ full batteries. Energy Environ. Sci. **16**(10), 4502–4510 (2023). 10.1039/d3ee01567j

[83] J. Zhao, Y. Chen, M. Zhang, Z. An, B. Nian et al., Iodine/chlorine multi‐electron conversion realizes high energy density zinc‐iodine batteries. Adv. Sci. **12**(1), 2410988 (2024). 10.1002/advs.202410988 PMC11714215 39499723

[84] C.L.T. Liu, H. Wang, J. Li, P. Jiang et al., Aqueous electrolyte with weak hydrogen bonds for four-electron zinc–iodine battery operates in a wide temperature range. Adv. Mater. **36**(32), 2405473 (2024). 10.1002/adma.202405473 38837833

[85] M. Wang, Y. Meng, M. Sajid, Z. Xie, P. Tong et al., Bidentate coordination structure facilitates high-voltage and high-utilization aqueous Zn−I_2_ batteries. Angew. Chem. Int. Ed. **63**(39), e202404784 (2024). 10.1002/anie.202404784 38868978

[86] W. Zong, J. Li, C. Zhang, Y. Dai, Y. Ouyang et al., Dynamical janus interface design for reversible and fast-charging zinc–iodine battery under extreme operating conditions. J. Am. Chem. Soc. **146**(31), 21377–21388 (2024). 10.1021/jacs.4c03615 39046802

[87] P. Jiang, Q. Du, C. Lei, C. Xu, T. Liu et al., Stabilized four-electron aqueous zinc–iodine batteries by quaternary ammonium complexation. Chem. Sci. **15**(9), 3357–3364 (2024). 10.1039/d3sc06155h PMC10901522 38425523

[88] C. Wang, X. Ji, J. Liang, S. Zhao, X. Zhang et al., Activating and stabilizing a reversible four electron redox reaction of I^−^/I^+^ for aqueous Zn-iodine battery. Angew. Chem. Int. Ed. **63**(25), e202403187 (2024). 10.1002/anie.202403187 38501218

[89] B. Wanyan, Y. Zhan, Z. Miao, W. Guo, J. Wang et al., Energetic aqueous zinc-sulfur battery achieved via co-solvent and redox mediator synergistic regulation. Compos. B Eng. **284**, 111709 (2024). 10.1016/j.compositesb.2024.111709

[90] A. Amiri, K. Bashandeh, R. Sellers, L. Vaught, M. Naraghi et al., Fully integrated design of a stretchable kirigami-inspired micro-sized zinc–sulfur battery. J. Mater. Chem. A **11**(20), 10788–10797 (2023). 10.1039/d2ta08544e

[91] M. Cui, J. Fei, F. Mo, H. Lei, Y. Huang, Ultra-high-capacity and dendrite-free zinc–sulfur conversion batteries based on a low-cost deep eutectic solvent. ACS Appl. Mater. Interfaces **13**(46), 54981–54989 (2021). 10.1021/acsami.1c15750 34780154

[92] W. Zong, J. Li, C. Zhang, Y. Dai, Y. Ouyang et al., Dynamical janus interface design for reversible and fast-charging zinc-iodine battery under extreme operating conditions. J. Am. Chem. Soc. **146**(31), 21377–21388 (2024). 10.1021/jacs.4c03615 39046802

[93] H. Tian, G. Feng, Q. Wang, Z. Li, W. Zhang et al., Three-dimensional zn-based alloys for dendrite-free aqueous zn battery in dual-cation electrolytes. Nat. Commun. **13**(1), 7922 (2022). 10.1038/s41467-022-35618-2 PMC9789050 36564385

[94] T. Zhou, H. Wan, M. Liu, Q. Wu, Z. Fan et al., Regulating uniform nucleation of ZnS enables low-polarized and high stable aqueous Zn–S batteries. Mater. Today Energy **27**, 101025 (2022). 10.1016/j.mtener.2022.101025

[95] Z. Chen, Z. Huang, J. Zhu, D. Li, A. Chen et al., Highly reversible positive‐valence conversion of sulfur chemistry for high-voltage zinc-sulfur batteries. Adv. Mater. **36**(30), 2402898 (2024). 10.1002/adma.202402898 38862392

[96] T.S. Thomas, A.P. Sinha, D. Mandal, Modulating electrolyte solvation for high-performance aqueous zinc–sulfur batteries. J. Mater. Chem. A **12**(32), 21350–21356 (2024). 10.1039/d4ta03061c

[97] J. Liu, H. Wu, C. Ye, S.Z. Qiao, High-entropy sulfides catalyze rate-determining redox in fast-charging aqueous zinc-sulfur batteries. Angew. Chem. Int. Ed. **64**(28), e202503472 (2025). 10.1002/anie.202503472 PMC12232885 40320369

[98] X. Geng, X. Hou, X. He, H.J. Fan, Challenges and strategies on interphasial regulation for aqueous rechargeable batteries. Adv. Energy Mater. **14**(12), 2304094 (2024). 10.1002/aenm.202304094

[99] W. Li, K. Wang, K. Jiang, A low cost aqueous Zn–S battery realizing ultrahigh energy density. Adv. Sci. **7**(23), 2000761 (2020). 10.1002/advs.202000761 PMC7709974 33304742

[100] Y. Ding, L. Yin, T. Du, Y. Wang, Z. He et al., A trifunctional electrolyte enables aqueous zinc ion batteries with long cycling performance. Adv. Funct. Mater. **34**(30), 2314388 (2024). 10.1002/adfm.202314388

[101] H. Xia, G. Xu, X. Cao, C. Miao, H. Zhang et al., Single-ion-conducting hydrogel electrolytes based on slide-ring pseudo-polyrotaxane for ultralong-cycling flexible zinc-ion batteries. Adv. Mater. **35**(36), e2301996 (2023). 10.1002/adma.202301996 37339158

[102] Y. Ren, J. Li, Y. Zhang, Y. Huang, Z. Li, Trace selenium doping for improving the reaction kinetics of ZnS cathode for aqueous Zn–S batteries. Small **20**(38), 2402466 (2024). 10.1002/smll.202402466 38742945

[103] H. Zhang, Z. Shang, G. Luo, S. Jiao, R. Cao et al., Redox catalysis promoted activation of sulfur redox chemistry for energy-dense flexible solid-state Zn–S battery. ACS Nano **16**(5), 7344–7351 (2021). 10.1021/acsnano.1c08645 34889091

[104] I.R. Tay, J. Xue, W.S.V. Lee, Methods for characterizing intercalation in aqueous zinc ion battery cathodes: a review. Adv. Sci. **10**(26), e2303211 (2023). 10.1002/advs.202303211 PMC10502642 37424052

[105] H. Zhang, M. Yang, J. Xiao, Z. Wu, W. Xin et al., Internal electron donor accelerated sulfur redox for aqueous Zn–S batterie. Adv. Funct. Mater. **34**(41), 2406125 (2024). 10.1002/adfm.202406125

[106] X. Li, S. Wang, D. Zhang, P. Li, Z. Chen et al., Perovskite cathodes for aqueous and organic iodine batteries operating under one and two electrons redox modes. Adv. Mater. **36**(4), e2304557 (2024). 10.1002/adma.202304557 37587645

[107] Y. Zhang, A. Amardeep, Z. Wu, L. Tao, J. Xu et al., A tellurium-boosted high-areal-capacity zinc-sulfur battery. Adv. Sci. **11**(23), 2308580 (2024). 10.1002/advs.202308580 PMC11187902 38566441

[108] Y. Li, J. Zhao, Q. Hu, T. Hao, H. Cao et al., Prussian blue analogs cathodes for aqueous zinc ion batteries. Mater. Today Energy **29**, 101095 (2022). 10.1016/j.mtener.2022.101095

[109] L. Wang, N. Liu, Q. Li, X. Wang, J. Liu et al., Dual redox reactions of silver hexacyanoferrate prussian blue analogue enable superior electrochemical performance for Zinc-ion storage. Angew. Chem. Int. Ed. **64**(4), e202416392 (2024). 10.1002/anie.202416392 39401949

[110] Q. Yang, F. Mo, Z. Liu, L. Ma, X. Li et al., Activating C‐coordinated iron of iron hexacyanoferrate for Zn hybrid-ion batteries with 10 000-cycle lifespan and superior rate capability. Adv. Mater. **31**(32), 1901521 (2019). 10.1002/adma.201901521 31192499

[111] R. Trócoli, G. Kasiri, F. La Mantia, Phase transformation of copper hexacyanoferrate KCuFe(CN)_6_ during zinc insertion: effect of co-ion intercalation. J. Power. Sources **400**, 167–171 (2018). 10.1016/j.jpowsour.2018.08.015

[112] B. Purusottam Reddy, P. Reddy Prasad, K. Mallikarjuna, M. Chandra Sekhar, Y.-W. Lee et al., Mn–Co prussian blue analogue cubic frames for efficient aqueous Zn ion batteries. Micropor. Mesopor. Mater. **362**, 112793 (2023). 10.1016/j.micromeso.2023.112793

[113] G. Zhang, Y. Li, G. Du, J. Lu, Q. Wang et al., Spiral-concave prussian blue crystals with rich steps: growth mechanism and coordination regulation. Angew. Chem. Int. Ed. **64**(2), e202414650 (2025). 10.1002/anie.202414650 39206502

[114] W.A. Syed, A.K. Kakarla, H. Bandi, R. Shanthappa, J.S. Yu, Copper substituted manganese prussian blue analogue composite nanostructures for efficient aqueous Zinc-ion batteries. J. Energy Storage **99**, 113325 (2024). 10.1016/j.est.2024.113325

[115] H.-g Wang, Q. Wu, L. Cheng, G. Zhu, The emerging aqueous zinc-organic battery. Coord. Chem. Rev. **472**, 214772 (2022). 10.1016/j.ccr.2022.214772

[116] Y. Lu, Y. Cai, Q. Zhang, J. Chen, Insights into redox processes and correlated performance of organic carbonyl electrode materials in rechargeable batteries. Adv. Mater. **34**(22), 2104150 (2022). 10.1002/adma.202104150 34617334

[117] M. Yiyuan, Q. Qi, M. Qi, Y. Yuyang, L. Huijun et al., A small molecular cathode for high-performance calcium metal batteries. Adv. Funct. Mater. **35**, 2411715 (2024). 10.1002/adfm.202411715

[118] P. Poizot, J. Gaubicher, S. Renault, L. Dubois, Y. Liang et al., Opportunities and challenges for organic electrodes in electrochemical energy storage. Chem. Rev. **120**, 6490 (2020). 10.1021/acs.chemrev.9b00482 32207919

[119] Z. Li, J. Tan, Y. Wang, C. Gao, Y. Wang et al., Building better aqueous Zn-organic batteries. Energy Environ. Sci. **16**, 2398 (2023). 10.1039/d3ee00211j

[120] Z. Song, L. Miao, H. Duan, L. Ruhlmann, Y. Lv et al., Anionic co-insertion charge storage in dinitrobenzene cathodes for high-performance aqueous zinc–organic batteries. Angew. Chem. Int. Ed. **61**, e202208821 (2022). 10.1002/anie.202208821 35781762

[121] Z. Song, L. Miao, Y. Lv, L. Gan, M. Liu, NH_4_^+^ charge carrier coordinated H-bonded organic small molecule for fast and superstable rechargeable zinc batteries. Angew. Chem. Int. Ed. **135**, e202309446 (2023). 10.1002/anie.202309446 37507839

[122] Y. Zhang, M. Li, Z. Li, Y. Lu, H. Li et al., A high capacity p-type organic cathode material for aqueous zinc batteries. Angew. Chem. Int. Ed. **136**, e202410342 (2024). 10.1002/anie.202410342 39223696

[123] M.H. Lee, G. Kwon, H. Lim, J. Kim, S.J. Kim et al., High-energy and long-lasting organic electrode for a rechargeable aqueous battery. ACS Energy Lett. **7**, 3637 (2022). 10.1021/acsenergylett.2c01535

[124] D. Li, Y. Guo, C. Zhang, X. Chen, W. Zhang et al., Unveiling organic electrode materials in aqueous zinc-ion batteries: from structural design to electrochemical performance. Nano-Micro Lett. **16**(1), 194 (2024). 10.1007/s40820-024-01404-6 PMC11093963 38743294

[125] J. Kim, Y. Kim, J. Yoo, G. Kwon, Y. Ko et al., Organic batteries for a greener rechargeable world. Nat. Rev. Mater. **8**, 54 (2022). 10.1038/s41578-022-00478-1

[126] N. Chen, W. Wang, Y. Ma, M. Chuai, X. Zheng et al., Aqueous zinc–chlorine battery modulated by a MnO redox adsorbent. Small Methods **8**(6), 2201553 (2023). 10.1002/smtd.202201553 37086122

[127] Y. Yin, Z. Yuan, X. Li, Rechargeable aqueous zinc–bromine batteries: an overview and future perspectives. Phys. Chem. Chem. Phys. **23**(46), 26070–26084 (2021). 10.1039/d1cp03987c 34787128

[128] H. Xu, W. Yang, M. Li, H. Liu, S. Gong et al., Advances in aqueous zinc ion batteries based on conversion mechanism: challenges, strategies, and prospects. Small **20**(27), 2310972 (2024). 10.1002/smll.202310972 38282180

[129] D. Lin, Y. Li, Recent advances of aqueous rechargeable zinc-iodine batteries: challenges, solutions, and prospects. Adv. Mater. **34**(23), e2108856 (2022). 10.1002/adma.202108856 35119150

[130] L. Yan, S. Zhang, Q. Kang, X. Meng, Z. Li et al., Iodine conversion chemistry in aqueous batteries: challenges, strategies, and perspectives. Energy Storage Mater. **54**, 339–365 (2023). 10.1016/j.ensm.2022.10.027

[131] W. Du, Q. Huang, X. Zheng, Y. Lv, L. Miao et al., High-conversion-efficiency and stable six-electron Zn–I_2_ batteries enabled by organic iodide/thiazole-linked covalent organic frameworks. Energy Environ. Sci. **18**(13), 6540–6547 (2025). 10.1039/d5ee00365b

[132] J.J.I.M. Kolthoff, Amperometric titration and voltammetric determination of iodide. Anal. Chem. **25**(12), 1833–1837 (1952). 10.1021/ac60084a012

[133] J.J. By, I.M. Kolthoff, Voltammetry of iodine and iodide at rotated platinum wire electrodes. J. Am. Chem. Soc. **75**(7), 1571–1575 (1952). 10.1021/ja01103a015

[134] Z. Jiang, X. Yang, J. Zhang, J. Yang, B. Sun et al., From conventional two-electron to emerging multi-electron zinc-iodine batteries: advantages, challenges, and future perspectives. Adv. Funct. Mater. **35**(50), e11754 (2025). 10.1002/adfm.202511754

[135] Y. Yang, S. Liang, B. Lu, J. Zhou, Eutectic electrolyte based on n-methylacetamide for highly reversible zinc–iodine battery. Energy Environ. Sci. **15**(3), 1192–1200 (2022). 10.1039/d1ee03268b

[136] Y. Zou, T. Liu, Q. Du, Y. Li, H. Yi et al., A four-electron Zn-I_2_ aqueous battery enabled by reversible I^-^/I_2_/I^+^ conversion. Nat. Commun. **12**(1), 170 (2021). 10.1038/s41467-020-20331-9 PMC7794333 33419999

[137] W. Ma, T. Liu, C. Xu, C. Lei, P. Jiang et al., A twelve-electron conversion iodine cathode enabled by interhalogen chemistry in aqueous solution. Nat. Commun. **14**(1), 5508 (2023). 10.1038/s41467-023-41071-6 PMC10484974 37679335

[138] C. Xie, C. Wang, Y. Xu, T. Li, Q. Fu et al., Reversible multielectron transfer I^−^/IO_3_^−^ cathode enabled by a hetero-halogen electrolyte for high-energy-density aqueous batteries. Nat. Energy **9**(6), 714–724 (2024). 10.1038/s41560-024-01515-9

[139] G. Raspi, F. Pergola, R. Guidelli, Electro-oxidation of iodine on smooth platinum in aqueous media. Anal. Chem. **44**(3), 472–479 (1972). 10.1021/ac60311a009 22309533

[140] Y. Chen, J. Ning, Y. Wen, K. Yao, Y. Zhang, Optimization strategies for high-performance aqueous zinc-sulfur batteries: challenges and future perspectives. Energy Mater. **5**(4), 500035 (2025). 10.20517/energymater.2024.123

[141] Y. Guo, X. Zhu, J. Zhang, T. Zhang, Z. Wang et al., Engineering electrolyte network structure for improved kinetics and dendrite suppression in zn-s batteries. Angew. Chem. Int. Ed. **64**(12), e202422047 (2025). 10.1002/anie.202422047 39601202

[142] D. Chao, C. Ye, F. Xie, W. Zhou, Q. Zhang et al., Atomic engineering catalyzed MnO_2_ electrolysis kinetics for a hybrid aqueous battery with high power and energy density. Adv. Mater. **32**(25), 2001894 (2020). 10.1002/adma.202001894 32424910

[143] D. Chao, W. Zhou, C. Ye, Q. Zhang, Y. Chen et al., An electrolytic Zn–MnO_2_ battery for high-voltage and scalable energy storage. Angew. Chem. Int. Ed. **58**(23), 7823–7828 (2019). 10.1002/anie.201904174 30972886

[144] Y. Li, N. Zhang, J.-Y. Wang, P. Wang, Z. Liu et al., Construction of high-voltage aqueous Zn-MnO_2_ batteries based on polar small-molecule organic acid-induced MnO_2_/Mn^2+^ reactions. Chem. Eng. J. **507**, 160415 (2025). 10.1016/j.cej.2025.160415

[145] X. Zeng, J. Liu, J. Mao, J. Hao, Z. Wang et al., Toward a reversible Mn^4+^/Mn^2+^ redox reaction and dendrite-free Zn anode in near-neutral aqueous Zn/MnO_2_ batteries via salt anion chemistry. Adv. Energy Mater. **10**(32), 1904163 (2020). 10.1002/aenm.201904163

[146] H. Lv, Y. Song, Z. Qin, M. Zhang, D. Yang et al., Disproportionation enabling reversible MnO_2_/Mn^2+^ transformation in a mild aqueous Zn- MnO_2_ hybrid battery. Chem. Eng. J. **430**(3), 133064 (2022). 10.1016/j.cej.2021.133064

[147] Y. Deng, H. Wang, M. Fan, B. Zhan, L.-J. Zuo et al., Nanomicellar electrolyte to control release ions and reconstruct hydrogen bonding network for ultrastable high-energy-density Zn–Mn battery. J. Am. Chem. Soc. **145**(36), 20109–20120 (2023). 10.1021/jacs.3c07764 37656940

[148] M. Xie, X. Zhang, R. Wang, Y. Jiao, Z. Shu et al., Mn-o bond engineering mitigating jahn-teller effects of manganese oxide for aqueous zinc-ion battery applications. Chem. Eng. J. **494**, 152908 (2024). 10.1016/j.cej.2024.152908

[149] Y. Liu, Y. Ma, Y. Zhang, Y. Li, J. Chen, L. Wang et al., A new design strategy enables high mn‐utilization rate in aqueous zinc–manganese batteries: constructing cathodic local mn‐rich region. Adv. Energy Mater. **14**(19), 2304161 (2024). 10.1002/aenm.202304161

[150] Y. Chen, S. Gu, S. Wu, X. Ma, I. Hussain et al., Copper activated near-full two-electron Mn^4+^/Mn^2+^ redox for mild aqueous Zn/MnO_2_ battery. Chem. Eng. J. **450**, 137923 (2022). 10.1016/j.cej.2022.137923

[151] Y. Liu, K. Wang, X. Yang, J. Liu, X.-X. Liu et al., Enhancing two-electron reaction contribution in MnO_2_ cathode material by structural engineering for stable cycling in aqueous zn batteries. ACS Nano **17**(15), 14792–14799 (2023). 10.1021/acsnano.3c02965 37459215

[152] Y. Zhao, X. Xia, Q. Li, Y. Wang, Y. Fan et al., Activating the redox chemistry of MnO_2_/Mn^2+^ in aqueous zn batteries based on multi-ions doping regulation. Energy Storage Mater. **67**, 103268 (2024). 10.1016/j.ensm.2024.103268

[153] Z. Shen, Z. Tang, C. Li, L. Luo, J. Pu et al., Precise proton redistribution for two-electron redox in aqueous zinc/manganese dioxide batteries. Adv. Energy Mater. **11**(41), 2102055 (2021). 10.1002/aenm.202102055

[154] X. Li, Z. Xu, Y. Qian, Z. Hou, In-situ regulated competitive proton intercalation and deposition/dissolution reaction of MnO_2_ for high-performance flexible zinc-manganese batteries. Energy Storage Mater. **53**, 72–78 (2022). 10.1016/j.ensm.2022.09.002

[155] Z. Liu, Y. Yang, B. Lu, S. Liang, H.J. Fan et al., Insights into complexing effects in acetate-based Zn-MnO_2_ batteries and performance enhancement by all-round strategies. Energy Storage Mater. **52**, 104–110 (2022). 10.1016/j.ensm.2022.07.043

[156] M. Tang, X. Zhao, R. Han, Y. Wang, Y. Ding et al., Electrolyte decoupling strategy for metal oxide-based zinc-ion batteries free of crosstalk effect. Angew. Chem. Int. Ed. **64**(11), e202421574 (2025). 10.1002/anie.202421574 39754524

[157] Y. Zhao, R. Zhou, Z. Song, X. Zhang, T. Zhang et al., Interfacial designing of MnO_2_ half-wrapped by aromatic polymers for high-performance aqueous zinc-ion batteries. Angew. Chem. Int. Ed. **61**(49), e202212231 (2022). 10.1002/anie.202212231 36239266

[158] L. Dai, Y. Wang, L. Sun, Y. Ding, Y. Yao et al., Jahn–teller distortion induced Mn^2+^‐rich cathode enables optimal flexible aqueous high‐voltage Zn‐Mn batteries. Adv. Sci. **8**(12), 2004995 (2021). 10.1002/advs.202004995 PMC8224442 34194938

[159] X. Xia, Y. Zhao, Y. Zhao, M. Xu, W. Liu et al., Mo doping provokes two electron reaction in MnO_2_ with ultrahigh capacity for aqueous zinc ion batteries. Nano Res. **16**(2), 2511–2518 (2022). 10.1007/s12274-022-5057-0

[160] X. Gao, C. Shen, H. Dong, Y. Dai, P. Jiang et al., Co-intercalation strategy for simultaneously boosting two-electron conversion and bulk stabilization of mn-based cathodes in aqueous zinc-ion batteries. Energy Environ. Sci. **17**(6), 2287–2297 (2024). 10.1039/d3ee04492k

[161] M. Chuai, J. Yang, M. Wang, Y. Yuan, Z. Liu et al., High-performance Zn battery with transition metal ions co-regulated electrolytic MnO_2_. eScience **1**(2), 178–185 (2021). 10.1016/j.esci.2021.11.002

[162] H. Ye, X. Zeng, X. Li, K. He, Y. Li et al., Review of ion doping and intercalation strategies for advancing manganese-based oxide cathodes in aqueous zinc-ion batteries. Nano Energy **136**, 110740 (2025). 10.1016/j.nanoen.2025.110740

[163] Y. Fan, Q. Wang, Y. Xie, N. Zhou, Y. Yang et al., Advances in aqueous zinc-ion battery systems: cathode materials and chemistry. Prog. Mater. Sci. **149**, 101393 (2025). 10.1016/j.pmatsci.2024.101393

[164] Y. Liu, Y. Zhang, H. Jiang, J. Sun, Z. Feng et al., Synergistic engineering of oxygen-defect and heterojunction boosts Zn^2+^ (de)intercalation kinetics in vanadium oxide for high-performance zinc-ion batteries. Chem. Eng. J. **435**, 134949 (2022). 10.1016/j.cej.2022.134949

[165] X. Hu, S. Gao, T. Lin, X. Peng, Y. Huang et al., Iodine-doped sodium vanadate cathode for improved Zn ion diffusion kinetics. Adv. Mater. **37**(46), 2416714 (2025). 10.1002/adma.202416714 PMC12631527 39998260

[166] R. Sinha, X. Xie, Y. Yang, Y. Li, Y. Xue et al., Failure mechanisms and strategies for vanadium oxide-based cathode in aqueous zinc batteries. Adv. Energy Mater. **15**(14), 2404815 (2025). 10.1002/aenm.202404815

[167] P. He, M. Yan, X. Liao, Y. Luo, L. Mai et al., Reversible V^3+^/V^5+^ double redox in lithium vanadium oxide cathode for zinc storage. Energy Storage Mater. **29**, 113–120 (2020). 10.1016/j.ensm.2020.04.005

[168] D. Chen, M. Lu, B. Wang, R. Chai, L. Li et al., Uncover the mystery of high-performance aqueous zinc-ion batteries constructed by oxygen-doped vanadium nitride cathode: cationic conversion reaction works. Energy Storage Mater. **35**, 679–686 (2021). 10.1016/j.ensm.2020.12.001

[169] Y. Zhang, S. Jiang, Y. Li, X. Ren, P. Zhang et al., In situ grown hierarchical electrospun nanofiber skeletons with embedded vanadium nitride nanograins for ultra-fast and super-long cycle life aqueous Zn-ion batteries. Adv. Energy Mater. **13**(5), 2202826 (2022). 10.1002/aenm.202202826

[170] P. Luo, W. Zhong, W. Zhang, M. Zhou, W. Tang et al., Trigger a multi-electron reaction by tailoring electronic structure of VO_2_ toward more efficient aqueous zinc metal batteries. J. Colloid Interface Sci. **666**, 371–379 (2024). 10.1016/j.jcis.2024.04.020 38603879

[171] K. Wang, H. Li, G. Guo, L. Zheng, S. Passerini et al., Enabling multi-electron reactions in NASICON positive electrodes for aqueous zinc-metal batteries. ACS Energy Lett. **8**(4), 1671–1679 (2023). 10.1021/acsenergylett.2c02837

[172] S. Li, X. Xu, W. Chen, J. Zhao, K. Wang et al., Synergetic impact of oxygen and vanadium defects endows NH_4_V_4_O_10_ cathode with superior performances for aqueous zinc-ion battery. Energy Storage Mater. **65**, 103108 (2024). 10.1016/j.ensm.2023.103108

[173] J. Cao, D. Zhang, Y. Yue, T. Pakornchote, T. Bovornratanaraks et al., Revealing the impacts of oxygen defects on Zn^2+^ storage performance in V_2_O_5_. Mater. Today Energy. **21**, 100824 (2021). 10.1016/j.mtener.2021.100824

[174] Y. Dai, C. Zhang, J. Li, X. Gao, P. Hu et al., Inhibition of vanadium cathodes dissolution in aqueous Zn-ion batteries. Adv. Mater. **36**(14), e2310645 (2024). 10.1002/adma.202310645 PMC11475447 38226766

[175] Y. Shi, B. Yang, G. Song, Z. Chen, M. Shakouri et al., Ambient synthesis of vanadium-based prussian blue analogues nanocubes for high-performance and durable aqueous zinc-ion batteries with eutectic electrolytes. Angew. Chem. Int. Ed. **63**(45), e202411579 (2024). 10.1002/anie.202411579 39086196

[176] Y. Zeng, X.F. Lu, S.L. Zhang, D. Luan, S. Li et al., Construction of Co-Mn prussian blue analog hollow spheres for efficient aqueous Zn-ion batteries. Angew. Chem. Int. Ed. **60**(41), 22189–22194 (2021). 10.1002/anie.202107697 PMC8518934 34313363

[177] Y. Zeng, J. Xu, Y. Wang, S. Li, D. Luan et al., Formation of cumn prussian blue analog double-shelled nanoboxes toward long-life Zn-ion batteries. Angew. Chem. Int. Ed. (2022). 10.1002/anie.202212031 36177990

[178] Y. Tan, H. Yang, C. Miao, Y. Zhang, D. Chen et al., Hydroxylation strategy unlocking multi-redox reaction of manganese hexacyanoferrate for aqueous zinc-ion battery. Chem. Eng. J. **457**, 141323 (2023). 10.1016/j.cej.2023.141323

[179] Y. Zhang, Y. Wang, L. Lu, C. Sun, D.Y.W. Yu, Vanadium hexacyanoferrate with two redox active sites as cathode material for aqueous Zn-ion batteries. J. Power. Sources **484**, 229263 (2021). 10.1016/j.jpowsour.2020.229263

[180] H. Ma, R. Chen, B. Liu, J. Yan, G. Wang et al., Synthesis of ultrasmall vanadium ferricyanide nanocrystallines with the aidance of graphene self-assembled fibers towards reinforced zinc storage performance. Chem. Eng. J. **489**, 151112 (2024). 10.1016/j.cej.2024.151112

[181] P.P. Puthiyaveetil, A. Nair, S. Dilwale, M. Kurian, K. Joshi et al., Insights on prussian blue analogue cathode material engineered with polypyrrole surface protection layer for aqueous rechargeable zinc metal battery. Small **21**(4), 2409947 (2024). 10.1002/smll.202409947 39588842

[182] G. Yang, Z. Liang, Q. Li, Y. Li, F. Tian et al., Epitaxial core–shell mnfe prussian blue cathode for highly stable aqueous zinc batteries. ACS Energy Lett. **8**(10), 4085–4095 (2023). 10.1021/acsenergylett.3c01603

[183] Z. Wang, J. Ren, G. Ling, J. Guo, Y. Lv et al., Prussian blue-derived atomic Fe/Fe_3_C@N-doped c catalysts supported by carbon cloth as integrated air cathode for flexible Zn-air batteries. Adv. Sci. **12**(1), e2407631 (2025). 10.1002/advs.202407631 PMC11714216 39467100

[184] S. Bian, Y. Yang, S. Liu, F. Ye, H. Tang et al., Recent progress of the cathode material design for aqueous zn-organic batteries. Chemistry **30**(13), e202303917 (2024). 10.1002/chem.202303917 38093171

[185] X. Peng, A. Baktash, N. Alghamdi, M.M. Rana, Y. Huang et al., Boosting aluminum storage in highly stable covalent organic frameworks with abundant accessible carbonyl groups. Adv. Energy Mater. **14**(22), 2400147 (2024). 10.1002/aenm.202400147

[186] X. Peng, A. Baktash, Y. Huang, N. Alghamdi, J. You et al., Multi-redox covalent organic frameworks for aluminium organic batteries. Energy Storage Mater. **71**, 103674 (2024). 10.1016/j.ensm.2024.103674

[187] Y. Pengshu, L. Zhiheng, M. Longli, F. Bingjian, L. Zhu et al., Eco-friendly high-performance symmetric all-COF/graphene aqueous zinc-ion batteries. Adv. Mater. **36**(52), 2414379 (2024). 10.1002/adma.202414379 39544151

[188] L. Bai, Z. Hu, C. Hu, S. Zhang, Y. Ying et al., Utilizing cationic vacancies and spontaneous polarization on cathode to enhance zinc-ion storage and inhibit dendrite growth in zinc-ion batteries. Angew. Chem. Int. Ed. **62**(26), e202301631 (2023). 10.1002/anie.202301631 37017994

[189] X. Ma, X. Cao, M. Yao, L. Shan, X. Shi et al., Organic–inorganic hybrid cathode with dual energy-storage mechanism for ultrahigh-rate and ultralong-life aqueous zinc-ion batteries. Adv. Mater. **34**(6), 2105452 (2022). 10.1002/adma.202105452 34786778

[190] Y. Zhao, D. Wang, X. Li, Q. Yang, Y. Guo et al., Initiating a reversible aqueous Zn/sulfur battery through a “liquid film.” Adv. Mater. **32**(32), 2003070 (2020). 10.1002/adma.202003070 32596928

[191] Y. Wang, C. Wang, Z. Ni, Y. Gu, B. Wang et al., Binding zinc ions by carboxyl groups from adjacent molecules toward long-life aqueous zinc-organic batteries. Adv. Mater. **32**(16), 2000338 (2020). 10.1002/adma.202000338 32141139

[192] Y. Zhao, Y. Huang, F. Wu, R. Chen, L. Li, High-performance aqueous zinc batteries based on organic/organic cathodes integrating multiredox centers. Adv. Mater. **33**(52), 2106469 (2021). 10.1002/adma.202106469 34625999

[193] M. Zhao, Y. Lv, S. Zhao, Y. Xiao, J. Niu et al., Simultaneously stabilizing both electrodes and electrolytes by a self-separating organometallics interface for high-performance zinc-ion batteries at wide temperatures. Adv. Mater. **34**(49), 2206239 (2022). 10.1002/adma.202206239 36255143

[194] S.N. Bariya, K. Surana, Y.G. Kapdi, H.K. Machhi, J. Prasad et al., Ultrafast self-charging in acid–base free aqueous rechargeable zinc–quinhydrone batteries. Adv. Energy Mater. **14**(45), 2401257 (2024). 10.1002/aenm.202401257

[195] C. Wang, T. He, J. Cheng, Q. Guan, B. Wang, Bioinspired interface design of sewable, weavable, and washable fiber zinc batteries for wearable power textiles. Adv. Funct. Mater. **30**(42), 2004430 (2020). 10.1002/adfm.202004430

[196] Z. Song, L. Miao, Y. Lv, L. Gan, M. Liu, NH^4+^ charge carrier coordinated H-bonded organic small molecule for fast and superstable rechargeable zinc batteries. Angew. Chem. Int. Ed. **62**(38), e202309446 (2023). 10.1002/anie.202309446 37507839

[197] B. Li, Y. Zeng, W. Zhang, B. Lu, Q. Yang et al., Separator designs for aqueous zinc-ion batteries. Sci. Bull. **69**(5), 688–703 (2024). 10.1016/j.scib.2024.01.011 38238207

[198] W. Li, H. Xu, H. Zhang, F. Wei, L. Huang et al., Tuning electron delocalization of hydrogen-bonded organic framework cathode for high-performance zinc-organic batteries. Nat. Commun. **14**(1), 5235 (2023). 10.1038/s41467-023-40969-5 PMC10462634 37640714

[199] T. Wirtanen, T. Prenzel, J.-P. Tessonnier, S.R. Waldvogel, Cathodic corrosion of metal electrodes—how to prevent it in electroorganic synthesis. Chem. Rev. **121**(17), 10241–10270 (2021). 10.1021/acs.chemrev.1c00148 PMC8431381 34228450

[200] H. Ma, J. Yu, M. Chen, X. Han, J. Chen et al., Amino‐enabled desolvation sieving effect realizes dendrite‐inhibiting thin separator for durable aqueous zinc‐ion batteries. Adv. Funct. Mater. **33**(52), 2307384 (2023). 10.1002/adfm.202307384

[201] H.-Y. Shi, Q. Jiang, W. Wu, Z. Lin, Z. Jia et al., Assisting zn storage in layered vanadyl phosphate cathode by interactions with oligoaniline pillars for rechargeable aqueous zinc batteries. Chem. Eng. J. **454**(3), 140323 (2023). 10.1016/j.cej.2022.140323

[202] T. Sun, Z. Yi, W. Zhang, Q. Nian, H.J. Fan et al., Dynamic balance of partial charge for small organic compound in aqueous zinc-organic battery. Adv. Funct. Mater. (2023). 10.1002/adfm.202306675

[203] W. Donghong, S. Qiwang, C. Xianjia, Q. Mengxuan, Z. Changyou et al., Organic nitro compounds for batteries. Adv. Funct. Mater. **35**(9), 2416000 (2024). 10.1002/adfm.202416000

[204] B. Li, K. Kumar, I. Roy, A.V. Morozov, O.V. Emelyanova et al., Capturing dynamic ligand-to-metal charge transfer with a long-lived cationic intermediate for anionic redox. Nat. Mater. **21**, 1165–1174 (2022). 10.1038/s41563-022-01278-2 35725928

[205] J. Huang, Z. Wang, M. Hou, X. Dong, Y. Liu et al., Polyaniline-intercalated manganese dioxide nanolayers as a high-performance cathode material for an aqueous zinc-ion battery. Nat. Commun. **9**(1), 2906 (2018). 10.1038/s41467-018-04949-4 PMC6060179 30046036

[206] S. Chen, K. Li, K.S. Hui, J. Zhang, Regulation of lamellar structure of vanadium oxide via polyaniline intercalation for high‐performance aqueous zinc-ion battery. Adv. Funct. Mater. **30**(43), 2003890 (2020). 10.1002/adfm.202003890

[207] X. Zhou, Z. Li, W. Li, X. Li, J. Fu et al., Regulating Na-ion solvation in quasi-solid electrolyte to stabilize na metal anode. Adv. Funct. Mater. **33**(11), 2212866 (2022). 10.1002/adfm.202212866

[208] X. Lai, Y. Li, J. Niu, J. Li, Y. Cao et al., Simultaneous electrodeposition of vanadium oxide and polyaniline derivatives and its application in aqueous zinc ion storage. Chem. Eng. J. **509**, 161447 (2025). 10.1016/j.cej.2025.161447

[209] Z. Pan, D.J.L. Brett, G. He, I.P. Parkin, Progress and perspectives of organosulfur for lithium–sulfur batteries. Adv. Energy Mater. **12**(8), 2103483 (2022). 10.1002/aenm.202103483

[210] H. Li, Y. Zhou, M. Zhao, B. Jin, Z. Wen et al., Suppressed shuttle via inhibiting the formation of long-chain lithium polysulfides and functional separator for greatly improved lithium-organosulfur batteries performance. Adv. Energy Mater. **10**(3), 1902695 (2019). 10.1002/aenm.201902695

[211] S. Tang, Q. Chen, Y. Si, W. Guo, B. Mao et al., Size effect of organosulfur and in situ formed oligomers enables high-utilization Na–organosulfur batteries. Adv. Mater. **33**(33), 2100824 (2021). 10.1002/adma.202100824 34247431

[212] P. Sang, Q. Chen, D.-Y. Wang, W. Guo, Y. Fu, Organosulfur materials for rechargeable batteries: structure, mechanism, and application. Chem. Rev. **123**(4), 1262–1326 (2023). 10.1021/acs.chemrev.2c00739 36757873

[213] R. Zou, W. Liu, F. Ran, Sulfur-containing polymer cathode materials: from energy storage mechanism to energy density. Infomat **4**(8), e12319 (2022). 10.1002/inf2.12319

[214] Q. Fan, J. Zhang, S. Fan, B. Xi, Z. Gao et al., Advances in functional organosulfur-based mediators for regulating performance of lithium metal batteries. Adv. Mater. **36**(45), 2409521 (2024). 10.1002/adma.202409521 39246200

[215] S. Haldar, P. Bhauriyal, A.R. Ramuglia, A.H. Khan, S. De Kock et al., Sulfide-bridged covalent quinoxaline frameworks for lithium–organosulfide batteries. Adv. Mater. **35**(16), 2210151 (2023). 10.1002/adma.202210151 36719245

[216] Z. Rong, L. Wenwu, R. Fen, A new thiol-sulfur click chemistry for lithium-organosulfide batteries. Innovation **6**, 100765 (2025). 10.1016/j.xinn.2024.100765 PMC11846034 39991483

[217] R. Yan, T. Ma, M. Cheng, X. Tao, Z. Yang et al., Metal–organic-framework-derived nanostructures as multifaceted electrodes in metal–sulfur batteries. Adv. Mater. **33**(27), 2008784 (2021). 10.1002/adma.202008784 PMC11468141 34031929

[218] X. Cheng, Z. Xiang, C. Yang, Y. Li, L. Wang et al., Polar organic molecules inserted in vanadium oxide with enhanced reaction kinetics for promoting aqueous zinc‐ion storage. Adv. Funct. Mater. **34**(9), 2311412 (2023). 10.1002/adfm.202311412

[219] X. Zhang, K. Chen, Z. Sun, G. Hu, R. Xiao et al., Structure-related electrochemical performance of organosulfur compounds for lithium–sulfur batteries. Energy Environ. Sci. **13**, 1076–1095 (2020). 10.1039/c9ee03848e

[220] W. Li, H. Xu, S. Ke, H. Zhang, H. Chen et al., Integrating electric ambipolar effect for high-performance zinc bromide batteries. Nano-Micro Lett. **17**(1), 143 (2025). 10.1007/s40820-024-01636-6 PMC11825431 39945958

[221] J. Chen, H. Yin, Q. Xue, J. Zhang, X. Chen et al., Design and synthesis of d-a polymer as cathode material for na-based dual-ion batteries with excellent cycling performance. Adv. Funct. Mater. **34**(52), 2411362 (2024). 10.1002/adfm.202411362

[222] Q. Xu, Z. Liu, Y. Jin, X. Yang, T. Sun et al., A bipolar-type covalent organic framework on carbon nanotubes with enhanced density of redox-active sites for high-performance lithium-ion batteries. Energy Environ. Sci. **17**, 5451–5460 (2024). 10.1039/d4ee00520a

[223] H. Wang, G. Liu, W. Zhou, Y. Wang, X. Dong, High-potential and stable organic cathode for rechargeable batteries with fast-charging and wide-temperature adaptability. Angew. Chem. Int. Ed. **64**(4), e202416874 (2025). 10.1002/anie.202416874 39489693

[224] S.J. Zhang, J. Hao, H. Wu, C.C. Kao, Q. Chen et al., Toward high-energy-density aqueous zinc-iodine batteries: multielectron pathways. ACS Nano **18**(42), 28557–28574 (2024). 10.1021/acsnano.4c10901 39383309

[225] J. Zhao, Y. Chen, M. Zhang, Z. An, B. Nian et al., Iodine/chlorine multi-electron conversion realizes high energy density zinc-iodine batteries. Adv. Sci. **12**(1), e2410988 (2025). 10.1002/advs.202410988 PMC11714215 39499723

[226] Y. Liu, L. Zhang, L. Liu, Q. Ma, R. Wang et al., All-climate energy-dense cascade aqueous Zn-I_2_ batteries enabled by a polycationic hydrogel electrolyte. Adv. Mater. **37**(46), 2415979 (2025). 10.1002/adma.202415979 39955652

[227] J. Kang, C. Wang, Z. Liu, L. Wang, Y. Meng et al., Electron-outflowing heterostructure hosts for high-voltage aqueous zinc-iodine batteries. Energy Storage Mater. **68**, 103367 (2024). 10.1016/j.ensm.2024.103367

[228] W.J. Ma, J.Y. Li, H.J. Wang, C.J. Lei, X. Liang, High efficiency alkaline iodine batteries with multi-electron transfer enabled by bi/bio redox mediator. Angew. Chem. Int. Ed. **63**(52), e202410994 (2024). 10.1002/anie.202410994 39381925

[229] Y. Guo, G.J.H. Lim, V. Verma, Y. Cai, K.K. Chan et al., Recent progress in zinc sulfur batteries: mechanism, challenges, and perspectives. Chem. Eng. J. **498**, 155329 (2024). 10.1016/j.cej.2024.155329

[230] X. Guo, H. Xu, Y. Tang, Z. Yang, F. Dou et al., Confining iodine into metal-organic framework derived metal-nitrogen-carbon for long-life aqueous zinc-iodine batteries. Adv. Mater. **36**(38), e2408317 (2024). 10.1002/adma.202408317 39081106

[231] P. Jiang, T. Liu, C. Lei, H. Wang, J. Li et al., Energetic hypervalent organoiodine electrochemistry for aqueous zinc batteries. J. Am. Chem. Soc. **146**(36), 25108–25117 (2024). 10.1021/jacs.4c08145 39190645

[232] J. Li, J. Cong, Y. Ren, H. Ji, Z. Li et al., Constructing a raincoat-like protective layer on sulfur cathode for aqueous Zn–S batteries. Energy Storage Mater. **70**, 103541 (2024). 10.1016/j.ensm.2024.103541

[233] T. Jin, X.-Y. Li, M. Zhao, S. Feng, Z. Li et al., Promoting the rate performances of weakly solvating electrolyte-based lithium‒sulfur batteries. Angew. Chem. Int. Ed. **64**(32), e202504898 (2025). 10.1002/anie.202504898 40458931

[234] X.-Y. Li, B.-Q. Li, T. Jin, S. Feng, Y.-C. Gao et al., Competitive anion coordination overcomes charge-transfer barriers for lithium–sulfur batteries. Joule **10**(2), 102259 (2026). 10.1016/j.joule.2025.102259

[235] Y.-W. Song, J. Zhou, Z.-X. Chen, J.-D. Zhang, L. Shen et al., Reducing the cathode thiele modulus to promote the discharge capacity of lithium–sulfur batteries. J. Energy Chem. **106**, 993–1001 (2025). 10.1016/j.jechem.2025.04.075

[236] Z.-X. Chen, J.-J. Zhao, G.-Y. Fang, F. Sun, M. Zhao et al., Sufficient cathode infiltration for stable 500 wh kg^−1^ level lithium–sulfur batteries. J. Energy Chem. **109**, 129–137 (2025). 10.1016/j.jechem.2025.05.006

[237] S. Mehta, S. Kaur, M. Singh, M. Kumar, K. Kumar et al., Unleashing ultrahigh capacity and lasting stability: Aqueous zinc-sulfur batteries. Adv. Energy Mater. **14**(27), 2401515 (2024). 10.1002/aenm.202401515

[238] P. Hei, Y. Sai, C. Liu, W. Li, J. Wang et al., Facilitating the electrochemical oxidation of ZnS through iodide catalysis for aqueous zinc-sulfur batteries. Angew. Chem. Int. Ed. **63**(9), e202316082 (2024). 10.1002/anie.202316082 38196064

[239] W. Wu, S. Wang, L. Lin, H.-Y. Shi, X. Sun, A dual-mediator for a sulfur cathode approaching theoretical capacity with low overpotential in aqueous Zn–S batteries. Energy Environ. Sci. **16**(10), 4326–4333 (2023). 10.1039/d3ee01749d

[240] S. Zhao, X. Wu, J. Zhang, C. Li, Z. Cui et al., Biomass-derived porous carbon with single-atomic cobalt toward high-performance aqueous zinc-sulfur batteries at room temperature. J. Energy Chem. **95**, 325–335 (2024). 10.1016/j.jechem.2024.03.054

[241] X. Ding, W. Liu, J. Zhao, L. Wang, Z. Zou, Photothermal CO_2_ catalysis towards the synthesis of solar fuel: from material and reactor engineering to techno-economic analysis. Adv. Mater. **37**(2), 2312093 (2024). 10.1002/adma.202312093 38683953

[242] Y. Guo, R. Chua, Y. Chen, Y. Cai, E.J.J. Tang et al., Hybrid electrolyte design for high-performance zinc-sulfur battery. Small **19**(29), 2207133 (2023). 10.1002/smll.202207133 36971296

[243] G. Chang, J. Liu, Y. Hao, C. Huang, Y. Yang et al., Bifunctional electrolyte additive with redox mediation and capacity contribution for sulfur cathode in aqueous Zn-S batteries. Chem. Eng. J. **457**, 141083 (2023). 10.1016/j.cej.2022.141083

[244] A. Zhang, T. Chen, R. Zhao, Y. Wang, J. Yang et al., Tunable organic-inorganic p-pi-d electron conjugation triggers d-pi hybridization in quinonized MnO_2_ superlattice toward ultrastable and high-rate Zn-MnO_2_ batteries. Angew. Chem. Int. Ed. **64**(14), e202423824 (2025). 10.1002/anie.202423824 39829039

[245] H. Wen, B. Yin, H. Wen, Y. Sun, J. Li et al., Synergistic strategy of targeted capture and potential responsive release for high-performance zinc-iodine batteries. Adv. Mater. **38**(9), e202516182 (2026). 10.1002/adma.202516182 41388718

[246] M. Yang, J. Zhu, M. Lai, P. Chen, Y. Lin et al., Discovery of D-band center engineered amorphous cathode with ultrahigh, superfast, and wide-temperature Zn^2+^ storage capability. Adv. Mater. **38**(21), e202520708 (2026). 10.1002/adma.202520708 41340329

[247] Y. Tan, W. Xu, F. Yang, J. Tao, D. Li et al., Dynamic polyiodide-trapping and proton-capturing dual-network engineering for high-areal-capacity, long-cycling and high-temperature Zn–I_2_ batteries. Adv. Mater. **38**(12), e202522609 (2026). 10.1002/adma.202522609 41524475

[248] Z. Lian, W. Yang, Z. Wu, L. Zhong, Z. Liu et al., A diffusion-retarded strategy for practical Zn–I_2_ batteries under harsh conditions. Angew. Chem. Int. Ed. **65**(4), e202515756 (2026). 10.1002/anie.202515756 41355445

[249] Z. Song, L. Miao, H. Duan, L. Ruhlmann, Y. Lv et al., Anionic co-insertion charge storage in dinitrobenzene cathodes for high-performance aqueous zinc-organic batteries. Angew. Chem. Int. Ed. **61**(35), e202208821 (2022). 10.1002/anie.202208821 35781762

[250] Q. Zong, Y. Zhuang, C. Liu, Q. Kang, Y. Wu et al., Dual effects of metal and organic ions co‐intercalation boosting the kinetics and stability of hydrated vanadate cathodes for aqueous zinc‐ion batteries. Adv. Energy Mater. **13**(31), 2301480 (2023). 10.1002/aenm.202301480

[251] Y. Liang, M. Qiu, P. Sun, W. Mai, Comprehensive review of electrolyte modification strategies for stabilizing Zn metal anodes. Adv. Funct. Mater. **33**(51), 2304878 (2023). 10.1002/adfm.202304878

[252] M.S. Javed, H. Lei, Z. Wang, B.-t Liu, X. Cai et al., 2D V_2_O_5_ nanosheets as a binder-free high-energy cathode for ultrafast aqueous and flexible Zn-ion batteries. Nano Energy **70**, 104573 (2020). 10.1016/j.nanoen.2020.104573

[253] S. Tang, C. Ye, X. Zhou, An azo polymer with abundant active sites and extended conjugation as a stable cathode for high-performance zinc-organic batteries. Angew. Chem. Int. Ed. **64**(17), e202501743 (2025). 10.1002/anie.202501743 39953932

[254] Z. Chen, Z. Huang, J. Zhu, D. Li, A. Chen et al., Highly reversible positive-valence conversion of sulfur chemistry for high-voltage zinc-sulfur batteries. Adv. Mater. **36**(30), e2402898 (2024). 10.1002/adma.202402898 38862392

[255] Z. Yan, J. Li, H. Liu, H. Zhang, S. Xi et al., A reversible six-electron transfer cathode for advanced aqueous zinc batteries. Angew. Chem. Int. Ed. **62**(47), e202312000 (2023). 10.1002/anie.202312000 37753789

[256] S. Liu, J. Li, W. Xiao, R. Chen, Z. Sun et al., Buried interface molecular hybrid for inverted perovskite solar cells. Nature **632**(8025), 536–542 (2024). 10.1038/s41586-024-07723-3 38925147

[257] C. Li, L. Hu, X. Ren, L. Lin, C. Zhan et al., Asymmetric charge distribution of active centers in small molecule quinone cathode boosts high-energy and high-rate aqueous zinc-organic batteries. Adv. Funct. Mater. **34**(16), 2313241 (2024). 10.1002/adfm.202313241

[258] Z. Fan, J. Wang, Y. Wu, X. Yan, D. Dai et al., Research progresses on cathode materials of aqueous zinc-ion batteries. J. Energy Chem. **97**, 237–264 (2024). 10.1016/j.jechem.2024.05.033

[259] L.F. Zhou, J.Y. Li, J. Peng, L.Y. Liu, H. Zhang et al., Advanced characterization techniques for phosphate cathodes in aqueous rechargeable zinc-based batteries. Carbon Energy **6**(10), e611 (2024). 10.1002/cey2.611

[260] Q. Zhang, S. Ju, S. Zhang, S. Xu, Z. Zhang, A multi-colored, structure-tolerant vanadate cathode for high-performance aqueous zinc-ion batteries. Adv. Energy Mater. **15**(18), 2404597 (2025). 10.1002/aenm.202404597

[261] T. Zhou, L. Xie, Q. Han, X. Qiu, Y. Xiao et al., Progress and prospect of vanadates as aqueous Zn-ion batteries cathodes. Coord. Chem. Rev. **498**, 215461 (2024). 10.1016/j.ccr.2023.215461

[262] J. Wang, C. Li, S. Zhao, R. Xia, Q. Zhang et al., Unlocking double redox reaction in conductive copper-organic framework for stable zinc-ion batteries. Chem. Eng. J. **504**, 158492 (2025). 10.1016/j.cej.2024.158492

[263] W. Zhong, R. Zhao, Y. Zhu, Y. Xu, W. Chen et al., Vacancy engineering on mnse cathode enables high‐rate and stable zinc-ion storage. Adv. Funct. Mater. **35**(17), 2419720 (2024). 10.1002/adfm.202419720

[264] M. Li, M. Liu, Y. Lu, G. Zhang, Y. Zhang et al., A dual active site organic–inorganic poly(o-phenylenediamine)/NH_4_V_3_O_8_ composite cathode material for aqueous zinc-ion batteries. Adv. Funct. Mater. **34**(19), 2312789 (2024). 10.1002/adfm.202312789

[265] S. Li, J. Shang, M. Li, M. Xu, F. Zeng et al., Design and synthesis of a pi-conjugated n-heteroaromatic material for aqueous zinc-organic batteries with ultrahigh rate and extremely long life. Adv. Mater. **35**(50), e2207115 (2023). 10.1002/adma.202207115 36177698

[266] W. Mantele, E. Deniz, UV-vis absorption spectroscopy: Lambert-beer reloaded. Spectrochim. Acta A Mol. Biomol. Spectrosc. **173**, 965–968 (2017). 10.1016/j.saa.2016.09.037 27727137

[267] X. Wang, H. Peng, H. Zheng, Z. Liu, K. Sun et al., Weak solvation effects and molecular-rich layers induced water-poor helmholtz layers boost highly stable Zn anode. Energy Storage Mater. **73**, 103856 (2024). 10.1016/j.ensm.2024.103856

[268] J. Zhao, Z. Xu, Z. Zhou, S. Xi, Y. Xia et al., A safe flexible self-powered wristband system by integrating defective MnO_2_–X nanosheet-based zinc-ion batteries with perovskite solar cells. ACS Nano **15**(6), 10597–10608 (2021). 10.1021/acsnano.1c03341 34037383

[269] J. Zhao, W. Wu, X. Jia, T. Xia, Q. Li et al., High-value utilization of biomass waste: from garbage floating on the ocean to high-performance rechargeable Zn–MnO_2_ batteries with superior safety. J. Mater. Chem. A **8**(35), 18198–18206 (2020). 10.1039/d0ta05926a

[270] Z. Tian, Z. Sun, Y. Shao, L. Gao, R. Huang et al., Ultrafast rechargeable Zn micro-batteries endowing a wearable solar charging system with high overall efficiency. Energy Environ. Sci. **14**(3), 1602–1611 (2021). 10.1039/d0ee03623d

[271] S. Saifi, X. Xiao, S. Cheng, H. Guo, J. Zhang et al., An ultraflexible energy harvesting-storage system for wearable applications. Nat. Commun. **15**(1), 6546 (2024). 10.1038/s41467-024-50894-w PMC11297324 39095398

[272] J. Zeng, L. Dong, L. Sun, W. Wang, Y. Zhou et al., Printable zinc-ion hybrid micro-capacitors for flexible self-powered integrated units. Nano-Micro Lett. **13**(1), 19 (2020). 10.1007/s40820-020-00546-7 PMC8187672 34138202

[273] M. Chen, X. Guo, B. Farhadi, X. Guo, Y. Zhu et al., Highest overall conversion efficiency for portable thin-film zinc-ion batteries photorecharged by flexible perovskite solar cells. Chem. Eng. J. **518**, 164711 (2025). 10.1016/j.cej.2025.164711

[274] J. Bi, J. Zhang, P. Giannakou, T. Wickramanayake, X. Yao et al., A highly integrated flexible photo-rechargeable system based on stable ultrahigh-rate quasi-solid-state zinc-ion micro-batteries and perovskite solar cells. Energy Storage Mater. **51**, 239–248 (2022). 10.1016/j.ensm.2022.06.043

[275] P. Chen, T.-T. Li, Y.-B. Yang, G.-R. Li, X.-P. Gao, Coupling aqueous zinc batteries and perovskite solar cells for simultaneous energy harvest, conversion and storage. Nat. Commun. **13**(1), 64 (2022). 10.1038/s41467-021-27791-7 PMC8748727 35013265

[276] H. Liu, P. Wu, R. Wang, H. Meng, Y. Zhang et al., A photo-rechargeable aqueous zinc–tellurium battery enabled by the janus-jointed perovskite/te photocathode. ACS Nano **17**(2), 1560–1569 (2023). 10.1021/acsnano.2c10762 36622820

